# Human Bone Marrow Organoids for Disease Modeling, Discovery, and Validation of Therapeutic Targets in Hematologic Malignancies

**DOI:** 10.1158/2159-8290.CD-22-0199

**Published:** 2022-11-09

**Authors:** Abdullah O. Khan, Antonio Rodriguez-Romera, Jasmeet S. Reyat, Aude-Anais Olijnik, Michela Colombo, Guanlin Wang, Wei Xiong Wen, Nikolaos Sousos, Lauren C. Murphy, Beata Grygielska, Gina Perrella, Christopher B. Mahony, Rebecca E. Ling, Natalina E. Elliott, Christina Simoglou Karali, Andrew P. Stone, Samuel Kemble, Emily A. Cutler, Adele K. Fielding, Adam P. Croft, David Bassett, Gowsihan Poologasundarampillai, Anindita Roy, Sarah Gooding, Julie Rayes, Kellie R. Machlus, Bethan Psaila

**Affiliations:** 1 Institute of Cardiovascular Sciences, College of Medical and Dental Sciences, University of Birmingham, Vincent Drive, Birmingham, United Kingdom.; 2 MRC Weatherall Institute of Molecular Medicine, Radcliffe Department of Medicine and National Institute of Health Research (NIHR) Oxford Biomedical Research Centre, University of Oxford, Oxford, United Kingdom.; 3 Centre for Computational Biology, MRC Weatherall Institute of Molecular Medicine, University of Oxford, Oxford, United Kingdom.; 4 Cancer and Haematology Centre, Churchill Hospital, Oxford University Hospitals NHS Foundation Trust, Oxford, United Kingdom.; 5 Rheumatology Research Group, Institute of Inflammation and Ageing, College of Medical and Dental Sciences, University of Birmingham, Birmingham, United Kingdom.; 6 MRC Weatherall Institute of Molecular Medicine, Department of Paediatrics and National Institute of Health Research (NIHR) Oxford Biomedical Research Centre, University of Oxford, Oxford, United Kingdom.; 7 Vascular Biology Program, Boston Children's Hospital, Department of Surgery, Harvard Medical School, Boston, Massachusetts.; 8 University College London Cancer Institute, London, United Kingdom.; 9 Healthcare Technologies Institute, School of Chemical Engineering, University of Birmingham, Birmingham, United Kingdom.; 10 School of Dentistry, Institute of Clinical Sciences, University of Birmingham, Birmingham, United Kingdom.

## Abstract

A lack of models that recapitulate the complexity of human bone marrow has hampered mechanistic studies of normal and malignant hematopoiesis and the validation of novel therapies. Here, we describe a step-wise, directed-differentiation protocol in which organoids are generated from induced pluripotent stem cells committed to mesenchymal, endothelial, and hematopoietic lineages. These 3D structures capture key features of human bone marrow—stroma, lumen-forming sinusoids, and myeloid cells including proplatelet-forming megakaryocytes. The organoids supported the engraftment and survival of cells from patients with blood malignancies, including cancer types notoriously difficult to maintain *ex vivo*. Fibrosis of the organoid occurred following TGFβ stimulation and engraftment with myelofibrosis but not healthy donor–derived cells, validating this platform as a powerful tool for studies of malignant cells and their interactions within a human bone marrow–like milieu. This enabling technology is likely to accelerate the discovery and prioritization of novel targets for bone marrow disorders and blood cancers.

**Significance:**

We present a human bone marrow organoid that supports the growth of primary cells from patients with myeloid and lymphoid blood cancers. This model allows for mechanistic studies of blood cancers in the context of their microenvironment and provides a much-needed *ex vivo* tool for the prioritization of new therapeutics.

*See related commentary by Derecka and Crispino, p. 263.*

*This article is highlighted in the In This Issue feature, p. 247*

## Teaser

Development of human bone marrow organoids that capture key cellular, molecular, and architectural features of hematopoietic tissue enables disease modeling and target screening using primary cells from patients with myeloid and lymphoid blood cancers.

## INTRODUCTION

The specialized bone marrow microenvironment maintains and regulates hematopoiesis, enabling an adequate supply of blood cells to meet changing physiologic requirements throughout life. Perturbations in the bone marrow hematopoietic niche contribute to the initiation and propagation of hematologic malignancies. In addition, the stromal remodeling that occurs as a consequence of blood cancers contributes to bone marrow failure (1–4). Modeling bone marrow dysfunction is challenging, particularly in the context of human diseases. *In vitro* studies are often limited to 2D systems and simple cocultures, in which the relevant cell types are absent, and many human diseases are inadequately reproduced by mouse models. Patient-derived xenografts have been used to model disease and validate targets *in vivo*, but some malignancies and hematologic cell subtypes do not engraft well, even when humanized murine models are used (5–11).

Advances have been made in modeling certain marrow components on “biochips” (12–16), but the lack of specialized stroma, vascularization, and active blood cell generation remains a limitation with these methods. Coculture with bone marrow mesenchymal stromal cells (MSC) can support hematopoietic cell growth, either in 2D or in 3D with the addition of extracellular matrix support (17–19). Although beneficial, these approaches are limited in terms of the elements of the bone marrow that are incorporated, as well as their scalability and reproducibility due to the limited availability and interdonor variability of primary bone marrow MSCs. Improved *in vitro* systems are therefore required to enable more detailed mechanistic studies of human hematopoiesis, and to allow for the functional interrogation of the pathways and cross-talk that drive bone marrow malignancies.

The development and application of organoids—self-organizing, 3D, living multilineage structures—have the potential to facilitate translational research by enabling genetic screens and pharmacologic modulation of disease pathobiology (20). Our goal was to generate a vascularized human bone marrow–like organoid that contains key hematopoietic niche elements and supports active endogenous hematopoiesis, as well as the growth and survival of hematopoietic cells from adult donors, including malignant cell types that are difficult to grow and study *ex vivo*. Such a system would offer a scalable and highly manipulable human model for mechanistic studies and drug development and, importantly, may reduce dependence on animal models.

To achieve this, we optimized a protocol in which human induced pluripotent stem cells (iPSC) generate mesenchymal elements, myeloid cells, and “sinusoidal-like” vasculature in a format that resembles the cellular, molecular, and spatial architecture of myelopoietic bone marrow. We confirmed the homology of these organoids to human bone marrow using multimodal imaging approaches and single-cell RNA sequencing (scRNA-seq). Crucially, in addition to modeling physiologic hematopoietic cell–niche interactions, we showed that the organoids supported the engraftment and survival of healthy and malignant hematopoietic cells from human donors, and enabled the screening of inhibitors of bone marrow fibrosis, a complication that occurs in patients with certain blood cancers and is associated with poor prognosis.

This platform addresses a long-standing need for 3D human bone marrow models for translational research in which both niche and hematopoietic components are species and cell context specific and creates a dynamic platform for high-throughput drug screening and studies aiming to understand disease pathways.

## RESULTS

### Mixed-Matrix Hydrogels Containing Matrigel and Type I and IV Collagens Are Optimal for the Production of Vascularized, Myelopoietic Organoids

**Figure 1.**
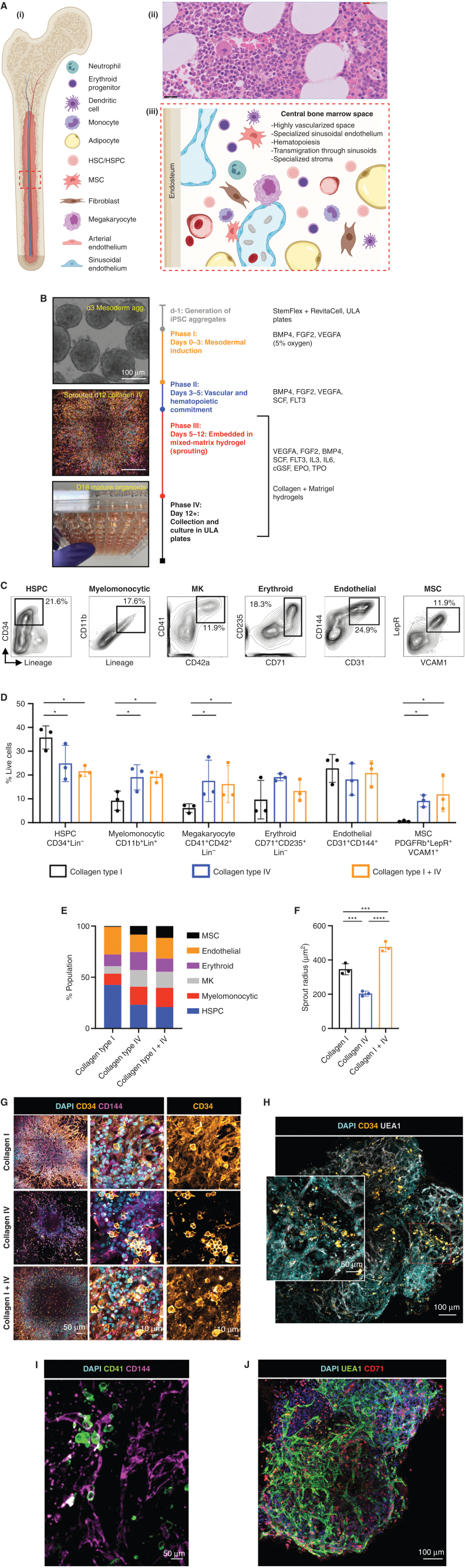
Mixed-matrix hydrogels containing Matrigel and type I and IV collagens are optimal for production of vascularized, myelopoietic organoids. **A,** (i) Central bone marrow is a complex tissue including MSC, endothelial, hematopoietic stem and progenitor cell (HSC/HSPCs), and myeloid and lymphoid subsets. (ii) Hematoxylin and eosin–stained section and (iii) model of human bone marrow highlighting the diverse hematopoietic and stromal cell types (created using BioRender.com). **B,** Differentiation workflow, in which iPSC aggregates undergo mesodermal induction (days 0–3) and commitment to hematopoietic and vascular lineages (days 3–5). Cell aggregates are then embedded in mixed-matrix hydrogels comprised of Matrigel and collagen I, collagen IV, or collagen I + IV mix at a 40:60 ratio to support vascular sprouting. Key media components are listed for each phase. Gating strategy (**C**) and quantification (**D**) of stromal and hematopoietic cell types in day 18 organoids supported by Matrigel + collagen type I–only, collagen IV–only, and collagen I + IV hydrogels. MK, megakaryocyte. **E,** Distribution of lineages as fractions of the whole organoid population. **F,** Radius of endothelial sprouts. **G,** Sprouting day 12 organoids immunostained for nuclei (DAPI), CD34, and CD144 (VE-cadherin). **H–J,** Whole organoid Z-stack imaging acquired at day 18 showing CD34^+^ HSPCs and UEA1^+^ vessels that are negative for CD34 (**H**), CD41^+^ megakaryocytes (**I**), and CD71^+^ erythroid cells dispersed throughout the organoids and associating with CD144^+^/UEA1^+^ vasculature (**J**). *, *P* < 0.05; ***, *P* < 0.001 for one-way ANOVA with multiple comparisons [Fisher least significant difference (LSD)]; *n* = 3 for endothelial sprout radius measurements. Two-way ANOVA with multiple comparisons (Fisher LSD); *n* = 3 (3 independent differentiations, 15 pooled organoids each) for flow cytometry analysis. Representative images are shown. See also Supplementary Fig. S1.

To mimic the central bone marrow space (Fig. 1A), we devised a four-stage workflow to generate mesenchymal, vascular, and myelopoietic marrow components. Human iPSCs were allowed to form nonadherent mesodermal aggregates (phase I, days 0–3; Fig. 1B) before commitment to vascular and hematopoietic lineages (phase II, days 3–5; Fig. 1B). The resulting cell aggregates were then embedded in mixed collagen–Matrigel hydrogels to induce vascular sprouting (phase III, days 5–12; Fig. 1B). At day 12, sprouts were collected individually and cultured to form bone marrow organoids in 96-well ultralow attachment (ULA) plates (phase IV, day 12 onward; Fig. 1B).

Hydrogels comprising Matrigel plus type I collagen have previously been used to support the formation of iPSC-derived blood vessel organoids (21). However, type IV collagen is more permissive than type I collagen for myeloid and megakaryocyte maturation in standard 2D *in vitro* culture systems (22, 23). We therefore compared hydrogels containing type I and/or type IV collagen plus Matrigel for the generation of stromal, endothelial, and myeloid lineages. Distinct immunophenotypic hematopoietic stem and progenitor cell (HSPC; CD34^+^ Lin^−^), myelomonocytic (CD34^−^ CD11b^+^ Lin^+^), megakaryocyte (CD34^−^ Lin^−^ CD41^+^ CD42b^+^), endothelial (CD31^+^ CD144^+^), erythroid (CD34^−^ Lin^−^ CD71^+^ CD235a^+^), and MSC (CD31^−^ CD140b^+^ VCAM1^+^ LepR^+^) populations were detected when organoids were digested at day 18 of differentiation (Fig. 1C–E). Organoids developed in collagen type I–only hydrogels contained the highest fraction of HSPCs but a low proportion of myelomonocytic cells and megakaryocytes, and no MSCs, whereas collagen type IV and collagen type I + IV hydrogel–derived organoids yielded significantly larger myeloid and MSC populations, indicating that the addition of collagen IV created more favorable conditions for multilineage differentiation (Fig. 1D and E; Supplementary Fig. S1A).

The hematopoietic niche contains a dense network of sinusoidal vessels, and specialized sinusoidal endothelium regulates stem cell self-renewal and differentiation, progenitor maturation, and platelet generation (24, 25). To assess vascularization of the organoids, we measured the degree of endothelial sprouting. Vascular sprout radii were significantly smaller in collagen type IV–only hydrogels compared with collagen types I–only and I + IV (average radius of 203.6 ± 14.61 μm vs. 345.9 ± 32.11 μm vs. 476.3 ± 28.82 μm, respectively; Fig. 1F), and the density of CD34 and CD144 [Vascular Endothelial (VE)-Cadherin]–positive sprouts was significantly lower in collagen IV–only hydrogels (Fig. 1G). To assess the vascularization and cellular architecture of the whole organoid in 3D, organoids were mounted in agarose and cleared with Ethyl Cinnamate for confocal Z-stack imaging. This revealed an elaborate network of UAE1^+^ CD144^+^ vessels throughout the collagen I + IV–derived organoids (Fig. 1H–J). In addition to CD34^+^ HSPCs (Fig. 1H), CD41^+^ megakaryocytes (Fig. 1I) and CD71^+^ erythroid cells (Fig. 1J) were distributed throughout the organoid volume and closely associated with the endothelium, as occurs in the native bone marrow.

### Addition of VEGFC Induces Specialization of Organoid Vasculature to a Bone Marrow Sinusoid-like Phenotype

**Figure 2.**
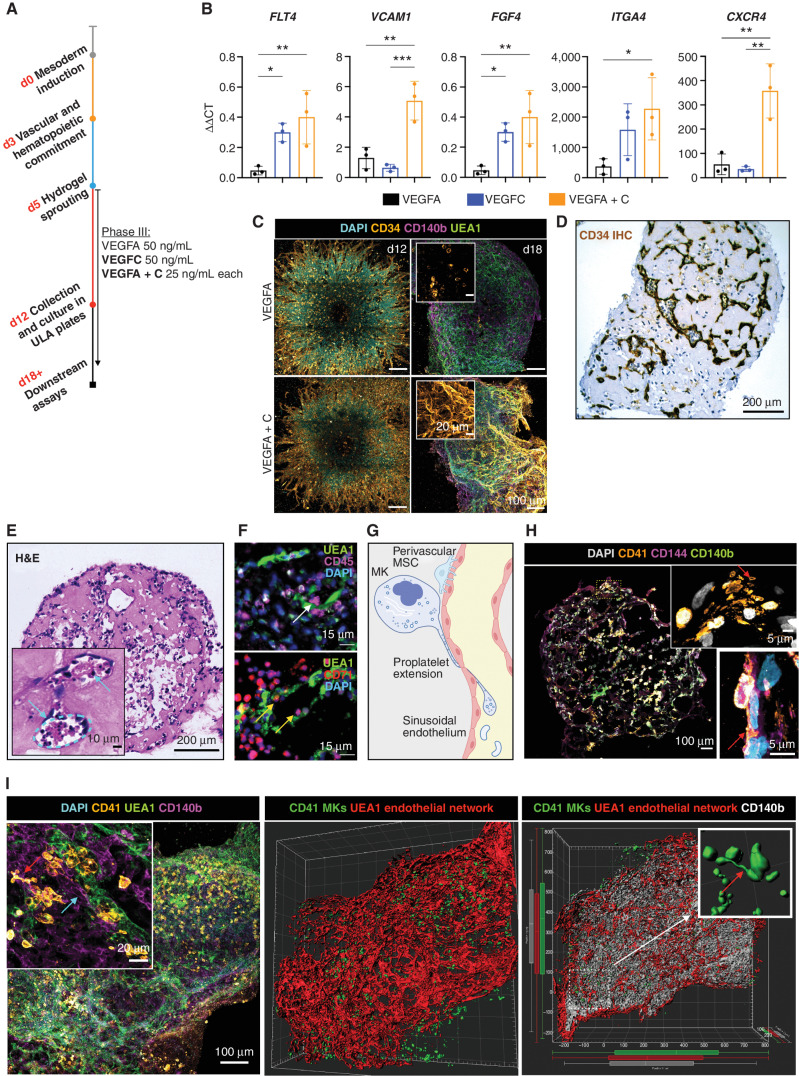
The addition of VEGFC induces specialization of organoid vasculature to a bone marrow sinusoid-like phenotype. **A,** In the sprouting phase of differentiation (D5) in hydrogels, organoids were supplemented with either VEGFA or VEGFC, or both VEGFA and VEGFC. **B,** mRNA expression of canonical cell-surface receptors, growth factors, and adhesion markers of bone marrow sinusoidal endothelium in VEGFA-, VEGFC-, and VEGFA + C–treated samples. ΔΔC_t_ values relative to housekeeping (GAPDH) and undifferentiated iPSCs shown. Each datapoint represents 15 organoids; 3 independent differentiations shown. *, *P* < 0.05; **, *P* < 0.01; ***, *P* < 0.001 for one-way ANOVA with multiple comparisons (Fisher least significant difference). **C,** CD34^+^ sprouting vessels at day 12 in both VEGFA and VEGFA + C conditions. At day 18, vessels were CD34 positive in VEGFA + C organoids but negative in VEGFA-only organoids. **D** and **E,** IHC staining for CD34 (**D**) and hematoxylin and eosin (H&E) staining of formalin-fixed, paraffin-embedded VEGFA + C organoid sections (**E**), with inset showing lumen-forming vessels containing hematopoietic cells (blue arrows). **F,** Immunofluorescence staining of paraffin-embedded sections of VEGFA + C organoids showing CD45^+^ hematopoietic (white arrow) and CD71^+^ erythroid cells (yellow arrows) migrating into the UAE1^+^ vessel lumen. **G,** Schematic demonstrating the process of proplatelet formation by megakaryocytes (MK; image created using BioRender.com). **H,** Whole organoid image showing CD140b^+^ MSCs surrounding CD144^+^ vessels with CD41^+^ megakaryocytes. Insets show megakaryocytes extending proplatelet protrusions into vessel lumen (red arrows). Top inset shows CD41^+^ platelet-like particles within vessel lumen. **I,** Confocal imaging and 3D render of whole-mount VEGFA + C organoids showing CD41^+^ megakaryocytes (red arrow) closely associating with the UEA1^+^ vessel network that is invested with CD140b^+^ fibroblast/MSCs (blue arrow; left and center image). Inset (right) shows 3D rendered megakaryocytes displaying proplatelet formation (red arrow).

Having determined that collagen I + IV Matrigel hydrogels enabled multilineage differentiation and the generation of a 3D vascular network, we sought to determine the optimal balance of endothelial growth factor support to generate organoid vasculature that resembles bone marrow sinusoids (24, 25). VEGFA is a key regulator of blood vessel formation in health and disease, acting via the VEGF receptors VEGFR1 and 2 (26). Bone marrow sinusoidal endothelial cells express VEGFR1 and 2 as well as VEGFR3 (27), and VEGFC—the main ligand for VEGFR3—was recently demonstrated to maintain the perivascular hematopoietic niche in murine bone marrow (28). We therefore tested the effect of adding VEGFC to the vascular sprouting phase (day 5; Fig. 2A). The addition of VEGFC significantly increased the expression of *FLT4* (encoding VEGFR3) in the organoids, as well as HSPC adhesion molecules (*VCAM1* and *ITGA4*) and HSPC-supporting growth factors and chemotactic cytokines (*CXCR4* and *FGF4*; Fig. 2B). Supplementation with VEGFC in addition to VEGFA also induced retention of CD34 expression on organoid vessels, similar to native adult bone marrow vessels, whereas organoids stimulated with VEGFA alone expressed CD34 during vessel sprouting (days 5–12; Fig. 1G) but lost CD34 expression by day 18 (Figs. 1H and 2C).

Organoid vessels formed clear lumens, containing extravasating hematopoietic cells (Fig. 2D and E), confirmed by multiplexed immunostaining to identify CD45^+^ and CD71^+^ hematopoietic cells within the lumen of UEA1^+^ sinusoidal vessels (Fig. 2F). A hallmark of the bone marrow perivascular niche *in vivo* is the close association of megakaryocytes with sinusoidal endothelium, where they extend long, beaded proplatelet extensions into the vessel lumen (Fig. 2G). These extensions subsequently generate platelet buds under shear forces (Fig. 2G; ref. 29). Cryosections of the bone marrow organoids stained for CD41^+^ MKs, CD144^+^ endothelium, and CD140b^+^ MSCs/fibroblasts demonstrated classic proplatelet protrusions in association with the vessels (Fig. 2H and I), with remarkable similarity to previously published *in vivo* images of thrombopoiesis occurring in calvarial bone marrow (29). Volumetric imaging of whole-mount organoids demonstrated that these cells were organized in 3D in an extensive endothelial network invested with perivascular CD140b^+^ fibroblasts/MSCs. A higher number of megakaryocytes were observed in close proximity (5 μm) to vessels in VEGFA + C–stimulated organoids than in VEGFA-only organoids (Supplementary Fig. S1B), consistent with the increased expression of chemotactic factors and adhesion proteins in VEGFA + C organoids (Fig. 2B). Together, these data indicate that the addition of VEGFC improved vascularization of the organoids and hematopoietic support.

### scRNA-seq Confirmed that Hematopoietic and Stromal Cell Lineages within Organoids Have Transcriptional Homology to Human Hematopoietic Tissues

**Figure 3.**
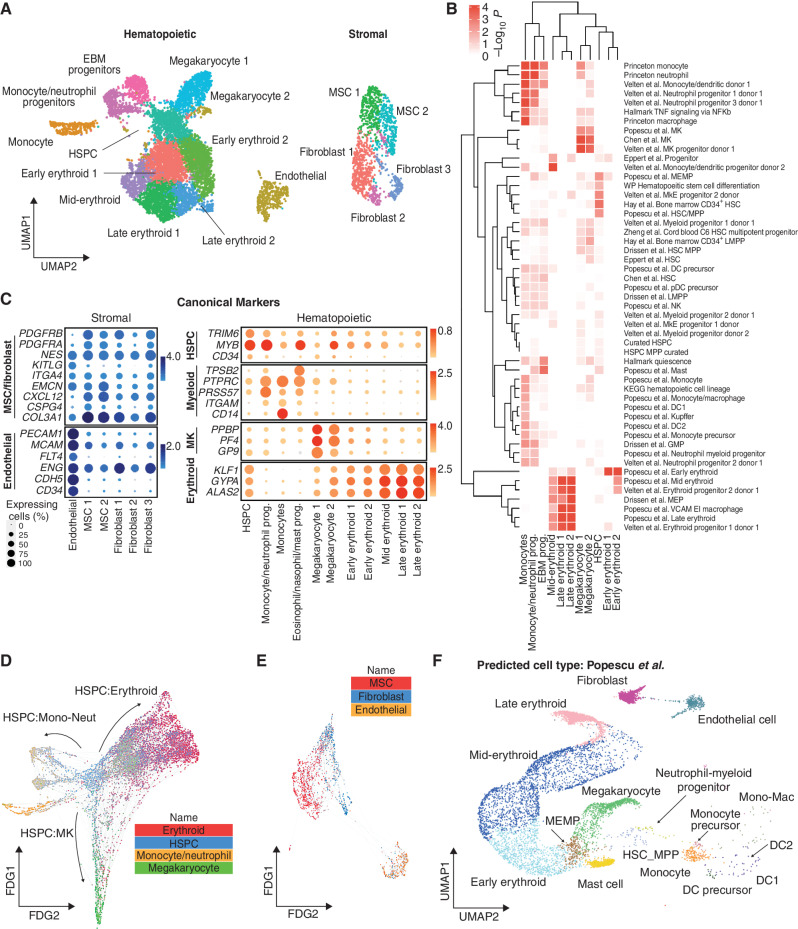
scRNA-seq confirmed that hematopoietic and stromal cell lineages within organoids showed transcriptional similarity to human hematopoietic tissues. **A,** Uniform manifold approximation and projection (UMAP) plot showing annotated cell clusters. **B,** Gene set enrichment analysis of differentially expressed genes for each cluster using a curated set of 64 hematopoietic lineage gene sets. DC, dendritic cell; GMP, granulocyte-macrophage progenitor; HSC, hematopoietic stem cell; KEGG, Kyoto Encyclopedia of Genes and Genomes; LMPP, lymphoid-primed multipotent progenitor; MEMP, megakaryocyte-erythroid-mast cell progenitor; MK, megakaryocyte; MkE, megakaryocyte-erythroid; MPP, multipotent progenitor; NK, natural killer cell; pDC, plasmacytoid DC; prog., progenitor; VCAM EI, VCAM erythroblastic island; WP, WikiPathways. **C,** Expression of canonical stromal and hematopoietic cell genes for each of the annotated clusters. The color scale represents the average level of expression, and the circle size shows the percentage of cells within each cluster in which expression was detected for each gene. **D** and **E,** FDG showing differentiation trajectories for hematopoietic (**D**) and stromal (**E**) compartments, superimposed with expression scores of lineage signature gene sets. **F,** Organoid cells projected onto a published dataset of human hematopoietic and stromal cells using the Symphony package (33). Mono-Mac, monocyte-macrophage. See also Supplementary Figs. 2 and 3 and Supplementary Tables S1 and S2.

To compare the cell types and molecular profiles of the organoids to human hematopoietic tissues, scRNA-seq was performed on a total of 26,648 cells from 3 independent organoid differentiations using VEGFA-only and VEGFA + C protocols. After quality control (see Methods), 19,506 cells (10,205 from VEGFA only and 9,301 from VEGFA + C) were included in downstream analyses. Distinct populations of key hematopoietic and stromal cell subtypes were identified, including HSPC, erythroid, neutrophil, monocyte, megakaryocyte, eosinophil/basophil/mast (EBM), fibroblast, endothelial cell, and MSC (Fig. 3A; Supplementary Table S1), annotated using gene set enrichment analysis with a curated list of 64 published gene sets (Fig. 3B; Supplementary Table S2; refs. 30, 31), as well as the expression of canonical marker genes (Fig. 3C). Erythroid, megakaryocytic, monocytic/neutrophil, and EBM populations demonstrated expression of *GYPA* and *KLF1*, *PF4* and *PPBP*, *CD14* and *RUNX1*, and *TPSB2* and *KIT*, respectively (Fig. 3C).

Within stromal cell subsets, expression of *COL3A1*, platelet-derived growth factors (*PDGFRA*/*B*), and the key HSPC niche factor *CXCL12* (SDF-1) was observed in fibroblasts and MSCs, with particularly high expression of *CXCL12* in the MSCs, suggesting a key role for MSCs in homing and maintenance of HSPCs to perivascular regions of the organoids (Fig. 3C; Supplementary Fig. S2A and S2B). High expression of *PECAM1*, *CDH5*, and *ENG* was detected in endothelial cells, confirming vascular specification (Fig. 3C; Supplementary Fig. S2A and S2B). The relative frequency of cell types in VEGFA-only organoids was broadly similar to that of VEGFA + C organoids, with a relative increase in abundance of endothelial and erythroid cells captured from organoids grown in the VEGFA + C condition (Supplementary Fig. S2C).

Trajectory analysis using a force-directed graph (FDG) showed that the organoids contained cells recapitulating the three main routes of hematopoietic myeloid differentiation (Fig. 3D; Supplementary Fig. S2D), similar to observations in human bone marrow (31). FDG analysis of the stromal cell populations showed independent routes of differentiation for endothelium and MSCs/fibroblasts as expected (Fig. 3E; Supplementary Fig. S2E).

To determine the transcriptional similarity of the bone marrow organoid to native human hematopoietic tissues, organoid scRNA-seq data were projected onto published single-cell datasets of cells isolated from adult human bone marrow (31, 32) and from fetal liver and fetal bone marrow (33) using the Symphony package (34). This revealed an extensive overlap of organoid-derived cells with HSPCs, myeloid subsets, fibroblasts/MSCs, and endothelial cell types, with the predicted cell types matching the cluster annotations (Fig. 3F; Supplementary Fig. S3A–S3C).

**Figure 4.**
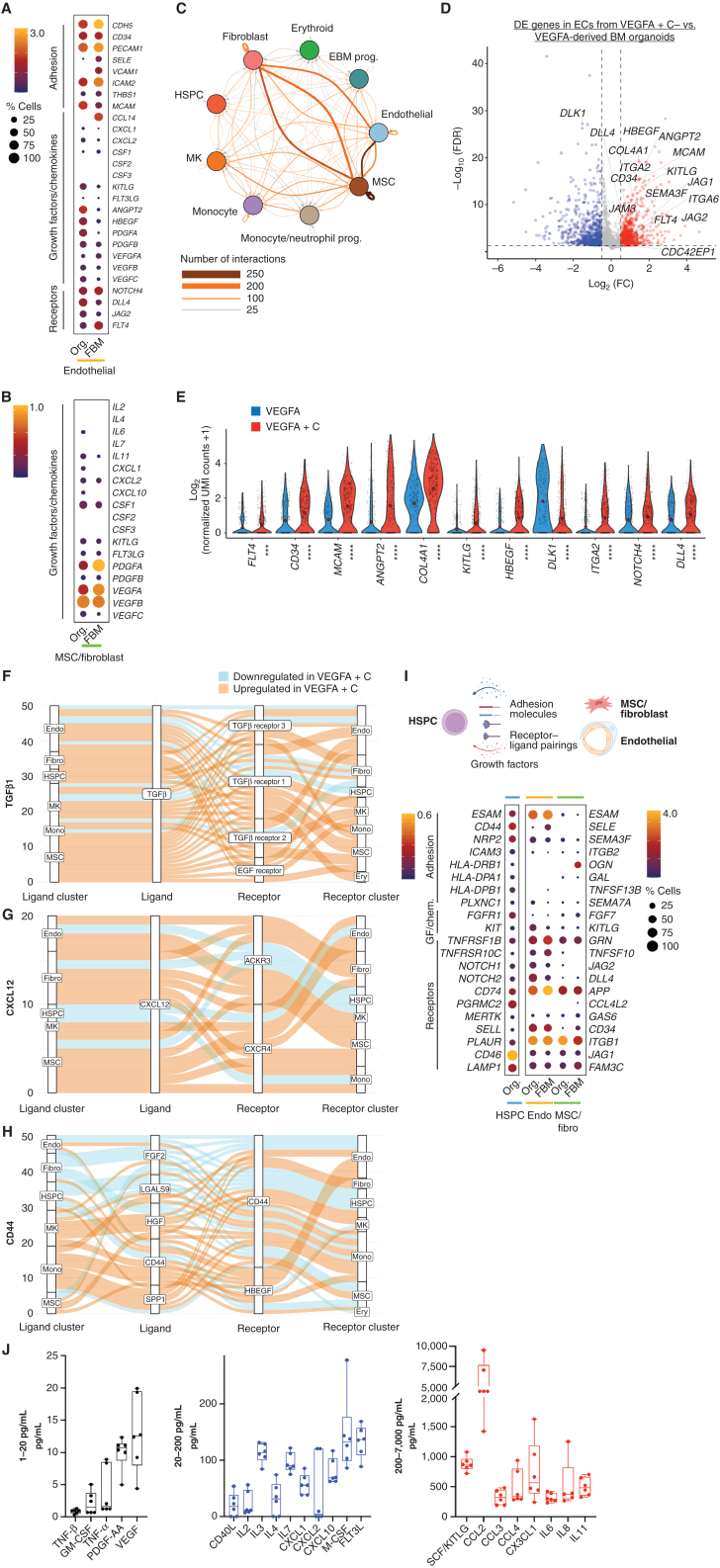
Endothelial cells (EC), fibroblasts, and MSCs from organoid stroma support hematopoiesis with increased hematopoietic support from VEGFA + C–stimulated vasculature. **A,** Comparison of expression of key receptors, adhesion proteins, and growth factors and chemokines in ECs from bone marrow organoids (Org.) and human fetal bone marrow (FBM). **B,** Comparison of expression of growth factors and chemokines in MSCs from organoids and FBM. Size of dots represents percentage of expressing cells, and color density indicates level of expression. **C,** Total predicted ligand–receptor interactions across clusters as predicted by CellPhoneDB (V2.0), showing extensive autocrine and paracrine interactions across BM organoid. MK, megakaryocyte; prog., progenitor. **D,** Volcano plot showing significantly differentially expressed (DE) genes in ECs from VEGFA + C versus VEGFA-only organoids (801 significantly upregulated and 700 significantly downregulated genes, *P* < 0.05, log_2_FC > 0.5 or −0.5). **E,** Violin plots showing key hematopoietic support factors and markers of bone marrow sinusoidal endothelium in ECs of VEGFA + C and VEGFA-only organoids. *P* values are indicated below *x*-axis labels, and mean value is indicated on violin plots. ***, *P* < 0.001; ****, *P* < 0.0001 for pairwise comparison Wilcox test applied (FDR). UMI, unique molecular identifier. **F–H,** Sankey plots comparing *TGFβ1*-mediated (**F**), *CXCL12*-mediated (**G**), and *CD44*-mediated (**H**) interactions in VEGFA + C versus VEGFA-stimulated organoids. Endo, endothelial; Ery, erythroid; Fibro, fibroblast; Mono, monocyte. **I,** Expression of interacting receptor–ligand pairs between organoid HSPCs and cognate partner in organoid or FBM ECs (endo) and MSCs/fibroblasts with percentage of expressing cells and level of expression shown. **J,** Hematopoietic cytokines/growth factors produced by bone marrow organoids, measured by Luminex assay. Each datapoint represents supernatant pooled from 16 separately generated organoids. See also Supplementary Figs. 4–7.

To explore the transcriptional similarity between the stromal support elements of the organoids with that of native human bone marrow in more detail, we extracted the stromal cell populations from a recently published study of fetal bone marrow. This provided the first comprehensive annotation of stromal cell subsets in human bone marrow (35), including endothelial, MSC, fibroblast, and osteochondral lineage subsets. Integration and unsupervised clustering showed a close approximation of organoid MSCs/fibroblasts (*n* = 687) and endothelial cells (*n* = 766) with the relevant cell clusters extracted from the human bone marrow dataset (endothelial, *n* = 766 and MSCs/fibroblasts, *n* = 687 cells; Supplementary Fig. S4A–S4C). As expected, osteolineage cells, chondrocytes, and smooth muscle and Schwann cells present in fetal bone marrow were absent from the organoids, and the distinct populations of sinusoidal and nonsinusoidal “tip” endothelial cells were also not detected. However, expression of adhesion proteins, cytokines, and hematopoietic support factors was very similar between organoid and bone marrow cells, including *CD34*, *PECAM*, *KITLG*, *FLT3LG*, and *ANGPT2* for endothelial cells (Fig. 4A) and *KITLG*, *PDGF*, and *VEGF* family members for MSCs/fibroblasts (Fig. 4B), confirming that the iPSC bone marrow organoid–derived stromal cells are highly homologous to their native bone marrow counterparts.

### Bone Marrow Organoids Recapitulate Cellular and Molecular Cross-talk between Hematopoietic, Endothelial, and Stromal Cells

To investigate the cellular and molecular interactions between hematopoietic, endothelial, and stromal cell subtypes within the organoids, we mapped the expression of interacting receptor and ligand pairs across clusters. Complex communication networks were detected, both within and between hematopoietic and stromal cell compartments (Fig. 4C). Strong autocrine and paracrine interactions were predicted between MSCs, fibroblasts, endothelial cells, and monocytes, HSPCs, and megakaryocytes, whereas erythroid cells showed weak interactions (Fig. 4C; Supplementary Fig. S5A–S5C).

Numerous interacting receptor–ligand partners were detected between megakaryocytes and endothelial cells (Supplementary Figs. S5B and S6A), and megakaryocytes with MSCs (Supplementary Fig. S6B), indicating bidirectional regulatory interactions between these cell types. These included *NOTCH1–JAG1/2, FLT4–PDGFC, ANGPT2–TEK*, and *FLT1–VEGFB* for endothelium:megakaryocytes (Supplementary Figs. S5B and S6A), and *NOTCH1–DLL4, KIT–KITLG, FGF2–CD44, SELP–CD34*, and *VEGFRA–FLT1* for megakaryocytes:endothelial cells (Supplementary Fig. S6A). Interactions between megakaryocytes and MSCs were dominated by growth factors produced by megakaryocytes, including *TGFB*, *PDGF*, *FGF*, and *VEGF* family members and their cognate receptors (Supplementary Fig. S6B).

Similarly, monocytes and endothelial cells demonstrated abundant interacting partners indicative of regulatory interactions, including *TNF–NOTCH1, JAG1/2–NOTCH, SIRPA–CD47*, and *LGALS9, ICAM, VEGF* family members with cognate receptors (Supplementary Figs. S5C and S6C). Significantly interacting partners between monocytes and MSCs/fibroblasts included *CXCL12–CXCR4, ICAM1–aXb2*, *SPP1–CD44*, IL1 and IL16, and hepatocyte and fibroblast growth factors with their respective binding partners (Supplementary Fig. S6D).

Although a high number of regulatory interactions between hematopoietic and stromal compartments were detected, interactions between the stromal cell subsets (endothelial cells:MSCs:fibroblasts) were particularly strong (Fig. 4C). Significantly interacting partners identified included key regulatory molecules such as *JAG–NOTCH, VEGF, DLL4–NOTCH3, PDGF–PDGFR, ANPT1/2–TEK*, *IL33–IL33R*, *FGF*, and *TGFB* (Supplementary Fig. S6E; refs. 36–38).

To explore the impact of the addition of VEGFC to the differentiation protocol on the phenotype of organoid vasculature, we compared the transcriptomes of endothelial cells captured from VEGFA- and VEGFA + C–derived bone marrow organoids by scRNA-seq. A total of 1,501 genes were significantly differentially expressed between endothelial cells generated with VEGFA only versus VEGFA + C, including 801 upregulated and 700 downregulated genes (*P* < 0.05, log_2_FC > 0.5 or −0.5; Fig. 4D). Canonical markers of bone marrow sinusoidal endothelium were more highly expressed in endothelial cells from VEGFA + C organoids than VEGFA-only organoids, including *FLT4* (VEGFR4), *CD34, MCAM, ANGPT2, COL4A1, COL4A2, ITGA2, CDC42EP1*, and the notch ligand *DLL4* (25, 35), whereas *DLK1*—a negative regulator of hematopoiesis (39)—was significantly lower in VEGFA + C–stimulated organoids than in VEGFA-only (Fig. 4D and E).

In addition to improved hematopoietic support from endothelial cells, key regulatory axes were also upregulated across other cellular subsets in VEGFA + C organoids compared with VEGFA organoids (Fig. 4F–H). TGFβ1 signaling primarily from MSCs, megakaryocytes, and fibroblasts was increased overall in VEGFA + C organoids across the different TGFβ receptors (Fig. 4F). Similarly, CXCL12 signaling via CXCR4 and ACKR3 receptors across both stromal and hematopoietic cell types was also elevated (Fig. 4G), whereas the impact of VEGFC on signaling between CD44 and its binding partners was more mixed (Fig. 4H).

The receptor–ligand communication networks predicted between hematopoietic cells and niche components in the organoid stroma mirrored many of the communication networks that have been reported in native bone marrow (35), including *CD44–SELE, CD74–APP, KIT–KITLG, ICAM3–ITGB2, CD46–JAG1*, and *NOTCH2–DLL4* (Fig. 4I).

Finally, we explored whether the expression of hematopoietic support factors detected in the bone marrow organoid niche cells by scRNA-seq (Supplementary Fig. S7A) could be confirmed at protein level. Organoids generated with VEGFA + C were harvested at day 18, washed, replated in media supplemented with L-Glutamine but no added cytokines or growth factors, and cultured for a further 12 days without any added supplements with 50:50 media changes every 72 hours. In the absence of exogenously supplied cytokines, the organoids secreted multiple hematopoietic factors including SCF/KITLG, CCL2–4, interleukins (IL^2, 3, 4, 6, 7, 8, 11^), PDGF, FLT3L, M-CSF, GM-CSF, and VEGF (Fig. 4J; Supplementary Fig. S7B), confirming that the organoid stroma expresses key growth factors that might endogenously support hematopoiesis.

### Bone Marrow Organoids Model the TGFβ-Induced Bone Marrow Fibrosis That Occurs in Hematologic Cancers and Provide an *Ex Vivo* Platform for Inhibitor Screening

**Figure 5.**
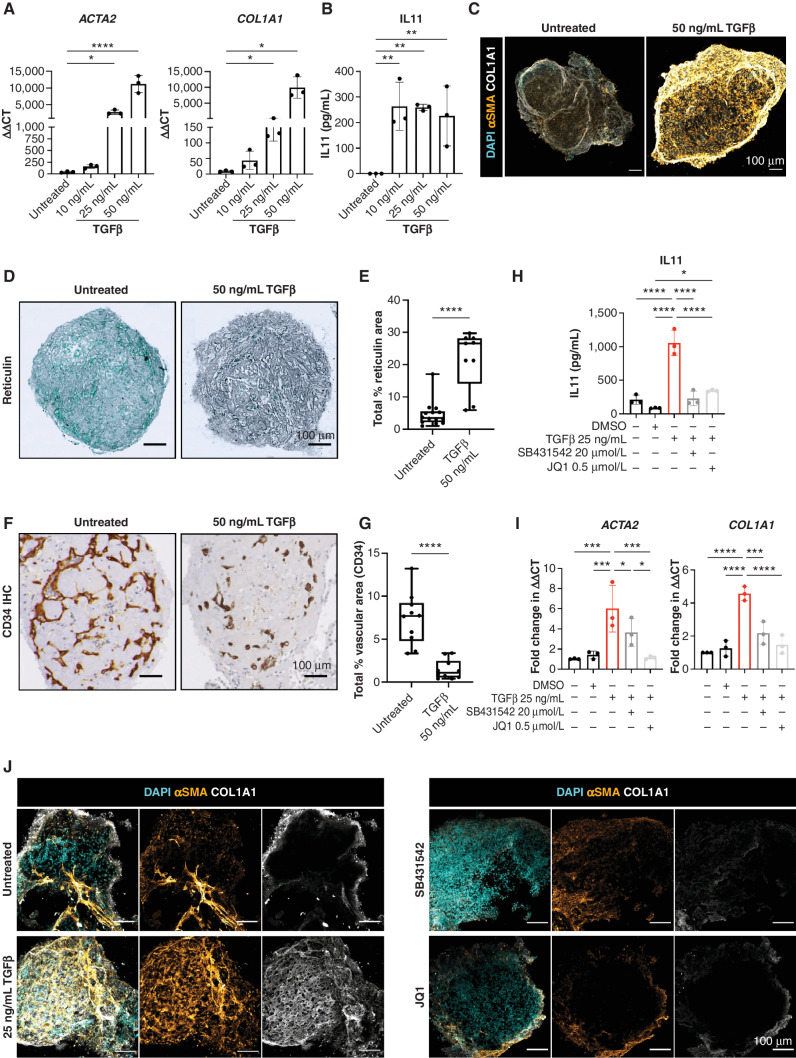
Bone marrow organoids model TGFβ-induced bone marrow fibrosis and enable inhibitor screening. **A,** Organoids were treated with 10, 25, or 50 ng/mL recombinant TGFβ and evaluated by qRT-PCR for expression of *ACTA2* (αSMA) and *COL1A1*, indicators of fibrosis. **B,** Soluble IL11 detected in organoid media following treatment of organoids with TGFβ. **C,** Confocal Z-stack images of whole, untreated, and TGFβ (50 ng/mL)-treated organoids stained for αSMA and COL1A1. **D,** Reticulin staining of formalin-fixed, paraffin-embedded sections of TGFβ-treated organoids versus control. **E,** Measurement of total reticulin stained area in untreated and TGFβ (50 ng/mL)-treated organoids. **F** and **G,** CD34 immunostaining of organoid vessels (**F**) and quantification of total vascular area of organoids with/without TGFβ treatment (**G**). **H** and **I,** Effect of two potential inhibitors of TGFβ-induced fibrosis (SB431542 and JQ1) on IL11 secretion (**H**) and *ACTA2* and *COL1A1* expression (**I**). **J,** αSMA and COL1A1 expression in TGFβ-treated organoids with/without indicated inhibitors. Representative images are shown. *, *P* < 0.01; **, *P* < 0.05; ***, *P* < 0.001; ****, *P* < 0.0001 for one-way ANOVA with multiple comparisons (Fisher least significant difference). *T* tests performed for image analysis of paraffin-embedded sections (reticulin and CD34). *n* = 3 with each repeat comprising 15 organoids pooled from 3 independent differentiations and treatments.

Pathologic hematopoietic niche remodeling occurs in the majority of hematologic malignancies. In certain cancers, particularly myeloproliferative neoplasms, myelodysplasia, acute leukemia, and mast cell neoplasms, bone marrow fibrosis is a major cause of bone marrow failure and morbidity and is associated with a poor prognosis (40). Fibrosis results from the excess production and release of profibrotic cytokines by hematopoietic cells—in particular TGFβ—leading to the deposition of reticulin and collagen fibers by marrow stroma (40–42). To investigate whether the organoids could model pathologic bone marrow fibrosis, we treated organoids with varying doses of TGFβ (10 ng/mL, 25 ng/mL, and 50 ng/mL), which resulted in a dose-dependent increase in the expression of hallmarks of fibrosis, including alpha smooth muscle actin (αSMA [*ACTA2*]) and collagen 1 (*COL1A1*; Fig. 5A), both canonical markers of fibroblast activation (42). A significant increase in soluble IL11 was also observed (Fig. 5B; ref. 43). Collagen deposition within the organoids was markedly increased following TGFβ treatment (Fig. 5C), with pronounced reticulin fibrosis (Fig. 5D and E), recapitulating changes seen in the bone marrow of patients with myelofibrosis. The induction of organoid fibrosis was accompanied by reduced vascularization, suggesting multilineage remodeling as a consequence of TGFβ stimulation, as occurs in adult bone marrow (Fig. 5F and G).

We then explored the utility of this system to test potential inhibitors of fibrosis, selecting two compounds that inhibit pathways currently under investigation in clinical trials for myeloid malignancies—SB431542, an inhibitor of the TGFβ superfamily type I activin receptors, and the BET bromodomain inhibitor JQ1 (44). TGFβ-induced expression of soluble IL11 was completely inhibited by both treatments (Fig. 5H), whereas *ACTA2* and *COL1A1* overexpression was normalized by JQ1 and reduced by 1.5-fold and 2.5-fold, respectively, by SB431542 at the transcript level (Fig. 5I), and this reduction was also evident at the protein level by immunofluorescence imaging (Fig. 5J). Together, these data confirm that bone marrow organoids provide an efficient model of malignant bone marrow fibrosis and enable screening for the efficacy of potential pharmacologic modulators.

### Organoid “Niche Remodeling” and Fibrosis Occurs following Engraftment with Cells from Patients with Myelofibrosis but Not Healthy Donors

Having confirmed substantial homology to native bone marrow, we hypothesized that the organoids may support the engraftment of primary cells from patients with hematologic malignancies, enabling the modeling of cancer–stroma interactions and the possibility of patient-specific cytotoxic screens. Given the fibrosis observed following treatment with TGFβ, and the current lack of adequate *in vitro* and *in vivo* systems for modeling cancer-induced bone marrow fibrosis, we first seeded the organoids with cells from healthy donors and patients with myelofibrosis, and tested the impact of engraftment on the remodeling of the bone marrow organoid “niche.”

**Figure 6.**
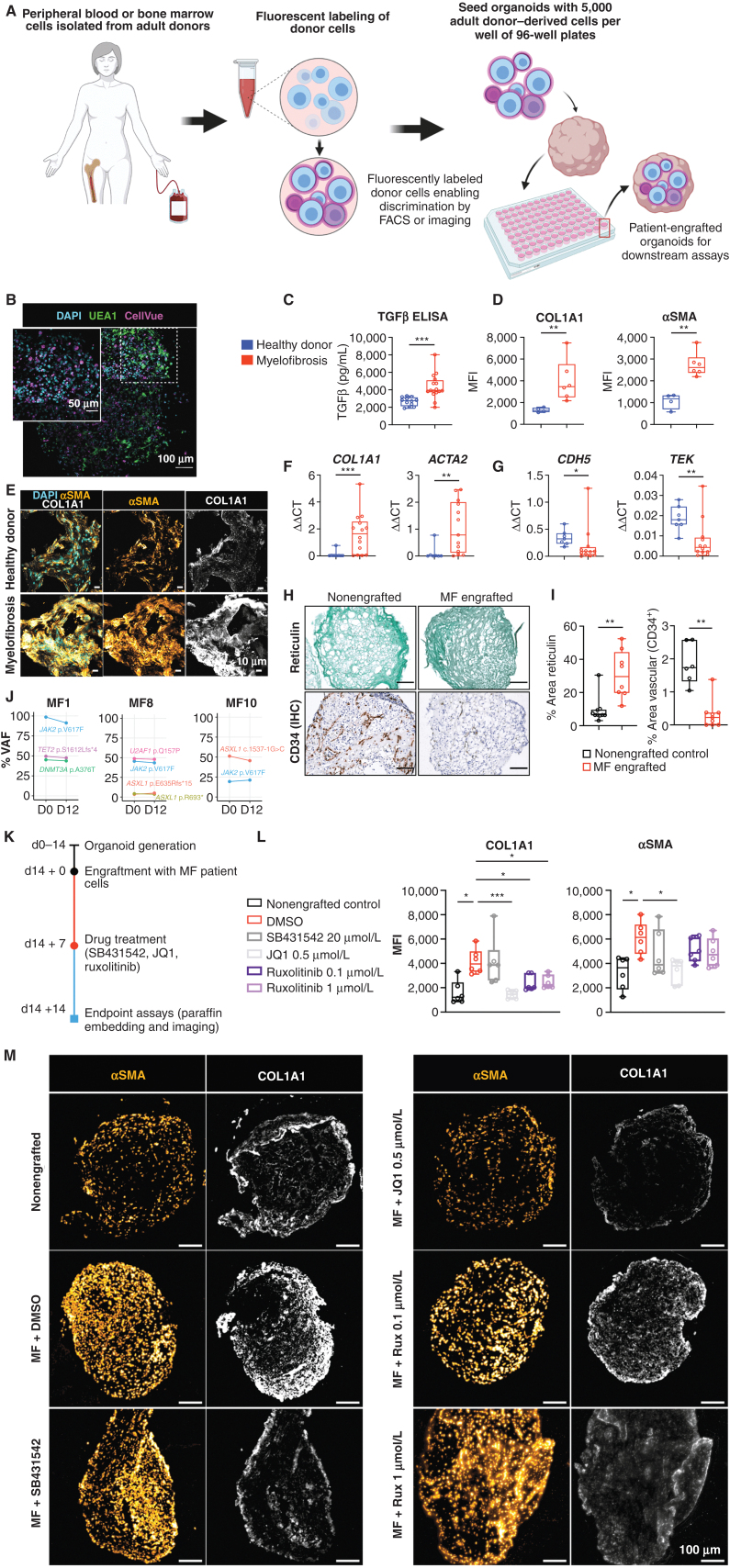
Engraftment of cells from patients with myelofibrosis, but not healthy donors, results in organoid “niche remodeling” and fibrosis. **A,** Cryopreserved peripheral blood or bone marrow cells from healthy donors and patients with blood cancers were fluorescently labeled, and 5,000 donor cells were seeded into each well of a 96-well plate containing individual organoids. Schematic created using BioRender.com. **B,** Maximum-intensity projection of confocal Z-stack of a whole engrafted organoid 72 hours after seeding of the wells with donor cells, indicating donor cells engrafted throughout the volume of the organoids. **C,** Soluble TGFβ in organoids engrafted by cells from patients with myelofibrosis and controls. **D–G,** Comparison of organoids engrafted with healthy donor and myelofibrosis cells for collagen 1 (COL1A1) and αSMA immunofluorescence (**D**) and representative images (**E**), *Col1A1* and *ACTA2* gene expression (**F**), and *CDH5* and *TIE2* expression (**G**).*n* = 4 healthy donors; *n* = 7 myelofibrosis samples for qRT-PCR; *n* = 4 healthy donors; *n* = 6 myelofibrosis samples for imaging and quantification of cryosections. MFI, mean fluorescence intensity. **H** and **I,** Increased reticulin deposition with a concomitant reduction in vascular area in organoids engrafted with myelofibrosis (MF) cells versus nonengrafted control organoids with paired *t* tests; each datapoint corresponds to a single organoid engrafted with cells from 3 donors. **J,** Variant allele frequencies (VAF) of mutations detected by next-generation sequencing of cells from patients with myelofibrosisbefore seeding in organoids (day 0) compared with cells isolated by flow cytometry 12 days after culture in organoids, indicating maintenance of clonal architecture. **K,** Workflow for organoid generation, engraftment with cells from patients with myelofibrosis, and treatment with inhibitors. **L,** αSMA and collagen 1 expression in nonengrafted organoids, and organoids engrafted with myelofibrosis cells treated with DMSO (control), SB431542, JQ1, and ruxolitinib. Each data point corresponds to total measurements per organoid within a block (*n* = 3 donors). One-way ANOVA with multiple comparisons (Fisher least significant difference). **M,** Representative images from **L**. Rux, ruxolitinib. *, *P* < 0.01; **, *P* < 0.05; ***, *P* < 0.001 for Mann–Whitney test. See also Supplementary Fig. S8.

Organoids were seeded with CD34^+^ HSPCs from healthy donors (*n* = 7) and patients with myelofibrosis (*n* = 10; Supplementary Table S3). Donor cells were labeled with the plekstrin homology domain dye CellVue Claret, and 5,000 donor cells were seeded into each well of a 96-well ULA plate containing individual organoids (Fig. 6A). CellVue labeling enabled the identification and tracking of donor cells within the organoid milieu. Confocal Z-stack imaging confirmed that labeled cells from healthy donors and patients with myelofibrosis efficiently engrafted and were distributed throughout the organoid architecture (Fig. 6B; Supplementary Fig. S8A).

After 14 days, engrafted organoids were assessed for fibrosis. Soluble TGFβ levels were significantly elevated in the culture media of organoids engrafted with myelofibrosis cells when compared with healthy donor–engrafted samples (Fig. 6C). Immunofluorescence imaging showed a significant increase in collagen 1 and αSMA in organoids engrafted with cells from patients with myelofibrosis at protein level (Fig. 6D and E; Supplementary Fig. S8B) as well as gene expression (Fig. 6F), with a concomitant decrease in expression of endothelial cell–associated genes *CDH5* and *TIE2* and vascularity (Fig. 6G–I).

Patient-derived CD34^+^ HSPCs cultured within the organoids underwent lineage differentiation. After 14 days, a quarter (22%) of the total cells in the organoids were positive for the fluorescent label indicating adult donor origin, and these cells had undergone myeloid differentiation with erythroid (CD34^−^ CD235^+^ CD71^+^), myelomonocytic (CD34^−^ CD45^+^ CD11^+^ CD14^+^), and megakaryocytic cell (CD34^−^ CD41^+^ CD42a^+^) immunophenotypes evident (Supplementary Fig. S9A). As expected, given the absence of lymphopoietic cytokines, no B or T cells were detected (Supplementary Fig. S9A and S9B). A small population of the label-positive cells retained CD34 expression even 14 days after seeding, and these cells showed lower rates of cell division, suggesting maintenance of a population of quiescent stem/progenitor cells in the organoids (Supplementary Fig. S9C) in addition to myeloid differentiation.

Crucially, the clonal architecture of the malignancies could be tracked following culture in the organoids. All mutations present in the HSPCs prior to seeding were detected in labeled cells sorted from the organoids 12 days after seeding, and at almost identical variant allele frequencies as the original sample (Fig. 6J).

### Patient Cell–Engrafted Organoids Allow for Testing of Potential Inhibitors of Fibrosis

We next assessed whether organoids engrafted with cells from patients with myelofibrosis enabled screening of potential inhibitors of fibrosis, to explore the utility of this platform for precision medicine approaches. Organoids seeded with cells from patients with myelofibrosis were cultured for 7 days and then treated with the TGFβ inhibitor SB431542, the BET inhibitor JQ1, or ruxolitinib (Fig. 6K). Only JQ1 treatment restored COL1A1 expression to the level seen in nonengrafted control organoids, and JQ1 also significantly reduced αSMA (Fig. 6L and M). Expression of COL1A1 was lower following ruxolitinib treatment, with only a minimal reduction in αSMA (Fig. 6L and M). Although SB431542 significantly inhibited the induction of COL1A1 and αSMA in organoids in response to TGFβ treatment (Fig. 5I), no significant reversal was observed on hallmarks of fibrosis induced by engraftment of patient cells, suggesting that additional profibrotic signals derive from the myelofibrosis clone beyond TGFβ or an inability to reverse fibrosis once established.

### Bone Marrow Organoids Support the Engraftment, Survival, and Proliferation of Primary Cells from a Range of Hematologic Malignancies

Finally, we investigated whether primary human cells of other blood cancer types would also successfully engraft the bone marrow organoids. We focused on hematologic malignancies that are particularly challenging to maintain *ex vivo* and/or model *in vivo*—multiple myeloma, acute lymphoblastic leukemia (ALL), and myeloid leukemias—explored whether the organoids could improve the survival of cells *ex vivo*, thereby enabling mechanistic studies and target screening for these cancer types.

**Figure 7.**
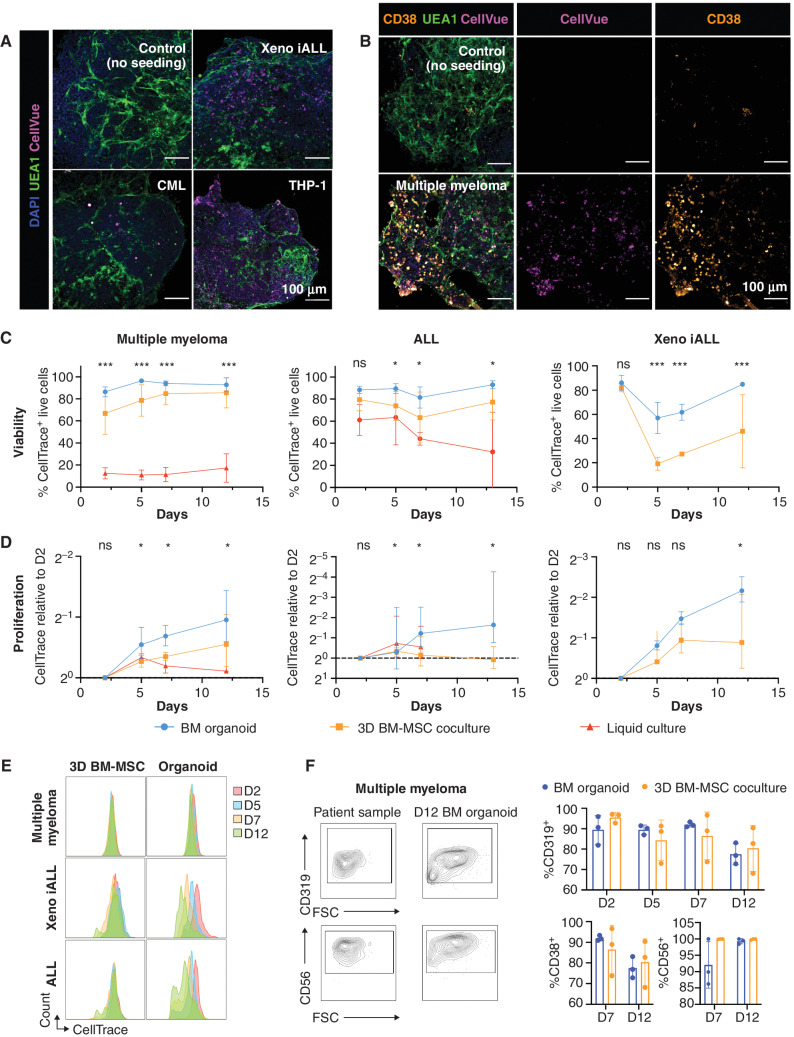
Bone marrow organoids support the engraftment, survival, and proliferation of cells from patients with myeloid and lymphoid hematologic malignancies. **A,** Organoids engrafted with CellVue-labeled model infant ALL cells from xenografts (Xeno iALL), primary cells from a patient with untreated CML, and THP-1 cells, an acute myeloid leukemia cell line. CellVue^+^ cells are visible throughout the volume of organoids. **B,** Organoids seeded with CD138^+^ cells isolated from bone marrow aspirates of patients with multiple myeloma show CellVue^+^ CD38^+^ plasma cell engraftment. **C–E,** Viability and proliferation of cells from 4 donors with multiple myeloma, 6 donors with ALL, and 3 Xeno iALL samples seeded simultaneously in the organoids, a 3D coculture with primary human BM-MSC (3D BM-MSC), and where possible, liquid culture. **E,** Serial dilution of CellTrace label, indicating cell proliferation, for multiple myeloma, Xeno iALL, and ALL cells in 3D BM-MSC and organoids on days 2, 5, 7, and 12 following thawing and plating. **F,** Engrafted multiple myeloma cells retained their immunophenotype at day 12 with more consistent maintenance of CD319 and CD38 in organoids than 3D BM-MSC. Representative images are shown. *, *P* < 0.01; ***, *P* < 0.001; ns, not significant. *n* = 4 for multiple myeloma, *n* = 3 for Xeno iALL, and *n* = 3 for ALL, with each repeat comprised of a separate donor two-way ANOVA with repeated measures and multiple comparisons (organoid cultures vs. 3D BM-MSC; Fisher least significant difference) for ALL and multiple myeloma and multiple unpaired *t* test for Xeno iALL data. BM, bone marrow; FSC, forward scatter.

To test this, cryopreserved cells were thawed and fluorescently labeled prior to seeding into 96-well plates containing organoids. Cells from patients with multiple myeloma (*n* = 5; Supplementary Table S4), ALL (*n* = 6, Supplementary Table S4), and chronic myeloid leukemia (CML; *n* = 2, Supplementary Table S3), a human AML cell line (THP-1), and leukemic cells from a human fetal liver–derived infant ALL xenograft model (Xeno iALL; *n* = 3, Supplementary Fig. S10) rapidly engrafted and were observed throughout the organoid volume (Fig. 7A and B). Distinct CellTrace-positive populations were detectable in the organoids for all 17 donor samples, confirming successful engraftment and survival of primary human and xenograft-derived cells over a 12-day time course (Supplementary Fig. S11A). Primary multiple myeloma cells were costained for CD38, confirming that cells derived from the malignant plasma cell clone had successfully engrafted (Fig. 7B).

We compared the survival and proliferation of primary multiple myeloma, ALL, and Xeno iALL cells engrafted in the organoids with cells seeded in wells with media alone or into a single-lineage 3D coculture system containing primary human bone marrow MSCs in a Matrigel + collagen I hydrogel (3D BM-MSC). Whereas multiple myeloma cells were less than 20% viable only 48 hours after seeding in wells with media alone, in stark contrast, the myeloma cells expanded and remained >90% viable more than 12 days after engraftment into organoids for all 5 donors tested (Fig. 7C and D). Similarly, the survival of primary ALL cells was significantly improved in the organoids compared with liquid culture (Fig. 7C). The cell viability for ALL and Xeno iALL was also higher in the organoids than in 3D BM-MSC cocultures (Fig. 7C), with higher proliferation rates (Fig. 7D and E). Plasma cells from patients with myeloma showed minimal cell division in either model (Fig. 7D), but the cells retained their original immunophenotype (CD38^+^, CD319^+^, and CD56^+^) more consistently in the organoids than in the 3D BM-MSC (Fig. 7F). Similarly, ALL and Xeno iALL cells showed improved maintenance of CD19 expression in the organoids as compared with 3D BM-MSC (Supplementary Fig. S12). Together, these data confirm that the organoids provide a supportive niche for the survival and growth of primary blood cancer cells from patients, including for cancer types that are otherwise poorly viable *ex vivo* and after cryopreservation.

## DISCUSSION

Here we describe the development of a protocol generating vascularized bone marrow organoids that faithfully model key cellular, molecular, and architectural features of myelopoietic bone marrow including stromal cells, lumen-forming vasculature, and myeloid cell types. We demonstrate the utility of these organoids for modeling cancer-induced perturbations to the bone marrow niche and myelofibrosis.

Treatment of organoids with TGFβ, the primary cytokine driving myelofibrosis, induced organoid fibrosis, enabling target prioritization and screening of potential inhibitors. Fibrosis also occurred following the engraftment of organoids with HSPCs from patients with myelofibrosis, but not healthy donors. The ability to reliably model bone marrow fibrosis is an important advance, as the lack of adequate *in vitro* and *in vivo* models currently hampers the efficient preclinical validation of strategies aiming to reduce or prevent fibrosis, which is a huge unmet need for patients with myeloproliferative neoplasms and other blood cancers (45). As the organoids are highly reproducible and feasible to generate at scale in 96- or 384-well plate formats, this system presents an ideal platform for high-throughput target screens using pharmacologic or genetic modulation.

We also show that cells from patients with both myeloid and lymphoid malignancies readily engraft and survive within the organoids, including cancer cell types that are notoriously difficult to maintain *ex vivo*. Remarkably, malignant cells from patients with multiple myeloma that had been cryopreserved and thawed prior to use were sustained by the organoids for 12 days, while rapidly losing viability when plated *in vitro* without stromal support. Maintenance of primary myeloma cells *ex vivo* and a method to study their interactions within a multicellular hematopoietic niche environment will enable preclinical pharmacogenomic screens and the study of disease mechanisms using primary cells from patients, which is currently a significant obstacle to translational research in this disease. Targeted genetic modification of organoids could be performed either by using lineage-specific promoters or by modulating the differentiation protocol to generate certain cellular subsets (e.g., stroma) from wild-type iPSCs, and then assembling these with hematopoietic cells generated from a genetically modified parent iPSC line.

A key limitation of current human *ex vivo* bone marrow models has been a lack of sinusoidal-like endothelium, with many systems reliant on human umbilical vein endothelial cells (HUVEC; ref. 13). We show here that the addition of VEGFC, recently shown to support the bone marrow perivascular niche (28), drives the generation of vasculature and supporting stroma that are specialized for hematopoietic support and phenocopy bone marrow sinusoidal endothelium. The resulting bone marrow organoids thereby present a unique opportunity to study the hematopoietic-niche cross-talk that underpins healthy hematopoiesis, and how perturbations to these regulatory interactions are permissive for the emergence and progression of cancers.

Although this system offers a substantial advance in the field, in its current iteration, no osteoid lineage, lymphoid cells, smooth muscle cells, or adipocytes are generated. Similarly, although we show a high degree of homology of the organoid vasculature to sinusoidal endothelium from human bone marrow, distinct arteriolar, and sinusoidal endothelial subtypes are not present in the organoids. The current differentiation protocol was optimized primarily to study myeloid malignancies and cancer-associated bone marrow fibrosis, and refinement of the growth factor supplements may improve the maintenance of lymphoid malignancies. In addition, the introduction of recirculating flow (46) may allow for the generation of organoids that mimic native bone marrow physiology more comprehensively. Despite these limitations, maintenance of cells from B-cell leukemias and plasma cell malignancies as well as myeloid cancers provides proof of principle that bone marrow organoids can be used to support a range of bone marrow cancers and cell types, paving the way for customization to support other relevant studies.

A protocol for the ectopic implantation of human bone marrow MSC-derived ossicles has previously been shown to support the engraftment of adult HSCs *in vivo* (47). Although the iPSC-derived organoids are not an *in vivo* system, they are substantially more efficient to generate than *in vivo* ossicles (weeks vs. months) and do not require human bone marrow or platelet lysates, which are hard to source and may induce experimental variability. The survival and continuous production of hematopoietic support factors in the absence of exogenous cytokine supplementation suggest that longer-term cultures may be possible. The implantation of organoids into mice has not yet been tested but may allow for longer-term studies in an *in vivo* setting.

The development of organoids has been transformative in other disease settings—for example, cerebral, lung, and kidney disease modeling (48). This platform may similarly be an enabling technology for the interrogation of disease mechanisms in hematologic cancers as well as the development and testing of novel therapies using human cells in a tissue-relevant system. Importantly, this platform is likely to reduce reliance on animal models. Target identification and screening using a species-specific, clinically relevant *ex vivo* model that can incorporate primary cells from patients may accelerate and increase the success rate of clinical translation.

## METHODS

### iPSC Culture and Differentiation

A Gibco Human Episomal iPSC (Thermo Fisher Scientific, cat. #A18945) line was maintained in StemFlex medium (Thermo Fisher Scientific, cat. #A3349401) and on Geltrex (Thermo Fisher Scientific, cat. #A1569601)-coated 6-well plates. The iPSC line was karyotyped prior to use (23), and potency markers were assessed upon expansion and freezing. A full, detailed description of passaging and differentiation protocols is included in Supplementary Materials and Methods. In brief, for differentiations, iPSCs were dissociated using EDTA when colonies were approximately 100 μm in diameter. The resulting iPSC aggregates were incubated overnight in StemFlex supplemented with RevitaCell in 6-well Co-star ULA plates (Corning, cat. #3471; day −1). After overnight incubation, cells were collected and resuspended in phase I medium (Supplementary Methods) and cultured at 5% O_2_ for 3 days (days 0–3). Aggregated cells were then collected again in phase II medium (Supplementary Methods). On day 5, cells were collected by gravitation for hydrogel embedding. Hydrogels were composed of 60% collagen (either type I, type IV, or an equal parts type I + IV mix) and 40% Matrigel as detailed in Supplementary Methods. Fully polymerized gels with cell aggregates were then supplemented with phase III media (Supplementary Methods). Media were replenished every 72 hours.

### Immunofluorescence Staining

Sections were blocked using 2% goat serum (Thermo Fisher Scientific, cat. #31872) and 1% bovine serum albumin (BSA; Sigma, cat. #A9418) prior to primary antibody labeling with antibody diluted in 1% BSA, sequential PBS washes, and secondary labeling with AlexaFluor conjugates. The whole organoid blocking solution was supplemented with Triton X100, Tween, and sodium deoxycholate as described by Wimmer and colleagues (21). Antibodies are listed in Supplementary Table S5, and additional details are given in Supplementary Methods.

### scRNA-seq, Data Processing, and Analysis

Cryopreserved cells pooled from 15 organoids from 3 differentiations from both VEGFA and VEGFA + C protocols were processed for single-cell sequencing as described in Supplementary Methods, and processed using the Chromium Single-Cell 3′ library and Gel Bead Kits v3.1 (10X Genomics) as per kit instructions. Demultiplexed FASTQ files were aligned to the human reference genome (GRCh38/hg38) using standard CellRanger (version 6.0.1) “cellranger count” pipeline (10X Genomics). SingCellaR (ref. 31; https://supatt-lab.github.io/SingCellaR.Doc/) was used for the downstream analysis.

### Flow Cytometry

Organoids were dissociated for flow cytometry analysis using collagenase type II (Sigma-Aldrich, cat. #C6885) resuspended in HEPES buffer (Sigma-Aldrich, cat. #H0887) at a concentration of 20 mg/mL. Samples were collected by gravitation in a 15-mL Falcon tube and washed 2× in PBS and then resuspended in collagenase type II. For dissociation, samples were incubated at 37°C for 5 minutes before trituration and a further 5-minute incubation. The dissociation reaction was stopped through the addition of PBS supplemented with FBS. Ten organoids were dissociated per flow cytometry experiment. Analysis was performed using either a cyan flow cytometer (Beckman Coulter) or an Attune NxT. Single color stained controls and fluorescence-minus-one controls were used for all experiments using antibodies as listed in Supplementary Table S5.

### TGFβ Treatment to Induce Organoid Fibrosis

Organoids were treated with TGFβ (PeproTech, cat. #100-21) at 10, 25, or 50 ng/mL for 72 hours. At 72 hours, all samples were approximately 90% viable after collagenase digestion. Samples were then collected for either whole-mount microscopy, paraffin/OCT embedding and sectioning, or qRT-PCR. Thirty-two organoids were treated per replicate; of these, 16 were spun down for RNA extraction for subsequent qRT-PCR. The remaining 16 organoids were fixed, with 8 taken for whole volume imaging and 8 per repeat taken for embedding and sectioning. For drug treatment, samples were treated with DMSO or TGFβ at a concentration of 25 ng/mL, and supplemented with an inhibitor as described (either JQ1 at 0.5 μmol/L or SB431542 at 20 μmol/L).

### qRT-PCR

Whole organoids were processed using either the Micro RNeasy Kit (Qiagen, cat. #74004) or Qiagen Mini RNA isolation kit (Qiagen, cat. #74104) as per kit instructions. cDNA was prepared using the High-Capacity cDNA Reverse Transcription Kit (Applied Biosystems, cat. #4368814) or EvoScript Universal cDNA Master (Roche, cat. #07912374001), and RT-PCR was performed using PowerUp SYBR Green Master Mix reagent (Applied Biosystems, cat. #A25742) or TaqMan Universal PCR Master Mix (Applied Biosystems; see Supplementary Table S6 for a list of primers).

### Seeding of Organoids and 3D BM-MSC Cultures with Hematopoietic Cells

Peripheral blood and bone marrow samples were collected from healthy donors and patients with hematologic malignancies following the provision of written informed consent in accordance with the Declaration of Helsinki. All participants donated human tissue for research without receiving monetary compensation, and the studies were approved by an institutional review board [myelofibrosis, CML, and G-CSF mobilized healthy apheresis donors—INForMed Study, University of Oxford (IRAS: 199833; REC 16/LO/1376); multiple myeloma—Oxford Radcliffe Biobank (Oxford Clinical Research Ethics Committee 09/H0606/5/5, project 16/A185); ALL samples: REC 16/LO/2055′ (IRAS 179685)]. Written informed consent was received from all the participants for the donation of human tissue.

For engraftments with CD34^+^ HSPCs, cryopreserved mononuclear cells were thawed and CD34^+^ viable cells were FACS isolated using a Becton Dickinson FACSAria Fusion Cell Sorter with 100-nm nozzles into 1.5 mL Eppendorf tubes prior to seeding in organoids. For inhibitor experiments, organoids were engrafted with CD34^+^ myelofibrosis cells and cultured for 7 days prior to the addition of inhibitors. Myeloma cells were selected from total bone marrow mononuclear cells using anti-CD138 magnetic bead enrichment (STEMCELL Technologies, cat. #17887) prior to cryopreservation. Xeno iALL cells were derived from a recently published xenograft model of infant ALL in which the t(4;11)/MLL–AF4 translocation was introduced into primary human fetal liver hematopoietic cells by CRISPR–Cas9 gene editing prior to transplantation into immunodeficient mice (49). All experiments were performed under a project license approved by the UK Home Office under the Animal (Scientific Procedures) Act 1986 after approval by the Oxford Clinical Medicine Animal Welfare and Ethical Review Body and in accordance with the principles of 3Rs (replacement, reduction, and refinement) in animal research. Cells were harvested from the bone marrow of leukemic mice at 17 to 18 weeks, and total bone marrow cells were cryopreserved. Following thawing and prior to seeding in the organoids, human CD45^+^ were selected using magnetic microbeads (Miltenyi Biotec, cat. #130-045-801). Over 90% of human CD45^+^ cells were CD19^+^ CD34^+^ lymphoblasts with a predominantly CD34^+^ pro-B phenotype (CD10^−^CD20^−^IgM/IgD^−^; Supplementary Fig. S10). Informed consent was provided by all participants for the donation of human tissue, and this study was approved by an institutional review board (REC: 18/NE/0290 and 18/LO/0822). The replicates included in this study were from 4 mice transplanted with MLL–AF4-edited cells from one human fetal liver sample. Details of donor cell labeling are included in Supplementary Materials and Methods. The composition of the engrafted organoids was analyzed by flow cytometry using either an LSRFortessa X50 (BD Biosciences) or an Attune NxT or a next-generation sequencing panel (see Supplementary Methods and Supplementary Table S7).

For the 3D BM-MSC cocultures, 24-well plates were prepared containing 300 μL of a 70:30 mix of collagen I:Matrigel per well and incubated at 37°C for 90 minutes. Primary bone marrow MSCs resuspended at 10,000 cells/mL in StemPro-34 supplemented medium were added to the wells (500 μL/well) and incubated overnight prior to seeding with primary hematopoietic cells.

### Data Analysis Software

scRNA-seq analyses were performed in R Studio (version 1.4.1106). Other statistical analyses were performed using GraphPad PRISM 7 with statistical tests as described in relevant figure legends. *P* values are defined as *, *P* < 0.01; **, *P* < 0.05; ***, *P* < 0.001; ****, *P* < 0.0001.

### Data Availability

scRNA-seq data are available at the Gene Expression Omnibus (accession GSE196684). Scripts used for analysis are available at https://github.com/aokhan/BMorganoidV1/ and https://github.com/supatt-lab/SingCellaR. Further data are available on request.

## Supplementary Material

Supplementary Information Supplementary Materials and Methods, Supplementary Figures S1-S12

Supp Table 1 Supplementary Table 1 VEGFAC Top Differentially Expressed Genes by Cluster

Supp Table 2 Supplementary Table 2 Gene sets for GSEA

Supp Table 3 Supplementary Table 3 HD and MPN samples.

Supp Table 4 MM ALL Donor Details

Supp Table 5 Supplementary Table 5 Antibodies

Supp Table 6 Supplementary Table 6 qRT PCR Probes

Supp Table 7 Supplementary Table 7 NGS Panel
