# Human bone marrow organoids for disease modelling, discovery and validation of therapeutic targets in hematological malignancies

**DOI:** 10.1158/2159-8290.CD-22-0199

**Published:** 2022-11-09

**Authors:** Abdullah O. Khan, Antonio Rodriguez-Romera, Jasmeet S. Reyat, Aude-Anais Olijnik, Michela Colombo, Guanlin Wang, Wei Xiong Wen, Nikolaos Sousos, Lauren C. Murphy, Beata Grygielska, Gina Perrella, Christopher B. Mahony, Rebecca E. Ling, Natalina E. Elliott, Christina Simoglou Karali, Andrew P. Stone, Samuel Kemble, Emily A. Cutler, Adele K. Fielding, Adam P. Croft, David Bassett, Gowsihan Poologasundarampillai, Anindita Roy, Sarah Gooding, Julie Rayes, Kellie R. Machlus, Bethan Psaila

**Affiliations:** 1 Institute of Cardiovascular Sciences, College of Medical and Dental Sciences, University of Birmingham, Vincent Drive, Birmingham, U.K, B15 2TT; 2 MRC Weatherall Institute of Molecular Medicine, Radcliffe Department of Medicine and National Institute of Health Research (NIHR) Oxford Biomedical Research Centre, University of Oxford, Oxford, U.K. OX3 9DS; 3 Centre for Computational Biology, MRC Weatherall Institute of Molecular Medicine, University of Oxford, Oxford, U.K. OX3 9DS; 4 Cancer and Haematology Centre, Churchill Hospital, Oxford University Hospitals NHS Foundation Trust, Oxford, U.K. OX3 7LE; 5 Rheumatology Research Group, Institute of Inflammation and Ageing, College of Medical and Dental Sciences, University of Birmingham, Vincent Drive, Birmingham, U.K. B15 2TT; 6 MRC Weatherall Institute of Molecular Medicine, Department of Paediatrics and National Institute of Health Research (NIHR) Oxford Biomedical Research Centre, University of Oxford, Oxford, U.K. OX3 9DS; 7 School of Dentistry, Institute of Clinical Sciences, University of Birmingham, Birmingham, U.K. B5 7EG; 8 Healthcare Technologies Institute, School of Chemical Engineering, University of Birmingham, Birmingham, B15 2TT, U.K; 9 University College London Cancer Institute, 72 Huntley Street, London WC1E 6DD; 10 Vascular Biology Program, Boston Children’s Hospital, Department of Surgery, Harvard Medical School, Boston, MA 02115, USA

**Keywords:** stroma, hematopoiesis, myelofibrosis, leukemia, megakaryocyte, microtissue, 3D culture, bone marrow fibrosis

## Abstract

A lack of models that recapitulate the complexity of human bone marrow has hampered mechanistic studies of normal and malignant hematopoiesis and the validation of novel therapies. Here, we describe a step-wise, directed-differentiation protocol in which organoids are generated from iPSCs committed to mesenchymal, endothelial and hematopoietic lineages. These 3-dimensional structures capture key features of human bone marrow – stroma, lumen-forming sinusoids and myeloid cells including pro-platelet forming megakaryocytes. The organoids supported the engraftment and survival of cells from patients with blood malignancies, including cancer types notoriously difficult to maintain *ex vivo*. Fibrosis of the organoid occurred following TGFβ stimulation and engraftment with myelofibrosis but not healthy donor-derived cells, validating this platform as a powerful tool for studies of malignant cells and their interactions within a human bone marrow-like milieu. This enabling technology is likely to accelerate discovery and prioritization of novel targets for bone marrow disorders and blood cancers.

## Introduction

The specialized bone marrow microenvironment maintains and regulates hematopoiesis, enabling adequate supply of blood cells to meet changing physiological requirements throughout life. Perturbations in the bone marrow hematopoietic niche contribute to the initiation and propagation of hematological malignancies. In addition, the stromal remodelling that occurs as a consequence of blood cancers contributes to bone marrow failure (1–4). Modelling bone marrow dysfunction is challenging, particularly in the context of human diseases. *In vitro* studies are often limited to two dimensional (2D) systems and simple co-cultures, in which the relevant cell types are absent, and many human diseases are inadequately reproduced by mouse models. Patient-derived xenografts have been used to model disease and validate targets *in vivo*, but some malignancies and hematological cell subtypes do not engraft well, even when humanised murine models are used (5–11).

Advances have been made in modelling certain marrow components on ‘biochips’ (12–16), but the lack of specialized stroma, vascularization and active blood cell generation remains a limitation with these methods. Co-culture with bone marrow mesenchymal stromal cells (MSCs) can support hematopoietic cell growth, either in 2D or in 3D with the addition of extracellular matrix support (17–19). While beneficial, these approaches are limited in terms of the elements of the bone marrow that are incorporated, as well as their scalability and reproducibility due to the limited availability and inter-donor variability of primary bone marrow MSCs. Improved *in vitro* systems are therefore required to enable more detailed mechanistic studies of human hematopoiesis, and to allow for the functional interrogation of the pathways and crosstalk that drive bone marrow malignancies.

The development and application of organoids – self-organizing, 3D, living multi-lineage structures – has the potential to facilitate translational research by enabling genetic screens and pharmacological modulation of disease pathobiology (20). Our goal was to generate a vascularised human bone marrow-like organoid that contains key hematopoietic niche elements and supports active endogenous hematopoiesis, as well as the growth and survival of hematopoietic cells from adult donors including malignant cell types that are difficult to grow and study *ex vivo*. Such a system would offer a scalable and highly manipulable human model for mechanistic studies and drug development and, importantly, may reduce dependence on animal models.

To achieve this, we optimised a protocol in which human induced pluripotent stem cells (iPSCs) generate mesenchymal elements, myeloid cells and ‘sinusoidal-like’ vasculature in a format that resembles the cellular, molecular and spatial architecture of myelopoietic bone marrow. We confirmed the homology of these organoids to human bone marrow using multi-modal imaging approaches and single cell RNA-sequencing (scRNAseq). Crucially, in addition to modelling physiological hematopoietic cell-niche interactions, we showed that the organoids supported the engraftment and survival of healthy and malignant hematopoietic cells from adult human donors, and enabled the screening of inhibitors of bone marrow fibrosis, a complication that occurs in patients with certain blood cancers and is associated with poor prognosis.

This platform addresses a long-standing need for 3D human bone marrow models for translational research where both niche and hematopoietic components are species and cell context specific, and creates a dynamic platform for high-throughput drug screening and studies aiming to understand disease pathways.

## Results

### Mixed-matrix hydrogels containing Matrigel and type I and IV collagens are optimal for the production of vascularised, myelopoietic organoids

**Figure 1:**
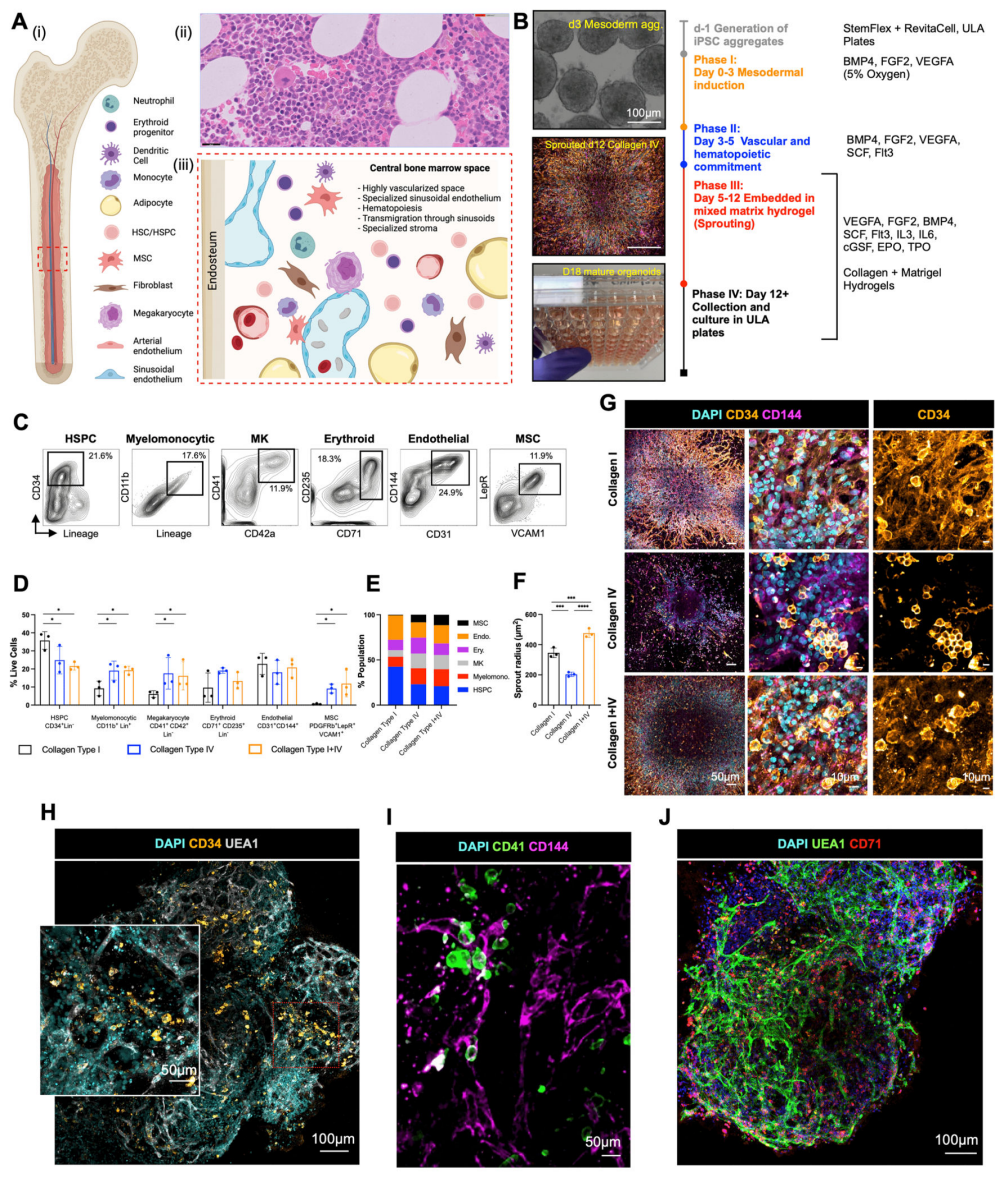
Mixed-matrix hydrogels containing Matrigel and type I and IV collagens are optimal for production of vascularised, myelopoietic organoids. **(A)** (i) Central bone marrow is a complex tissue including mesenchymal stromal cell (MSC), endothelial, hematopoietic stem and progenitor cells (HSC/HSPCs), myeloid and lymphoid subsets. (ii) Hematoxylin and Eosin stained section and (iii) model of human bone marrow highlighting the diverse hematopoietic and stromal cell types (created using Biorender.com). **(B)** Differentiation workflow, in which iPSC aggregates undergo mesodermal induction (day 0-3) and commitment to hematopoietic and vascular lineages (day 3 – 5). Cell aggregates are then embedded in mixed matrix hydrogels comprised of Matrigel and collagen I, collagen IV, or collagen I+IV mix at a 40:60 ratio to support vascular sprouting. Key media components are listed for each phase. **(C)** Gating strategy and **(D)** quantification of stromal and hematopoietic cell types in day 18 organoids supported by Matrigel + collagen type I only, collagen IV only and collagen I+IV hydrogels. **(E)** Distribution of lineages as fractions of the whole organoid population. **(F)** Radius of endothelial sprouts and **(G)** sprouting day 12 organoids immunostained for nuclei (DAPI), CD34 and CD144 (VE-cadherin). **(H, I, J)** Whole organoid Z-stack imaging acquired at day 18 showing **(H)** CD34^+^ HSPCs and UEA1+ vessels that are negative for CD34; **(I)** CD41^+^ megakaryocytes and **(J)** CD71^+^ erythroid cells dispersed throughout the organoids and associating with CD144^+^/UEA1^+^ vasculature. * p < 0.05, ** p < 0.01, *** p < 0.001, One-Way ANOVA with multiple comparisons (Fisher’s LSD) *n = 3* for endothelial sprout radius measurements. Two-Way ANOVA with multiple comparisons (Fisher’s LSD); *n = 3* (3 independent differentiations, 15 pooled organoids each) for flow cytometry analysis. Representative images shown. See also Figure S1.

To mimic the central bone marrow space (Fig. 1A), we devised a four-stage workflow to generate mesenchymal, vascular and myelopoietic marrow components. Human iPSCs were allowed to form non-adherent mesodermal aggregates (Phase I, days 0 – 3, Fig. 1B) before commitment to vascular and hematopoietic lineages (Phase II, days 3 – 5, Fig. 1B). The resulting cell aggregates were then embedded in mixed collagen-Matrigel hydrogels to induce vascular sprouting (Phase III, days 5 – 12, Fig. 1B). At day 12, sprouts were collected individually and cultured to form bone marrow organoids in 96-well ultra-low attachment (ULA) plates (Phase IV, days 12 onwards, Fig. 1B).

Hydrogels comprising Matrigel plus type I collagen have previously been used to support the formation of iPSC-derived blood vessel organoids (21). However, type IV collagen is more permissive than type I collagen for myeloid and megakaryocyte maturation in standard 2D *in vitro* culture systems(22,23). We therefore compared hydrogels containing type I and/or type IV collagen plus Matrigel for the generation of stromal, endothelial and myeloid lineages. Distinct immunophenotypic hematopoietic stem and progenitor cells (HSPC, CD34^+^ Lin^-^), myelomonocytic (CD34^-^ CD11b^+^ Lin^+^), megakaryocyte (CD34^-^ Lin^-^ CD41^+^ CD42b^+^), endothelial (CD31^+^ CD144^+^), erythroid (CD34^-^ Lin^-^ CD71^+^ CD235a^+^) and MSC populations (CD31^-^ CD140b^+^ VCAM1^+^ LepR^+^) were detected when organoids were digested at day 18 of differentiation (Fig. 1C – 1E). Organoids developed in collagen type I-only hydrogels contained the highest fraction of HSPCs but a low proportion of myelomonocytic cells, megakaryocytes and no MSCs, while collagen type IV and collagen types I+IV hydrogel-derived organoids yielded significantly larger myeloid and MSC populations, indicating that the addition of collagen IV created more favourable conditions for multilineage differentiation (Fig.1D & 1E, Suppl. Fig. 1A).

The hematopoietic niche contains a dense network of sinusoidal vessels, and specialized sinusoidal endothelium regulates stem cell self-renewal and differentiation, progenitor maturation and platelet generation (24,25). To assess vascularization of the organoids, we measured the degree of endothelial sprouting. Vascular sprout radii were significantly smaller in collagen type IV-only hydrogels compared to collagen types I-only and I+IV (average radius of 203.6 ± 14.61 μm *vs*. 345.9 ± 32.11 μm *vs*. 476.3 ± 28.82 μm respectively; Fig. 1F), and the density of CD34^+^ CD144 (VE-cadherin)-positive sprouts was significantly lower in collagen IV-only hydrogels (Fig. 1G). To assess vascularization and cellular architecture of the whole organoid in 3D, organoids were mounted in agarose and cleared with Ethyl Cinnamate for confocal Z-stack imaging. This revealed an elaborate network of UAE1^+^ CD144^+^ vessels throughout the collagen I+IV-derived organoids (Figs. 1H – 1J). In addition to CD34^+^ HSPCs (Fig. 1H), CD41^+^ megakaryocytes (Fig. 1I) and CD71 ^+^ erythroid cells (Fig. 1J) were distributed throughout the organoid volume and closely associated with the endothelium, as occurs in native bone marrow.

### Addition of VEGFC induced specialization of organoid vasculature to a bone marrow sinusoid-like phenotype

**Figure 2:**
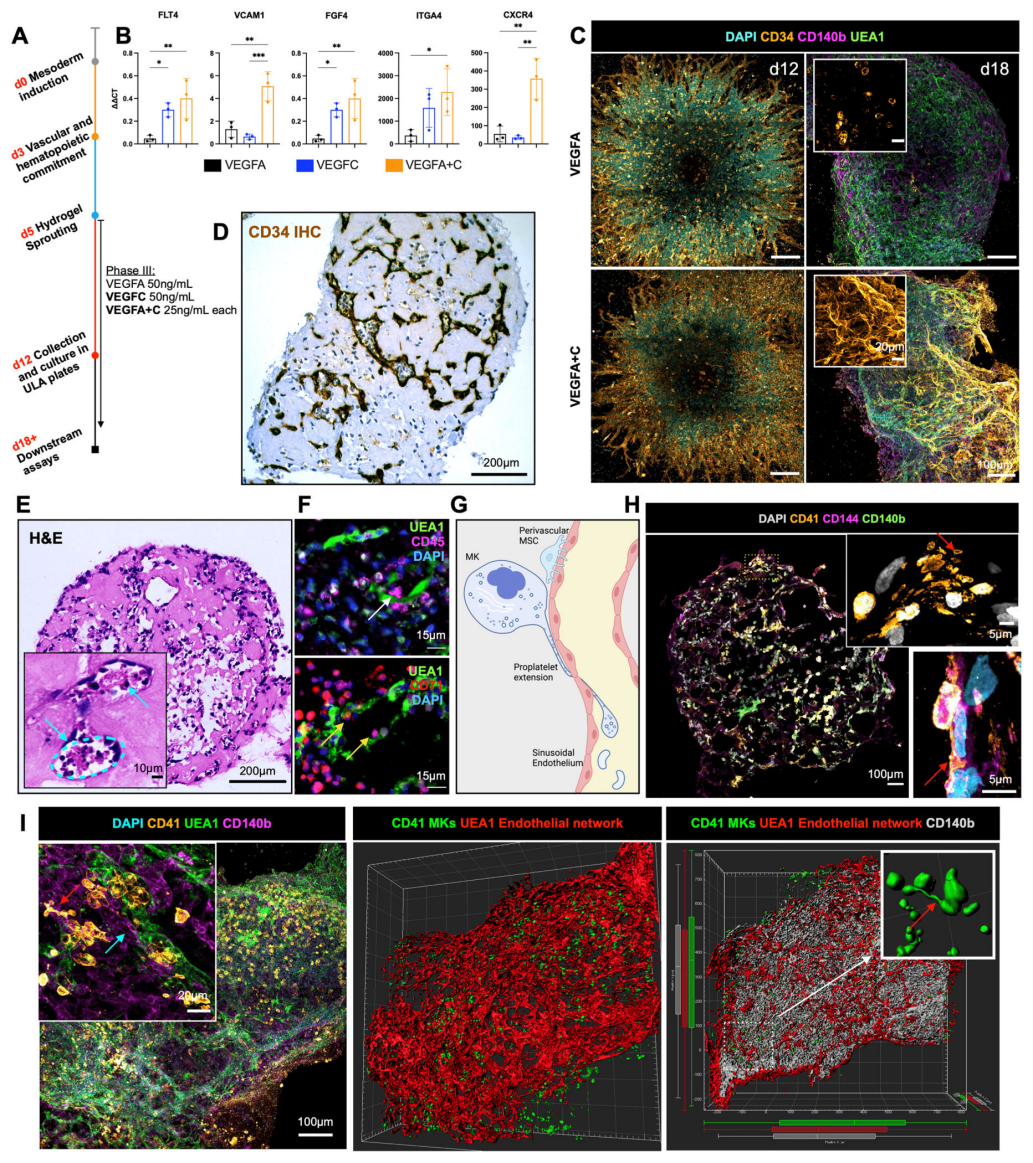
Addition of VEGFC induces specialization of organoid vasculature to a bone marrow sinusoid-like phenotype. **(A)** In the sprouting phase of differentiation (D5) in hydrogels, organoids were supplemented with either VEGFA or VEGFC, or both VEGFA and VEGFC. **(B)** mRNA expression of canonical cell surface receptors, growth factors and adhesion markers of bone marrow sinusoidal endothelium in VEGFA, VEGFC and VEGFA + C treated samples. ΔΔCt values relative to housekeeping (GAPDH) and undifferentiated iPSCs shown. Each data point represents 15 organoids, 3 independent differentiations shown. * p < 0.05, ** p < 0.01, *** p < 0.001, for one-way ANOVA with multiple comparisons (Fisher’s LSD). **(C)** CD34+ sprouting vessels at day 12 in both VEGFA and VEGFA+C conditions. At day 18, vessels were CD34 positive in VEGFA+C organoids but negative in VEGFA-only organoids. **(D)** Immunohistochemical staining for CD34 and **(E)** Hematoxylin and Eosin (H&E) staining of formalin-fixed, paraffin-embedded VEGFA+C organoid sections, with inset showing lumen-forming vessels containing hematopoietic cells (blue arrows). **(F)** Immunofluorescence staining of paraffin-embedded sections of VEGFA+C organoids showing CD45^+^ hematopoietic (white arrow) and CD71+ erythroid cells (yellow arrows) migrating into the UAE1+ vessel lumen. **(G)** Schematic demonstrating the process of proplatelet formation by megakaryocytes (image created by Biorender.com). **(H)** Whole organoid image showing CD140b+ MSCs surrounding CD144+ vessels, with CD41+ megakaryocytes. Insets show megakaryocytes extending pro-platelet protrusions into vessel lumen (red arrows). Top inset shows CD41^+^ plateletlike particles within vessel lumen. **(I)** Confocal imaging and 3D render of whole-mount VEGFA + C organoids showing CD41^+^ megakaryocytes (red arrow) closely associating with UEA1^+^ vessel network that is invested with CD140b^+^ fibroblast/MSCs (blue arrow) (left & centre image). Inset (right) shows 3D rendered megakaryocytes displaying proplatelet formation (red arrow).

Having determined that collagen I+IV Matrigel hydrogels enabled multil-ineage differentiation and the generation of a 3D vascular network, we sought to determine the optimal balance of endothelial growth factor support to generate organoid vasculature that resembles bone marrow sinusoids (24,25). Vascular endothelial growth factor (VEGF) A is a key regulator of blood vessel formation in health and disease, acting via the VEGF receptors VEGFR1 and 2 (26). Bone marrow sinusoidal endothelial cells express VEGFR1 and 2 as well as VEGFR3 (27), and VEGFC – the main ligand for VEGFR3 – was recently demonstrated to maintain the perivascular hematopoietic niche in murine bone marrow (28). We therefore tested the effect of adding VEGFC to the vascular sprouting phase (Day 5, Fig. 2A). The addition of VEGFC significantly increased the expression of *FLT4* (encoding VEGFR3) in the organoids, as well as HSPC adhesion molecules *(VCAM1, ITGA4)*, HSPC-supporting growth factors and chemotactic cytokines *(CXCR4, FGF4;* Fig. 2B). Supplementation with VEGFC in addition to VEGFA also induced retention of CD34 expression on organoid vessels, similar to native adult bone marrow vessels, while organoids stimulated with VEGFA alone expressed CD34 during vessel sprouting (days 5 – 12, Fig. 1G) but lost CD34 expression by day 18 (Fig. 1H & 2C).

Organoid vessels formed clear lumens, containing extravasating hematopoietic cells (Figs. 2D & 2E), confirmed by multiplexed immunostaining to identify CD45^+^ and CD71^+^ hematopoietic cells within the lumen of UEA1^+^ sinusoidal vessels (Fig. 2F). A hallmark of the bone marrow perivascular niche *in vivo* is the close association of megakaryocytes with sinusoidal endothelium, where they extend long, beaded proplatelet extensions into the vessel lumen (Fig. 2G). These extensions subsequently generate platelet buds under shear forces (Fig. 2G) (29). Cryosections of the bone marrow organoids stained for CD41^+^ MKs, CD144^+^ endothelium, and CD140b+ MSC/fibroblasts demonstrated classical proplatelet protrusions in association with the vessels (Fig. 2H & 2I), with remarkable similarity to previously published *in vivo* images of thrombopoiesis occurring in calvarial bone marrow (29). Volumetric imaging of whole-mount organoids demonstrated that these cells were organised in 3D in an extensive endothelial network invested with perivascular CD140b^+^ fibroblasts/MSCs. A higher number of megakaryocytes were observed in close proximity (5μm) to vessels in VEGFA+C-stimulated organoids than in VEGFA-only organoids (Suppl. Fig. 1B), consistent with the increased expression of chemotactic factors and adhesion proteins in VEGFA+C organoids (Fig. 2B). Together, these data indicate that the addition of VEGFC improved vascularization of the organoids and hematopoietic support.

### scRNAseq confirmed that hematopoietic and stromal cell lineages within organoids have transcriptional homology to human hematopoietic tissues

**Figure 3:**
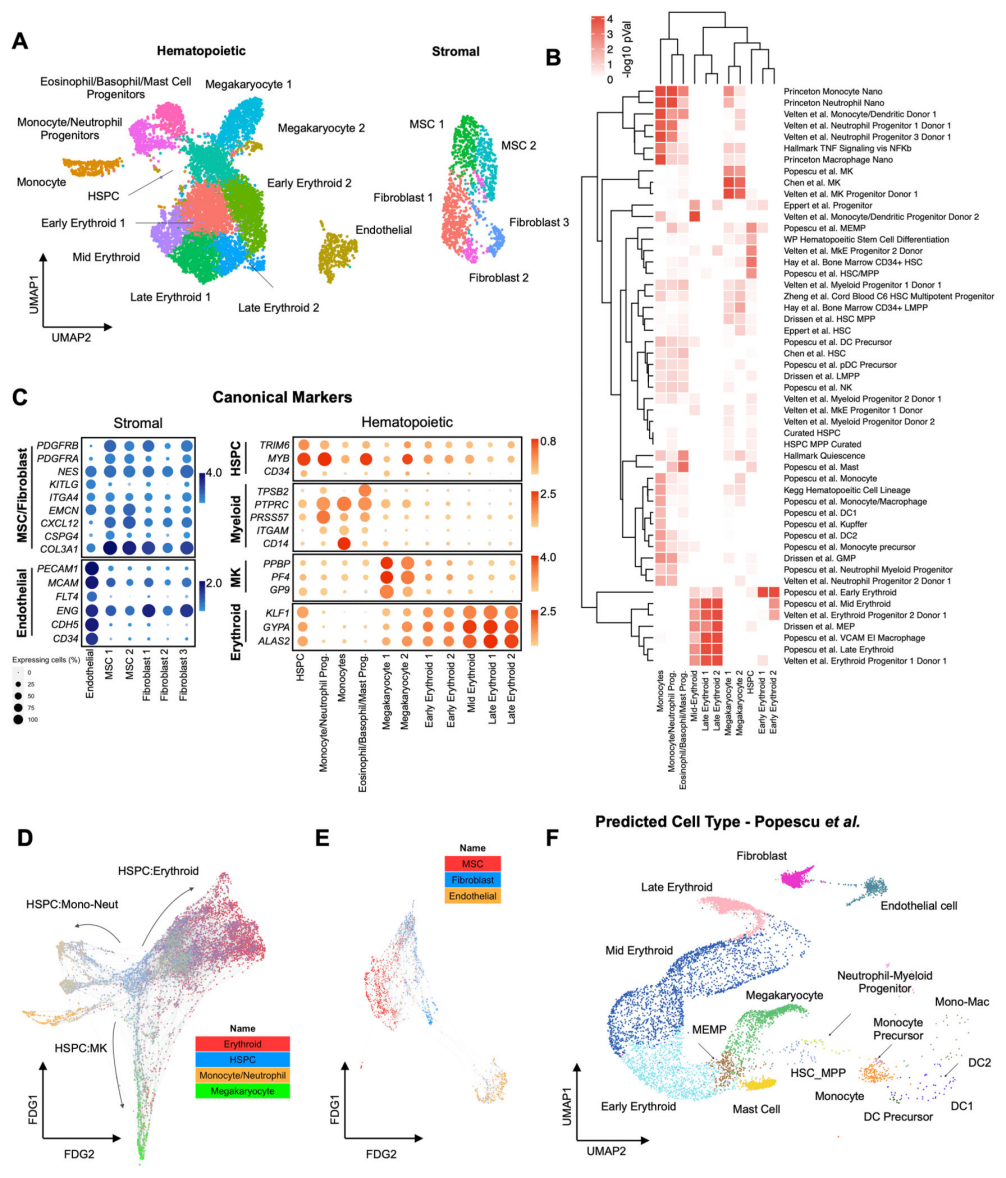
scRNAseq confirmed that hematopoietic and stromal cell lineages within organoids showed transcriptional similarity to human hematopoietic tissues. **(A)** Uniform Manifold Approximation and Projection (UMAP) plot showing annotated cell clusters. (**B**) Gene Set Enrichment Analysis (GSEA) of differentially expressed genes for each cluster using a curated set of 64 hematopoietic lineage gene sets. **(C)** Expression of canonical stromal and hematopoietic cell genes for each of the annotated clusters. Color scale represents the average level of expression and circle size shows % of cells within each cluster in which expression was detected for each gene. **(D & E)** Force-Directed Graph showing differentiation trajectories for **(D)** hematopoietic and **(E)** stromal compartments, superimposed with expression scores of lineage signature gene sets. **(F)** Organoid cells projected onto a published dataset of human hematopoietic and stromal cells using the Symphony package (33). See also Suppl. Figs 2 & 3 and Suppl. Tables 1 and 2.

To compare the cell types and molecular profiles of the organoids to human hematopoietic tissues, scRNAseq was performed on a total of 26,648 cells from 3 independent organoid differentiations using VEGFA only and VEGFA+C protocols. After quality control (see Methods), 19,506 cells (10,205 from VEGFA only and 9,301 from VEGFA+C) were included in downstream analyses. Distinct populations of key hematopoietic and stromal cell subtypes were identified including HSPCs, erythroid, neutrophil, monocyte, megakaryocyte, eosinophil/basophil/mast (EBM), fibroblasts, endothelial cells and MSCs (Fig. 3A, Suppl. Table 1), annotated using Gene Set Enrichment Analysis with a curated list of 64 published gene sets (Fig. 3B, Suppl. Table 2) (30,31) as well as the expression of canonical marker genes (Fig. 3C). Erythroid, megakaryocytic, monocytic/neutrophil and EBM populations demonstrated expression of *GYPA* and *KLF1, PF4* and *PPBP, CD14* and *RUNX1*, and *TPSB2* and *KIT* respectively (Fig. 3C).

Within stromal cell subsets, expression of *COL3A1*, platelet-derived growth factors *(PDGFRA/B)* and the key HSPC niche factor *CXCL12* (SDF-1) was observed in fibroblasts and MSCs, with particularly high expression of *CXCL12* in the MSCs, suggesting a key role for MSCs in homing and maintenance of HSPCs to perivascular regions of the organoids (Fig. 3C, Suppl. Figs. 2A & B). High expression of *PECAM1, CDH5* and *ENG* was detected in endothelial cells, confirming vascular specification (Fig. 3C, Suppl. Figs. 2A & 2B). The relative frequency of cell types in VEGFA-only organoids was broadly similar to that of VEGFA+C organoids, with a relative increase in abundance of endothelial and erythroid cells captured from organoids grown in the VEGFA+C condition (Suppl. Fig. 2C).

Trajectory analysis using a force-directed graph (FDG) showed that the organoids contained cells recapitulating the three main routes of hematopoietic myeloid differentiation (Fig. 3D, Suppl. Fig. 2D), similar to observations in human bone marrow (31). FDG analysis of the stromal cell populations showed independent routes of differentiation for endothelium and MSC/fibroblasts, as expected (Fig. 3E, Suppl. Fig. 2E).

To determine the transcriptional similarity of the bone marrow organoid to native human hematopoietic tissues, organoid scRNA-seq data was projected onto published single cell datasets of cells isolated from adult human bone marrow (31,32), fetal liver and fetal bone marrow (33) using the Symphony package (34). This revealed extensive overlap of organoid-derived cells with HSPCs, myeloid subsets, fibroblast/MSC and endothelial cell types, with the predicted cell types matching the cluster annotations (Fig. 3F, Suppl. Fig. 3A-C).

**Figure 4:**
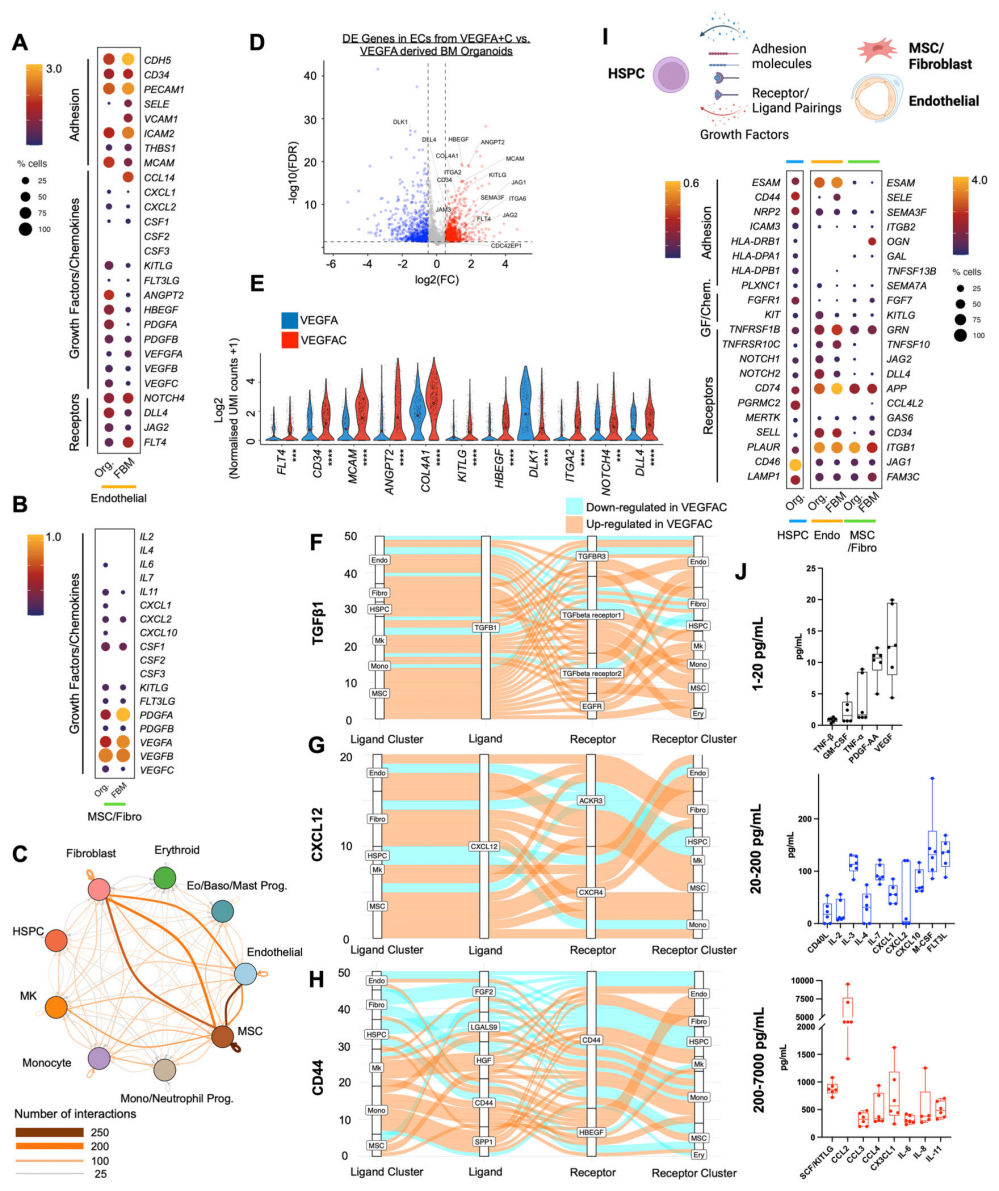
Endothelial, fibroblasts and mesenchymal stromal cells from organoid stroma support hematopoiesis, with increased hematopoietic support from VEGFA+C stimulated vasculature. **(A)** Comparison of expression of key receptors, adhesion proteins, growth factors and chemokines in endothelial cells from bone marrow organoids (Org) and human fetal bone marrow (FBM). **(B)** Comparison of expression of growth factors and chemokines in mesenchymal stromal cells (MSCs) from organoids and FBM. Size of dots represents % of expressing cells and color density indicates level of expression. **(C)** Total predicted ligand receptor interactions across clusters as predicted by CellPhoneDB (V2.0) showing extensive autocrine and paracrine interactions across BM organoid. **(D)** Volcano plot showing significantly differentially expressed (DE) genes in endothelial cells (ECs) from VEGFA+C vs. VEGFA-only organoids (801 significantly up- and 700 significantly down-regulated genes, p < 0.05, log2FC > 0.5 or -0.5). **(E)** Violin plots showing key hematopoietic support factors and markers of bone marrow sinusoidal endothelium in ECs of VEGFA+C and VEGFA-only organoids. *P* values indicated below x-axis labels and mean value is indicated on violin plots. *** p < 0.001, **** p < 0.0001 for pairwise comparison Wilcox test applied [FDR]). **(F, G, H)** Sankey plots comparing **(F)** *TGFβ1;* **(G)** *CXCL12* and **(H)** *CD44*-mediated interactions in VEGFA+C *vs*. VEGFA stimulated organoids. **(I)** Expression of interacting receptorligand pairs between organoid hematopoietic stem/progenitor cells (HSPCs) and cognate partner in organoid or FBM endothelial cells (endo) and MSC/fibroblasts, with percent of expressing cells and level of expression shown. **(J)** Hematopoietic cytokines/growth factors produced by bone marrow organoids, measured by Luminex assay. Each data point represents supernatant pooled from 16 separately generated organoids. See also Suppl. Figs. 4, 5, 6 & 7.

To explore the transcriptional similarity between the stromal support elements of the organoids with that of native human bone marrow in more detail, we extracted the stromal cell populations from a recently published study of fetal bone marrow that provided the first comprehensive annotation of stromal cell subsets in human bone marrow (35) including endothelial, MSC, fibroblast, and osteochondral lineage subsets. Integration and unsupervised clustering showed close approximation of organoid MSCs/fibroblasts (n=687) and endothelial cells (n=766) with the relevant cell clusters extracted from the human bone marrow dataset (endothelial, n=766 and MSC/fibroblasts, n=687 cells; Suppl. Figs. 4A – 4C). As expected, osteolineage, chondrocytes, smooth muscle and Schwann cells present in fetal bone marrow were absent from the organoids, and the distinct populations of sinusoidal and non-sinusoidal ‘tip’ endothelial cells were also not detected. However, expression of adhesion proteins, cytokines and hematopoietic support factors were very similar between organoid and bone marrow cells, including *CD34, PECAM, KITLG, FLT3LG, ANGPT2* for endothelial cells (Fig. 4A) and *KITLG, PDGF* and *VEGF* family members for MSCs/fibroblasts (Fig. 4B), confirming that the iPSC bone marrow organoid-derived stromal cells are highly homologous to their native bone marrow counterparts.

### Bone marrow organoids recapitulate cellular and molecular cross-talk between hematopoietic, endothelial and stromal cells

To investigate the cellular and molecular interactions between hematopoietic, endothelial and stromal cell subtypes within the organoids, we mapped the expression of interacting receptor and ligand pairs across clusters. Complex communication networks were predicted, both within and between hematopoietic and stromal cell compartments (Fig. 4C). Strong autocrine and paracrine interactions were predicted between MSCs, fibroblasts, endothelial cells, and monocytes, HSPCs and megakaryocytes, while erythroid cells showed weak interactions (Fig. 4C, Suppl. Figs. 5A – C).

Numerous interacting receptor-ligand partners were detected between megakaryocytes and endothelial cells (Suppl. Fig. 5B, Suppl. Fig 6A), and megakaryocytes with MSCs (Suppl. Fig. 6B), indicating bi-directional regulatory interactions between these cell types. These included *NOTCH1-JAG1/2, FLT4-PDGFC, ANGPT2-TEK* and *FLT1-VEGFB* for endothelium:megakaryocytes (Suppl. Fig. 5B & Suppl. Fig. 6A), and *NOTCH1-DLL4, KIT-KITLG, FGF2-CD44, SELP-CD34* and *VEGFRA-FLT1* for megakaryocytes:endothelial cells (Suppl. Fig. 6A). Interactions between megakaryocytes and MSCs were dominated by growth factors produced by megakaryocytes, including transforming growth factor beta (TGFβ), platelet-derived growth factor *(PDGF)*, fibroblast growth factor (*FGF*) and *VEGF* family members and their cognate receptors (Suppl. Fig. 6B).

Similarly, monocytes and endothelial cells demonstrated abundant interacting partners indicative of regulatory interactions, including *TNF-NOTCH1, JAG1/2-NOTCH, SIRPA-CD47*, and *LGALS9, ICAM, VEGF* family members with cognate receptors (Suppl. Fig. 5C and Suppl. Fig. 6C).

Significantly interacting partners between monocytes and MSCs/fibroblasts included *CXCL12-CXCR4, ICAM1-aXb2, SPP1-CD44*, interleukins 1 and 16, hepatocyte and fibroblast growth factors with their respective binding partners (Suppl. Fig. 6D).

Although a high number of regulatory interactions between hematopoietic and stromal compartments were detected, interactions between the stromal cell subsets (endothelial cells:MSCs:fibroblasts) were particularly strong (Fig. 4C). Significantly interacting partners identified included key regulatory molecules such as *JAG-NOTCH, VEGF, DLL4-NOTCH3, PDGF-PDGFR, ANPT1/2-TEK, IL33-IL33R, FGF* and *TGFB*(Suppl. Fig. 6E) (36–38).

To explore the impact of the addition of VEGFC to the differentiation protocol on the phenotype of organoid vasculature, we compared the transcriptomes of endothelial cells captured from VEGFA and VEGFA+C derived bone marrow organoids by scRNAseq. 1501 genes were significantly differentially expressed between endothelial cells generated with VEGFA only *vs*. VEGFA+C, including 801 up-regulated and 700 down-regulated genes (*p*< 0.05, log2FC > 0.5 or -0.5) (Fig. 4D). Canonical markers of bone marrow sinusoidal endothelium were more highly expressed in endothelial cells from VEGFA+C organoids than VEGFA only organoids, including *FLT4* (VEGFR4)*, CD34, MCAM, ANGPT2, COL4A1, COL4A2, ITGA2, CDC42EP1* and the notch ligand *DLL4* (25,35), while *DLK1* – a negative regulator of hematopoiesis (39) – was significantly lower in VEGFA+C-stimulated organoids than VEGFA-only (Figs. 4D & 4E).

In addition to improved hematopoietic support from endothelial cells, key regulatory axes were also upregulated across other cellular subsets in VEGFA+C organoids compared to VEGFA organoids (Fig. 4F – H). TGFβ1 signalling primarily from MSCs, megakaryocytes and fibroblasts was increased overall in VEGFA+C organoids, across the different TGFβ receptors (Fig. 4F). Similarly, CXCL12 signalling via CXCR4 and ACKR3 receptors across both stromal and haematopoietic cell types was also elevated (Fig. 4G), while the impact of VEGFC on signalling between CD44 and its binding partners was more mixed (Fig. 4H).

The receptor-ligand communication networks predicted between hematopoietic cells and niche components in the organoid stroma mirrored many of the communication networks that have been reported in native bone marrow (35), including *CD44-SELE, CD74-APP, KIT-KITLG, ICAM3-ITGB2, CD46-JAG1, and NOTCH2-DLL4* (Fig. 4I).

Finally, we explored whether the expression of hematopoietic support factors detected in the bone marrow organoid niche cells by scRNAseq (Suppl. Fig. 7A) could be confirmed at protein level. Organoids generated with VEGFA+C were harvested at day 18, and washed and re-plated in media supplemented with L-Glutamine but no added cytokines or growth factors, and cultured for a further 12 days without any added supplements with 50:50 media changes every 72 hours. In the absence of exogenously supplied cytokines, the organoids secreted multiple hematopoietic factors including SCF/KITLG, CCL2-4, interleukins (2, 3, 4, 6, 7, 8, 11), PDGF, FLT3L, M-CSF, GM-CSF, and VEGF (Fig. 4J, Suppl. Fig. 7B), confirming that the organoid stroma expresses key growth factors that might endogenously support hematopoiesis.

### Bone marrow organoids model the TGFβ-induced bone marrow fibrosis that occurs in hematological cancers and provide an *ex vivo* platform for inhibitor screening

**Figure 5:**
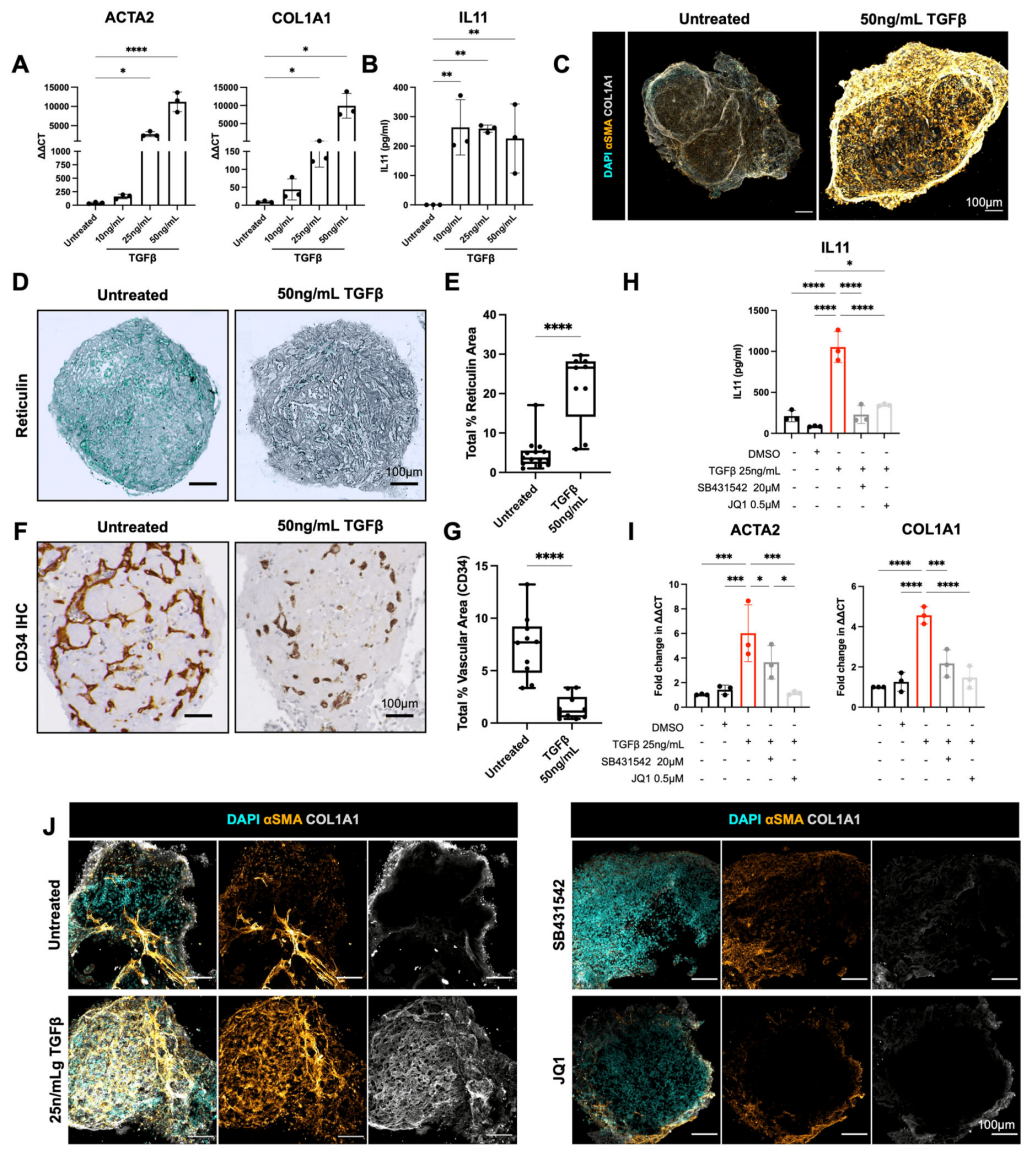
Bone marrow organoids model TGFβ-induced bone marrow fibrosis and enable inhibitor screening. **(A)** Organoids were treated with 10, 25, or 50ng/mL recombinant TGFβ and evaluated by qRT-PCR for expression of *ACTA2* (αSMA) and *COL1A1*, indicators of fibrosis. **(B)** Soluble IL11 detected in organoid media following treatment of organoids with TGFβ. **(C)** Confocal Z-stack images of whole, untreated and TGFβ (50ng/mL)-treated organoids stained for αSMA and COL1A1. **(D)** Reticulin staining of formalin-fixed, paraffin-embedded sections of TGFβ-treated organoids *vs*. control. **(E)** Measurement of total reticulin stained area in untreated and TGFβ (50ng/mL)-treated organoids. **(F)** CD34 immunostaining of organoid vessels and **(G)** quantification of total vascular area of organoids with/without TGFβ-treatment. **(H)** Effect of two potential inhibitors of TGFβ-induced fibrosis (SB431542 and JQ1) on IL11 secretion and **(I)** *ACTA2* and *COL1A1* expression. **(J)** αSMA and COL1A1 expression in TGFβ-treated organoids with/without indicated inhibitors. Representative images shown. * p < 0.01, ** p < 0.05, *** p < 0.001, **** p < 0.0001 for One-Way ANOVA with multiple comparisons (Fisher’s LSD). T-tests performed for image analysis of paraffin embedded sections (reticulin and CD34). *N* = 3 with each repeat comprising of 15 organoids pooled from 3 independent differentiations and treatments,

Pathological hematopoietic niche remodelling occurs in the majority of hematological malignancies. In certain cancers, particularly myeloproliferative neoplasms, myelodysplasia, acute leukemia and mast cell neoplasms, bone marrow fibrosis is a major cause of bone marrow failure and morbidity, and is associated with a poor prognosis (40). Fibrosis results from the excess production and release of pro-fibrotic cytokines by hematopoietic cells – in particular TGFβ – leading to the deposition of reticulin and collagen fibres by marrow stroma(40–42). To investigate whether the organoids could model pathological bone marrow fibrosis, we treated organoids with varying doses of TGFβ (10ng/mL, 25ng/mL, 50ng/mL), which resulted in a dose-dependent increase in the expression of hallmarks of fibrosis, including alpha smooth muscle actin (αSMA *[ACTA2])* and collagen 1 *(COL1A1)* (Fig. 5A), both canonical markers of fibroblast activation (42). A significant increase in soluble IL11 was also observed (43)(Fig. 5B). Collagen deposition within the organoids was markedly increased following TGFβ treatment (Fig. 5C), with pronounced reticulin fibrosis (Fig. 5D & Fig. 5E), recapitulating changes seen in the bone marrow of patients with myelofibrosis. The induction of organoid fibrosis was accompanied by reduced vascularization, suggesting multi-lineage remodelling as a consequence of TGFβ stimulation, as occurs in adult bone marrow (Fig. 5F, G).

We then explored the utility of this system to test potential inhibitors of fibrosis, selecting two compounds that inhibit pathways currently under investigation in clinical trials for myeloid malignancies - SB431542, an inhibitor of the TGFβ superfamily type I activin receptors, and the BET bromodomain inhibitor JQ1 (44). TGFβ-induced expression of soluble IL11 was completely inhibited by both treatments (Fig. 5H), while *ACTA2* and *COL1A1* over-expression was normalized by JQ1 and reduced by 1.5-fold and 2.5-fold respectively by SB431542 at the transcript level (Fig. 5I), and this reduction was also evident at protein level by immunofluorescence imaging (Fig. 5J). Together, these data confirm that the bone marrow organoids provide an efficient model of malignant bone marrow fibrosis and enable screening for the efficacy of potential pharmacological modulators.

### Organoid ‘niche remodelling’ and fibrosis occurred following engraftment with cells from patients with myelofibrosis but not healthy donors

Having confirmed substantial homology to native bone marrow, we hypothesized that the organoids may support the engraftment of primary cells from patients with hematological malignancies, enabling the modelling of cancer-stroma interactions and the possibility of patient-specific cytotoxic screens. Given the fibrosis observed following treatment with TGFβ, and the current lack of adequate *in vitro* and *in vivo* systems for modelling cancer-induced bone marrow fibrosis, we first seeded the organoids with cells from healthy donors and patients with myelofibrosis, and tested the impact of engraftment on the remodelling of the bone marrow organoid “niche”.

**Figure 6:**
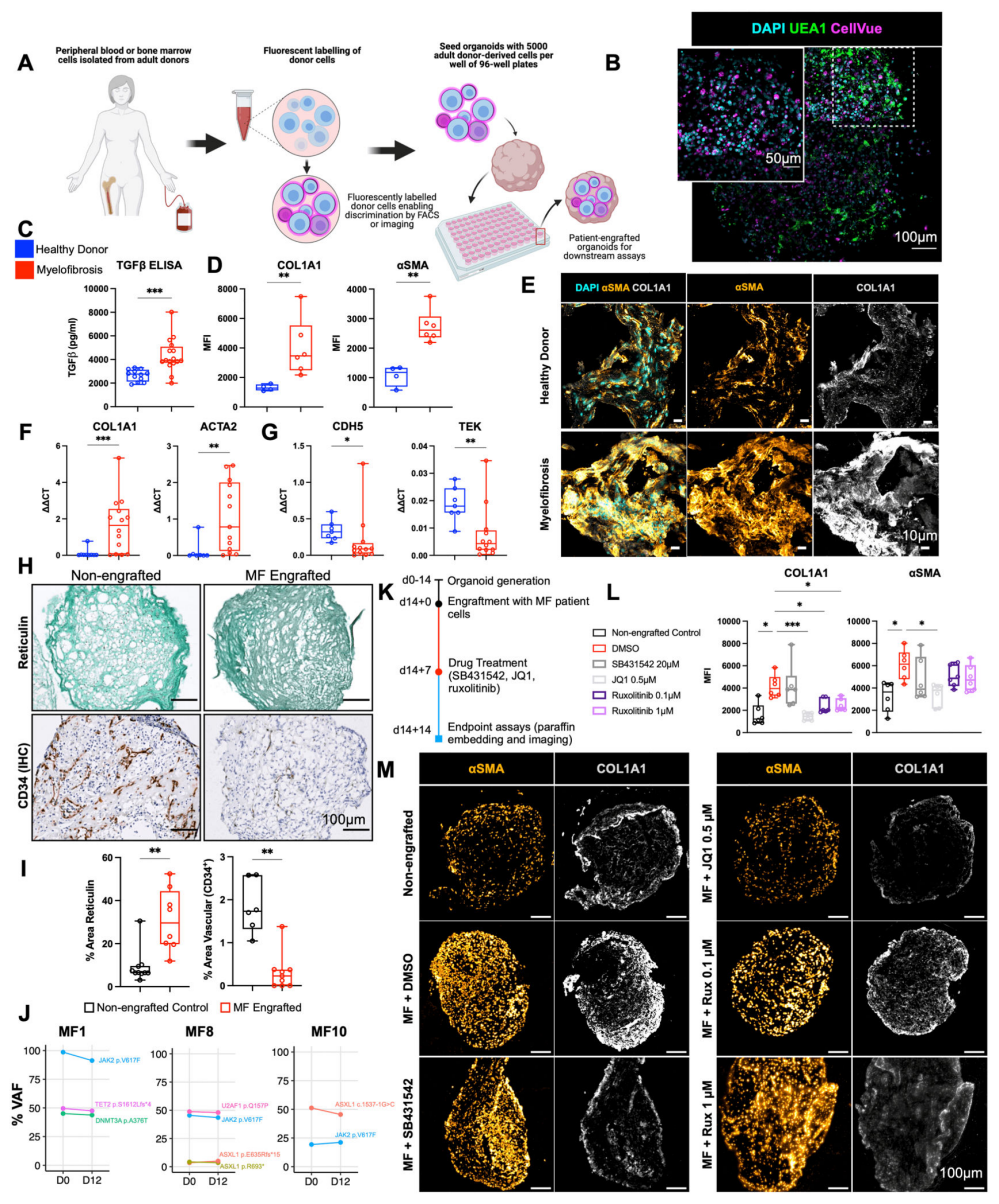
Engraftment of cells from patients with myelofibrosis, but not healthy donors, results in organoid ‘niche remodelling’ and fibrosis. **(A)** Cryopreserved peripheral blood or bone marrow cells from healthy donors and patients with blood cancers were fluorescently labelled and 5000 donor cells seeded into each well of a 96-well plate containing individual organoids. **(B)** Maximum-intensity projection of confocal Z-stack of a whole engrafted organoid 72 hours after seeding of the wells with donor cells, indicating donor cells engrafted throughout the volume of the organoids. **(C)** Soluble TGFβ in organoids engrafted by cells from myelofibrosis patients and controls. **(D, E, F,G)** Comparison of organoids engrafted with healthy donor and myelofibrosis cells for **(D)** collagen 1 (COL1A1) and αSMA immunofluorescence and **(E)** representative images; **(F)** *Col1A1* and *ACTA2* gene expression; **(G)** *CDH5* and *TIE2* expression. (* p < 0.01, ** p < 0.05, *** p < 0.001 for Mann-Whitney test, *n =* 4 healthy donors; *n = 7* MF samples for qRT PCR, *n =* 4 healthy donors; *n = 6* MF samples for imaging and quantification of cryo-sections). **(H, I)** Increased reticulin deposition with concomitant reduction in vascular area in organoids engrafted with myelofibrosis cells vs. non-engrafted control organoids with paired T-tests, each data corresponds to a single organoid engrafted with cells from 3 donors. **(J)** Variant allele frequencies of mutations detected by NGS of cells from myelofibrosis patients before seeding in organoids (day 0) compared to cells isolated by flow cytometry 12 days after culture in organoids, indicating maintenance of clonal architecture. **(K)** Workflow for organoid generation, engraftment with cells from myelofibrosis (MF) patients and treatment with inhibitors. **(L)** αSMA and collagen 1 expression in non-engrafted organoids, and organoids engrafted with myelofibrosis cells treated with DMSO (control), SB431542, JQ1, and Ruxolitinib. Each data point corresponds to total measurements per organoid within a block (n=3 donors). One-Way ANOVA with multiple comparisons (Fisher’s LSD). (**M)** Representative images from (L). Schematic in (A) created on Biorender.com. * p < 0.01, ** p < 0.05, *** p < 0.001. See also Suppl. Fig. 8.

Organoids were seeded with CD34^+^ HSPCs from healthy donors (n=7) and patients with myelofibrosis (n=10, Suppl. Table 3). Donor cells were labelled with the plekstrin homology domain dye CellVue Claret, and 5000 donor cells seeded into each well of a 96-well ultra-low attachment plate containing individual organoids (Fig. 6A). CellVue labelling enabled the identification and tracking of donor cells within the organoid milieu. Confocal Z-stack imaging confirmed that labelled cells from healthy donors and patients with myelofibrosis efficiently engrafted and were distributed throughout the organoid architecture (Fig. 6B, Suppl. Fig. 8A).

After 14 days, engrafted organoids were assessed for fibrosis. Soluble TGFβ levels were significantly elevated in the culture media of organoids engrafted with myelofibrosis cells when compared to healthy donor-engrafted samples (Fig. 6C). Immunofluorescence imaging showed a significant increase in collagen 1 and αSMA in organoids engrafted with cells from patients with myelofibrosis at protein level (Fig. 6D & 6E, Suppl. Fig. 8B) as well as gene expression (Fig. 6F), with a concomitant decrease in expression of endothelial cell-associated genes *CDH5* and *TIE2* and vascularity (Fig. 6G – I).

Patient-derived CD34^+^ HSPCs cultured within the organoids underwent lineage differentiation. After 14 days, around a quarter (22%) of the total cells in the organoids were positive for the fluorescent label indicating adult donor origin, and these cells had undergone myeloid differentiation with erythroid (CD34^-^ CD235^+^ CD71^+^), myelomonocytic (CD34^-^ CD45^+^ CD11^+^ CD14^+^), and megakaryocytic cells (CD34^-^ CD41^+^ CD42a^+^) immunophenotypes evident (Suppl. Fig 9A). As expected given the absence of lymphopoietic cytokines, no B or T cells were detected (Suppl. Fig. 9A & 9B). A small population of the label-positive cells retained CD34 expression even 14 days after seeding, and these cells showed lower rates of cell division, suggesting maintenance of a population of quiescent stem/progenitor cells in the organoids (Suppl. Fig. 9C), in addition to myeloid differentiation.

Crucially, the clonal architecture of the malignancies could be tracked following culture in the organoids. All mutations present in the HSPCs prior to seeding were detected in labelled cells sorted from the organoids 12 days after seeding, and at almost identical variant allele frequencies as the original sample (Fig. 6J).

### Patient cell-engrafted organoids allow for testing of potential inhibitors of fibrosis

We next assessed whether organoids engrafted with cells from patients with myelofibrosis enabled screening of potential inhibitors of fibrosis, to explore the utility of this platform for precision medicine approaches. Organoids seeded with cells from patients with myelofibrosis were cultured for 7 days, then treated with the TGFβ inhibitor SB431542, BET inhibitor JQ1 or ruxolitinib (Fig. 6K). Only JQ1 treatment restored COL1A1 expression to the level seen in non-engrafted control organoids, and JQ1 also significantly reduced αSMA (Fig. 6L & 6M). Expression of COL1A1 was lower following ruxolitinib treatment, with only a minimal reduction in αSMA (Fig. 6L & 6M). Although SB431524 significantly inhibited the induction of COL1A1 and αSMA in organoids in response to TGFβ treatment (Fig. 5I), no significant reversal was observed on hallmarks of fibrosis induced by engraftment of patient cells, suggesting that additional pro-fibrotic signals derive from the myelofibrosis clone beyond TGFβ or an inability to reverse fibrosis once established.

### Bone marrow organoids support the engraftment, survival and proliferation of primary cells from a range of hematological malignancies

Finally, we investigated whether primary human cells of other blood cancer types would also successfully engraft the bone marrow organoids. We focused on hematological malignancies that are particularly challenging to maintain *ex vivo* and/or model *in vivo* – multiple myeloma, acute lymphoblastic leukemia (ALL) and myeloid leukemias, and explored whether the organoids could improve the survival of cells *ex vivo*, thereby enabling mechanistic studies and target screening for these cancer types.

**Figure 7:**
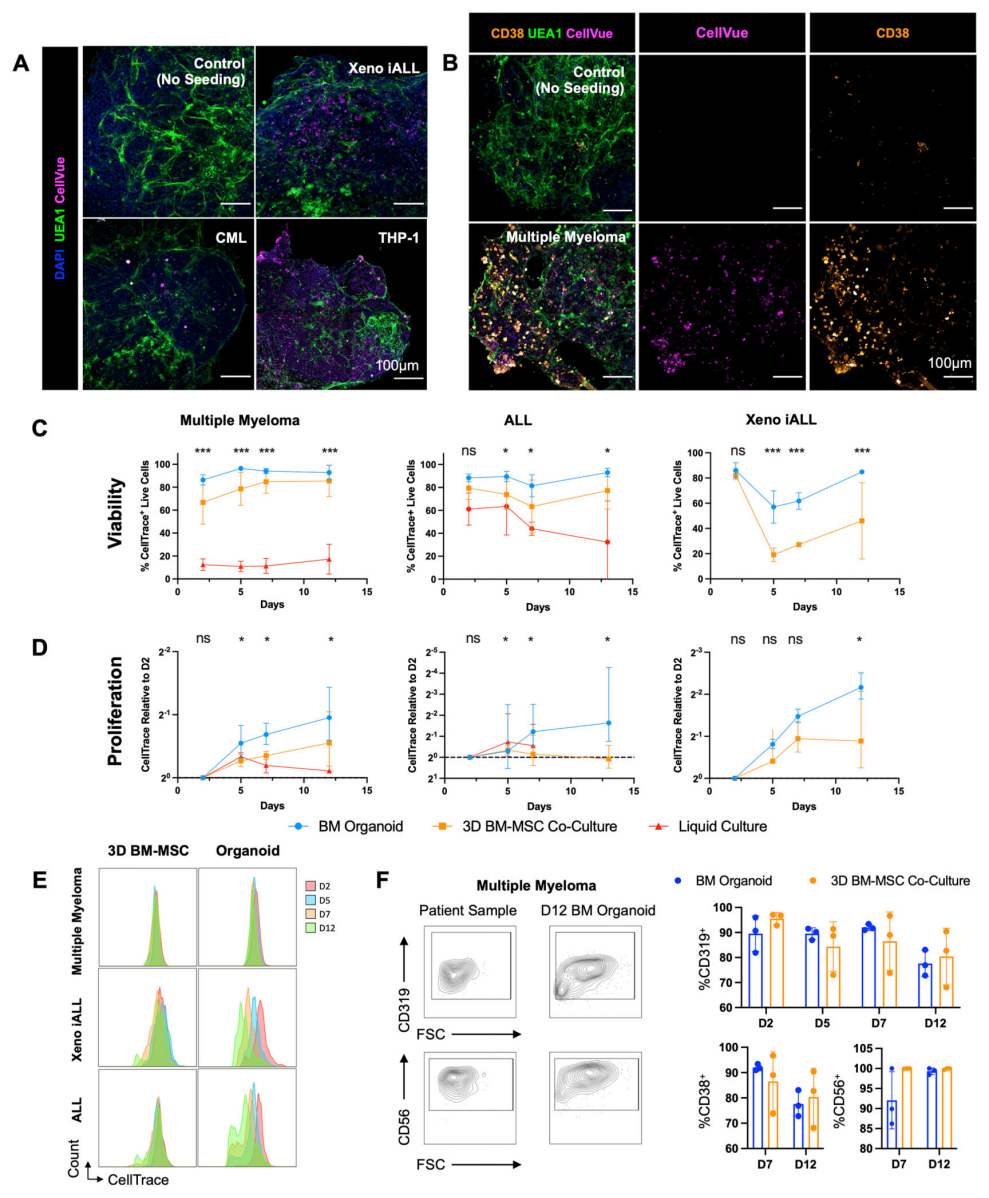
Bone marrow organoids support the engraftment, survival and proliferation of cells from patients with myeloid and lymphoid hematological malignancies. **(A)** Organoids engrafted with CellVue-labelled model infant acute lymphoblastic leukemia cells from xenografts (Xeno iALL), primary cells from a patient with untreated chronic myeloid leukemia (CML) and THP-1 cells, an acute myeloid leukemia cell line. CellVue^+^ cells are visible throughout the volume of organoids. **(B)** Organoids seeded with CD138^+^ cells isolated from bone marrow aspirates of patients with multiple myeloma show CellVue^+^ CD38^+^ plasma cell engraftment. **(C, D, E)** Viability and proliferation of cells from 4 donors with multiple myeloma, 6 donors with acute lymphoblastic leukemia (ALL) and 3 Xeno iALL samples seeded simultaneously in the organoids, a 3D co-culture with primary human bone marrow mesenchymal stromal cells (3D BM-MSC), and where possible, liquid culture. **(E)** Serial dilution of CellTrace label, indicating cell proliferation, for multiple myeloma, Xeno iALL and ALL cells in 3D BM-MSC and organoids on days 2, 5, 7 and 12 following thawing and plating. **(F)** Engrafted multiple myeloma cells retained their immunophenotype at d12, with more consistent maintenance of CD319 and CD38 in organoids than 3D BM-MSC. Representative images shown. * p < 0.01, ** p < 0.05, *** p < 0.001. *n* = 4 for multiple myeloma, *n = 3* for Xeno iALL and *n* = 3 for ALL, with each repeat comprised of a separate donor-Two-Way ANOVA with repeated measures and multiple comparisons (organoid cultures vs. 3D BM-MSC) (Fisher’s LSD) for ALL and multiple myeloma, multiple un-paired T-Test for Xeno iALL data.

To test this, cryopreserved cells were thawed and fluorescently labelled prior to seeding into 96-well plates containing organoids. Cells from patients with multiple myeloma (n=5, Suppl. Table 4), ALL (n=6, Suppl. Table 4), CML (n=2, Suppl. Table 3), a human AML cell line (THP-1) and leukemic cells from a human fetal liver derived infant acute lymphoblastic leukemia xenograft model (Xeno iALL; n=3, Suppl. Fig. 10) rapidly engrafted and were observed throughout the organoid volume (Figs. 7A & 7B). Distinct CellTrace-positive populations were detectable in the organoids for all 17 donor samples, confirming successful engraftment and survival of primary human and xenograft-derived cells over a 12-day time course (Suppl. Fig.11A). Primary multiple myeloma cells were costained for CD38, confirming that cells derived from the malignant plasma cell clone had successfully engrafted (Fig. 7B).

We compared the survival and proliferation of primary multiple myeloma, ALL and Xeno iALL cells engrafted in the organoids to cells seeded in wells with media alone or into a single-lineage 3D co-culture system containing primary human bone marrow MSCs in a Matrigel + collagen I hydrogel (3D BM-MSC). Whereas multiple myeloma cells were less than 20% viable only 48 hours after seeding in wells with media alone, in stark contrast, the myeloma cells expanded and remained >90% viable more than 12 days after engraftment into organoids for all 5 donors tested (Fig. 7C & 7D). Similarly, the survival of primary ALL cells was significantly improved in the organoids compared to liquid culture (Fig. 7C). The cell viability for ALL and Xeno iALL was also higher in the organoids than in 3D BM-MSC co-cultures (Fig. 7C), with higher proliferation rates (Fig. 7D & 7E). Plasma cells from myeloma patients showed minimal cell division in either model (Fig. 7D), but the cells retained their original immunophenotype (CD38^+^, CD319^+^, CD56^+^) more consistently in the organoids than in the 3D BM-MSC (Fig. 7F). Similarly, ALL and Xeno iALL cells showed improved maintenance of CD19 expression in the organoids as compared to 3D BM-MSC (Suppl. Fig. 12). Together, these data confirm that the organoids provide a supportive niche for the survival and growth of primary blood cancer cells from patients, including for cancer types that are otherwise poorly viable *ex vivo* and after cryopreservation.

## Discussion

Here we describe the development of a protocol generating vascularised bone marrow organoids that faithfully model key cellular, molecular and architectural features of myelopoietic bone marrow including stromal cells, lumen-forming vasculature and myeloid cell types. We demonstrate the utility of these organoids for modelling cancer-induced perturbations to the bone marrow niche and myelofibrosis.

Treatment of organoids with TGFβ, the primary cytokine driving myelofibrosis, induced organoid fibrosis, enabling target prioritization and screening of potential inhibitors. Fibrosis also occurred following engraftment of organoids with HSPCs from patients with myelofibrosis, but not healthy donors. The ability to reliably model bone marrow fibrosis is an important advance, as the lack of adequate *in vitro* and *in vivo* models currently hampers efficient pre-clinical validation of strategies aiming to reduce or prevent fibrosis, which is a huge unmet need for patients with myeloproliferative neoplasms and other blood cancers (45). As the organoids are highly reproducible and feasible to generate at scale in 96- or 384-well plate formats, this system presents an ideal platform for high-throughput target screens using pharmacological or genetic modulation.

We also show that cells from patients with both myeloid and lymphoid malignancies readily engraft and survive within the organoids, including cancer cell types that are notoriously difficult to maintain *ex vivo*. Remarkably, malignant cells from patients with multiple myeloma that had been cryopreserved and thawed prior to use were sustained by the organoids for 12 days, while rapidly losing viability when plated *in vitro* without stromal support. Maintenance of primary myeloma cells *ex vivo* and a method to study their interactions within a multi-cellular hematopoietic niche environment will enable pre-clinical pharmacogenomic screens and the study of disease mechanisms using primary cells from patients, which is currently a significant obstacle to translational research in this disease. Targeted genetic modification of organoids could be performed either by using lineage-specific promoters, or by modulating the differentiation protocol to generate certain cellular subsets (e.g. stroma) from wild-type iPSCs, and then assembling these with hematopoietic cells generated from a genetically modified parent iPSC line.

A key limitation of current human *ex vivo* bone marrow models has been a lack of sinusoidal-like endothelium, with many systems reliant on human umbilical vein endothelial cells (HUVEC) (13). We show here that the addition of VEGFC, recently shown to support the bone marrow perivascular niche (28) drives the generation of vasculature and supporting stroma that are specialized for hematopoietic support and phenocopy bone marrow sinusoidal endothelium. The resulting bone marrow organoids thereby present a unique opportunity to study the hematopoietic-niche crosstalk that underpins healthy hematopoiesis, and how perturbations to these regulatory interactions are permissive for the emergence and progression of cancers.

While this system offers a substantial advance in the field, in its current iteration, no osteoid lineage, lymphoid cells, smooth muscle cells or adipocytes are generated. Similarly, while we show a high degree of homology of the organoid vasculature to sinusoidal endothelium from human bone marrow, distinct arteriolar and sinusoidal endothelial subtypes are not present in the organoids. The current differentiation protocol was optimized primarily to study myeloid malignancies and cancer-associated bone marrow fibrosis, and refinement of the growth factor supplements may improve the maintenance of lymphoid malignancies. In addition, the introduction of re-circulating flow (46) may allow for the generation of organoids that mimic native bone marrow physiology more comprehensively. Despite these limitations, maintenance of cells from B-cell leukemias and plasma cell malignancies as well as myeloid cancers provides proof-of-principle that bone marrow organoids can be used to support a range of bone marrow cancers and cell types, paving the way for customization to support other relevant studies.

A protocol for ectopic implantation of human bone marrow MSC-derived ossicles has previously been shown to support engraftment of adult HSCs *in vivo* (47). Although the iPSC-derived organoids are not an *in vivo* system, they are substantially more efficient to generate than *in vivo* ossicles (weeks *vs*. months), and do not require human bone marrow or platelet lysates, which are hard to source and may induce experimental variability. The survival and continuous production of hematopoietic support factors in the absence of exogenous cytokine supplementation suggests that longer-term cultures may be possible. The implantation of organoids into mice has not yet been tested, but may allow for longer term studies in an *in vivo* setting.

The development of organoids has been transformative in other disease settings, e.g. cerebral, lung and kidney disease modelling (48). This platform may similarly be an enabling technology for the interrogation of disease mechanisms in hematological cancers as well as the development and testing of novel therapies using human cells in a tissue-relevant system. Importantly, this platform is likely to reduce reliance on animal models. Target identification and screening using a speciesspecific, clinically relevant *ex vivo* model that can incorporate primary cells from patients may accelerate and increase the success rate of clinical translation.

## Materials and Methods

### iPSC Culture and Differentiation

A Gibco Human Episomal iPSC (Thermo Fisher Scientific Cat#A18945) line was maintained in StemFlex medium (Thermo Fisher Scientific Cat # A3349401) and on Geltrex (Thermo Fisher Scientific Cat#A1569601)-coated 6-well plates. The iPSC line was karyotyped prior to use (23) and potency markers assessed upon expansion and freezing. A full, detailed description of passaging and differentiation protocols are included in supplementary materials and methods. In brief - for differentiations, iPSC were dissociated using EDTA when colonies were approximately 100 μm in diameter. The resulting iPSC aggregates were incubated overnight in StemFlex supplemented with RevitaCell in 6-well Co-star Ultra-Low Attachment plates (Corning Cat#3471) (day -1). After an overnight incubation, cells were collected and resuspended in Phase I medium (supplementary methods) and cultured at 5% O_2_ for 3 days (d0-3). Aggregated were then collected again in Phase II medium (supplementary methods). On d5 cells were collected by gravitation for hydrogel embedding. Hydrogels were composed of 60% collagen (either type I, type IV, or an equal parts type I+IV mix) and 40% Matrigel as detailed in supplementary methods. Fully polymerised gels with cell aggregates were then supplemented with Phase III media (supplementary methods). Media was replenished every 72 hours.

### Immunofluorescence staining

Sections were blocked using 2% Goat Serum (Thermo Fisher Scientific, Cat#31872) 1% Bovine Serum Albumin (BSA) (Sigma, Cat#A9418) prior to primary antibody labelling with antibody diluted in 1% BSA, sequential PBS washes, and secondary labelling with AlexaFluor conjugates. Whole organoid blocking solution was supplemented with Triton X100, Tween, and Sodium deoxycholate as described by Wimmer *et al* (21). Antibodies listed in Suppl. Table 5 and additional details in Suppl. Methods.

### Single-cell RNA-sequencing, data processing and analysis

Cryopreserved cells pooled from 15 organoids from 3 differentiations from both VEGFA and VEGFA+C protocols were processed for single cell sequencing as described in Suppl. Methods, and processed using the Chromium Single Cell 3’ library and Gel Bead Kits v3.1 (10x Genomics) as per kit instructions. Demultiplexed FASTQ files were aligned to the human reference genome (GRCh38/hg38) using standard CellRanger (version 6.0.1) ‘cellranger count’ pipeline (10x Genomics). SingCellaR (31) (https://supatt-lab.github.io/SingCellaR.Doc/) was used for the downstream analysis.

### Flow Cytometry

Organoids were dissociated for flow cytometry analysis using collagenase Type II (Sigma Aldrich, Cat#C6885) resuspended in HEPES buffer (Sigma Aldrich, Cat#H0887) at a concentration of 20mg/mL. Samples were collected by gravitation in a 15mL Falcon tube and washed 2x in PBS then resuspended in collagenase Type II. For dissociation, samples were incubated at 37°C for 5 minutes before trituration and a further 5 minute incubation. The dissociation reaction was stopped through the addition of PBS supplemented with FBS. 10 organoids were dissociated per flow cytometry experiment. Analysis was performed using either a Cyan Flow Cytometer (Beckman Coulter) or an Attune NxT. Single colour stained controls and fluorescence-minus-one (FMO) controls were used for all experiments, using antibodies as listed in Suppl. Table 5.

### TGFβ treatment to induce organoid fibrosis

Organoids were treated with TGFβ (Peprotech, Cat#100-21) at 10, 25, or 50ng/mL for 72 hours. At 72 hours all samples were approx.. 90% viable after collagenase digestion. Samples were then collected for either whole mount microscopy, paraffin/OCT embedding and sectioning, or qRT-PCR. 32 organoids were treated per replicate, of these 16 were spun down for RNA extraction for subsequent qRT-PCR. The remaining 16 organoids were fixed, with 8 taken for whole volume imaging, and 8 per repeat taken for embedding and sectioning. For drug treatment, samples were treated with DMSO or TGFβ at a concentration of 25ng/mL, and supplemented with an inhibitor as described (either JQ1 at 0.5μM and SB at 20μM).

### Quantitative Real-Time Polymerase Chain Reaction (qRT-PCR)

Whole organoids were processed using either the Micro RNEasy Kit (Qiagen, Cat#74004) or Qiagen Mini RNA isolation kit (Qiagen, Cat#74104) as per kit instructions. cDNA was prepared using the High Capacity cDNA Reverse Transcription Kit (Applied Biosystems, Cat#4368814) or EvoScript Universal cDNA Master (Roche, Cat#07912374001) and RT-PCR performed using PowerUp SYBR Green Master Mix reagent (Applied Biosystems, Cat#A25742) or TaqMan™ Universal PCR Master Mix (Applied Biosystems) (see Suppl. Table 6 for list of primers).

### Seeding of organoids and 3D BM-MSC cultures with hematopoietic cells

Peripheral blood and bone marrow samples were collected from healthy donors and patients with hematological malignancies following provision of written informed consent in accordance with the Declaration of Helsinki. All participants donated human tissue for research without receiving monetary compensation, and the studies were approved by an institutional review board (myelofibrosis, CML and G-CSF mobilized healthy apheresis donors - INForMed Study, University of Oxford [IRAS: 199833; REC 16/LO/1376]; Multiple myeloma - Oxford Radcliffe Biobank [Oxford Clinical Research Ethics Committee 09/H0606/5/5, project 16/A185]; ALL samples: REC *16/LO/2055’* [IRAS 179685]). Written informed consent was received from all the participants for the donation of human tissue.

For engraftments with CD34^+^ HSPCs, cryopreserved mononuclear cells were thawed and CD34^+^ viable cells FACS-isolated using a Becton Dickinson FACSAria™ Fusion Cell Sorter with 100nm nozzle into 1.5ml Eppendorf tubes prior to seeding in organoids. For inhibitor experiments, organoids were engrafted with CD34^+^ myelofibrosis cells and cultured for 7 days prior to the addition of inhibitors. Myeloma cells were selected from total bone marrow mononuclear cells using anti-CD138 magnetic bead enrichment (StemCell Technologies, Cat #17887) prior to cryopreservation. Xeno iALL cells were derived from a recently published xenograft model of infant ALL in which the t(4;11)/MLL-AF4 translocation was introduced into primary human fetal liver hematopoietic cells by CRISPR-Cas9 gene editing prior to transplantation into immunodeficient mice (49). All experiments were performed under a project licence approved by the UK Home Office under the Animal (Scientific Procedures) Act 1986 after approval by the Oxford Clinical Medicine Animal Welfare and Ethical Review Body; and in accordance with the principles of 3Rs (replacement, reduction and refinement) in animal research. Cells were harvested from bone marrow of leukemic mice at 17-18 weeks, and total bone marrow cells cryopreserved. Following thawing and prior to seeding in the organoids, human CD45^+^ were selected using magnetic microbeads (Miltenyi Biotech, Cat# 130-045-801). Over 90% of human CD45^+^ cells were CD19^+^ CD34^+^ lymphoblasts with a predominantly CD34^+^ProB phenotype (CD10-CD20-IgM/IgD^-^, Suppl. Fig. 10). Informed consent was provided by all participants for the donation of human tissue, and this study was approved by an institutional review board (REC: 18/NE/0290 and 18/LO/0822). The replicates included in this study were from 4 mice transplanted with MLL-AF4 edited cells from one human fetal liver sample. Details of donor cell labelling are included in supplementary materials and methods. The composition of the engrafted organoids was analyzed by flow cytometry using either an LSR Fortessa X50 (BD Biosciences) or an Attune NxT or an NGS panel (see Suppl. Methods and Suppl. Table 7).

For the 3D BM MSC co-cultures, 24-well plates were prepared containing 300μl of a 70:30 mix of collagen I:Matrigel per well and incubated at 37°C for 90 minutes. Primary BM MSCs re-suspended at 10,000 cells/mL in StemPro-34 supplemented medium were added to the wells (500 μl/well) and incubated overnight prior to seeding with primary hematopoietic cells.

### Data Analysis Software

scRNAseq analyses were performed in R Studio (version 1.4.1106). Other statistical analyses were performed using Graph Pad PRISM 7 with statistical tests as described in relevant figure legends. *P* values defined as * p < 0.01, ** p < 0.05, *** p < 0.001, **** p < 0.0001.

## Supplementary Material

Supplementary material

Table S1

Table S2

Table S3

Table S4

Table S5

Table S6

Table S7

## Data Availability

Single cell RNA sequencing data is available at GEO (accession GSE19668). Scripts used for analysis are available at (https://github.com/aokhan/BMorganoidV1/) and (https://github.com/supatt-lab/SingCellaR). Further data is available on request.

## Significance Statement

We present a human bone marrow organoid that supports the growth of primary cells from patients with myeloid and lymphoid blood cancers. This model allows for mechanistic studies of blood cancers in the context of their microenvironment, and provides a much-needed*, ex vivo* tool for the prioritization of new therapeutics.
