# Supplementary material for: Human bone marrow organoids for disease modelling, discovery and validation of therapeutic targets in hematological malignancies
Source: Cancer Discov. Author manuscript; Available in PMC 2023 Feb 9. (PMC9900323; doi:10.1158/2159-8290.CD-22-0199)
Supplement: Supplementary material [file EMS157157-supplement-Supplementary_material.pdf]

Supplementary Material

Human bone marrow organoids for disease modelling, discovery and validation of therapeutic targets in hematological malignancies

Abdullah O. Khan*[1,2], Antonio Rodriguez-Romera[2], Jasmeet S. Reyat[1], Aude-Anais Olijnik[2], Michela Colombo[2], Guanlin Wang[2,3], Wei Xiong Wen[2,3], Nikolaos Sousos[2,4], Lauren C. Murphy[2], Beata Grygielska[1], Gina Perrella[1], Chris Mahoney[5], Rebecca E. Ling[6], Natalina E. Elliott[6], Christina Simoglou Karali[2], Andrew P. Stone[10], Samuel Kemble[5], Emily A. Cutler[9], Adele K. Fielding[9], Adam P. Croft[5], David Bassett[8], Gowsihan Poologasundarampillai[7], Anindita Roy[6], Sarah Gooding[2], Julie Rayes[1], Kellie R. Machlus*[10], Bethan Psaila*[2,4].

1. Institute of Cardiovascular Sciences, College of Medical and Dental Sciences, University of Birmingham, Vincent Drive, Birmingham, U.K, B15 2TT
2. MRC Weatherall Institute of Molecular Medicine, Radcliffe Department of Medicine and National Institute of Health Research (NIHR) Oxford Biomedical Research Centre, University of Oxford, Oxford, U.K. OX3 9DS
3. Centre for Computational Biology, MRC Weatherall Institute of Molecular Medicine, University of Oxford, Oxford, U.K. OX3 9DS.
4. Cancer and Haematology Centre, Churchill Hospital, Oxford University Hospitals NHS Foundation Trust, Oxford, U.K. OX3 7LE.
5. Rheumatology Research Group, Institute of Inflammation and Ageing, College of Medical and Dental Sciences, University of Birmingham, Vincent Drive, Birmingham, U.K. B15 2TT.
6. MRC Weatherall Institute of Molecular Medicine, Department of Paediatrics and National Institute of Health Research (NIHR) Oxford Biomedical Research Centre, University of Oxford, Oxford, U.K. OX3 9DS
7. School of Dentistry, Institute of Clinical Sciences, University of Birmingham, Birmingham, U.K. B5 7EG.
8. Healthcare Technologies Institute, School of Chemical Engineering, University of Birmingham, Birmingham, B15 2TT, U.K.
9. University College London Cancer Institute, 72 Huntley Street, London WC1E 6DD
10. Vascular Biology Program, Boston Children’s Hospital, Department of Surgery, Harvard Medical School, Boston, MA 02115, USA.

*Correspondence to a.khan.4@bham.ac.uk, Kellie.Machlus@childrens.harvard.edu and bethan.psaila@ndcls.ox.ac.uk

Supplementary Materials and Methods

iPSC Culture and Differentiation

A Gibco Human Episomal iPSC (Thermo Fisher Scientific Cat#A18945) line was maintained in StemFlex medium (Thermo Fisher Scientific Cat # A3349401) and on Geltrex (Thermo Fisher Scientific Cat#A1569601)-coated 6-well plates. The iPSC line was karyotyped prior to use (1) and potency markers assessed upon expansion and freezing. Cells were passaged as clumps using EDTA at 0.02% in PBS (0.5mM, Sigma Cat#E8008), and were freshly thawed or passaged for differentiation and maintained in StemFlex supplemented with RevitaCell (Thermo Fisher Scientific Cat#2644501). Cultures were maintained at 37°C and 5% CO_2. Differentiations were initiated with iPSCs between passages 5 and 30.

For differentiations, iPSC were dissociated using EDTA when colonies were approximately 100 μm in diameter. The resulting iPSC aggregates were incubated overnight in StemFlex supplemented with RevitaCell in 6-well Costar Ultra-Low Attachment plates (Corning Cat#3471) (day -1). After an overnight incubation, cells were collected by gravitation in a 15mL Falcon tube (Fisher Scientific Cat#11507411) and resuspended in Phase I medium comprised of APEL2 (StemCell Technologies Cat#05275)(2) supplemented with Bone Morphogenic Protein-4 (BMP4, Thermo Fisher Scientific Cat#PHC9531), Fibroblast Growth Factor-2 (FGF2, StemCell Technologies Cat#78134.1), Vascular Endothelial Growth Factor-A (VEGF-165, StemCell Technologies Cat#78159.1) at 50ng/mL, plated in a 6-well ULA plates and incubated at 5% O_2 for 3 days (d0-3).

Cell aggregates were then collected by gravitation and re-suspended in Phase II medium for a further 48 hours (d3-5). Phase II medium (APEL2 supplemented with BMP-4, FGF2, and VEGFA at 50ng/mL; human Stem Cell Factor (hSCF, StemCell Technologies Cat#78062) and Fms-like tyrosine kinase-3 Ligand (Flt3, StemCell Technologies Cat#78009) at 25ng/mL.

On d5 cells were collected by gravitation for hydrogel embedding. Hydrogels were composed of 60% collagen (either type I, type IV, or an equal parts type I+IV mix) and 40% Matrigel. Hydrogels were prepared on ice as per manufacturer's instructions and were comprised of Reduced Growth Factor Matrigel (Corning, Cat#354230) supplemented with 1mg/mL human collagen type I (Advanced Biomatrix, Cat#5007) and human collagen type IV (Advanced Biomatrix, Cat#5022) as per designated gel composition. Hydrogel mixes were neutralised with 1N NaOH. An 0.5mL cell-free base layer was added and allowed to polymerise for 2 hours, before a further 0.5mL layer of gel supplemented with gravitated cell aggregates was added and also left to polymerise for 2 hours at 37°C and 5%CO_2. Fully polymerised gels with cell aggregates were then supplemented with Phase III media comprised of VEGFA at either 50ng or 25ng/mL, VEGFC (where relevant) at 50 or 25ng/mL, FGF2, BMP4, hSCF, Flt3, Erythropoeitin (EPO, StemCell Technologies, Cat#78007), Thrombopoeitin (TPO, StemCell Technologies, Cat#78210), Granuolocytic Colony-Stimulating

Factor (G-CSF, StemCell Technologies, Cat#78012), at 25ng/mL, and Interleukin-3 (IL3, StemCell Technologies, Cat#78194) and Interleukin-6 (IL6, StemCell Technologies, Cat#78050) at 10ng/mL. Media was replenished every 72 hours.

Immunofluorescence staining

Sections were blocked using 2% Goat Serum (Thermo Fisher Scientific, Cat#31872) 1% Bovine Serum Albumin (BSA) (Sigma, Cat#A9418) prior to primary antibody labelling with antibody diluted in 1% BSA, sequential PBS washes, and finally secondary labelling with AlexaFluor conjugates. Whole organoid blocking solution was further supplemented with Triton X100, Tween, and Sodium deoxycholate as described by Wimmer *et al* (3).

Sprouting organoids were imaged within hydrogels in 8-well microslides (Ibidi, Cat#80806), whole organoids were labelled in 15mL Falcons before embedding in 0.5% Agarose within 8-well microslides. Whole organoids were subject to serial dehydration (50%, 70%, 90%, 100%) within microslides before clearance with Ethyl Cinnamate and subsequent imaging. Sections were prepared by embedding fixed organoids in Optimal Cutting Temperature compound (OCT, VWR Cat#361603E) before sectioning onto Poly-L-Lysine covered slides. Slides were washed in Acetone before immunofluorescence labelling.

Microscopy and Image Analysis

Confocal microscopy was performed using a Zeiss LSM880 confocal AiryScan microscope with either a 25X LD LCI plan apo 0.8 NA dual immersion (420852-9871-000) or 40x C-APO NA 1.2 water immersion objective (421767-9971-711) as described previously(1). Confocal images were acquired as representative Z-stacks (with Z-resolution set to Nyquist requirements), and presented as maximum intensity projections (Fiji)(4) where stated. Histological preparations (reticulin and H&E, details provided in supplementary materials and methods) were imaged using a Zeiss AxioScan.Z1 slide scanner. Image analysis was performed in Fiji. For measurements of sprout radii, brightfield images acquired on an Evos (Thermo Fisher Scientific) desktop microscope. Sprout radii were measured manually by drawing and measuring a line from the centre to the tip of the sprout across 3 independent biological replicates, with between 30-50 sprouts measured per replicate. To measure the proximity of megakaryocytes to organoid blood vessels, 250µm x 50µm volumes of individual organoids were acquired using cleared whole mount organoids imaged by confocal microscopy, as previously described. CD41 labelled megakaryocytes within 5µm of UEA1 labelled vessels were counted as ‘vessel-associated MKs’ within a maximum intensity projection of each imaged volume (5).

Single-cell RNA-sequencing

Cryopreserved cells pooled from 15 organoids from 3 differentiations from both VEGFA and VEGFA+C protocols were thawed, stained with DAPI to exclude non-viable cells, and DAPI- live cells sorted on a Becton Dickinson Aria Fusion with 100nm nozzle as per recommendations in the 10x Genomics Single Cell Protocols – Cell Preparation Guide. 10,000 live cells per sample were sorted into 2µL PBS/0.05% BSA (non-acetylated) and the cell number/volume adjusted to the target for loading onto the 10x Chromium Controller. Samples were processed according to the 10x protocol using the Chromium Single Cell 3′ library and Gel Bead Kits v3.1 (10x Genomics). Cells and reagents were prepared and loaded onto the chip and into the Chromium Controller for droplet generation. Reverse transcription was conducted in the droplets and cDNA recovered through demulsification and bead purification. Pre-amplified cDNA was used for library preparation, multiplexed and sequenced on a Novaseq 6000. Details on data processing ana

scRNAseq data processing and analysis

Demultiplexed FASTQ files were aligned to the human reference genome (GRCh38/hg38) using standard CellRanger (version 6.0.1) 'cellranger count' pipeline (10x Genomics). SingCellaR (6) (https://supatt-lab.github.io/SingCellaR.Doc/) was used for the downstream analysis. Data was first subject to quality control with the maximum percentage of mitochondrial genes, maximum detected genes and max number of UMIs set to 12%, 6,000, and 50,000, respectively. Minimum detected genes and UMIs were set to 300 and 500, respectively and genes with minimum expressing cells was set as 10. Raw expression matrix was then normalised and scaled and number of UMIs and percentage of mitochondrial reads were regressed out before a general linear model (GLM) was used to identify highly variable genes(7). Data were then subject to downstream analyses including principal component analysis (PCA), UMAP analysis (top 40 PCs were used, and n.neighbour = 120), and clustering using the Louvain method. Differentially expressed genes were calculated using 'identifyDifferentialGenes' function (min.log2FC = 0.3 and min.expFraction = 0.25). To compare cells from the two experimental conditions (VEGFA only and VEGFA+C), cells were down-sampled so that each cell group had the same number of cells. Wilcoxon test of normalized UMIs was used to compare the gene expressions and Fisher's exact test was used to compare the cell frequency. The resulting *P values* from both tests were combined using Fisher's method and subsequently adjusted by Benjamini-Hochberg correction. 'runFA2_ForceDirectedGraph' function was used to identify the trajectories.

CellPhoneDB v 2.1.1 (https://github.com/Teichlab/cellphonedb) was performed for ligand-receptor interactions using normalized expression matrix of VEGFA +C as detailed by Garcia-Alonso *et al.* (8,9). Cell-cell interaction network between the different cell clusters from VEGFAC and Sankey plot demonstrating the interaction between TGFβ1, CXCL12, and CD44 ligands with their responding

receptors from VEFGA and VEFGAC were plotted using a modified version of the CrossTalkeR R package (version 1.2.1) (10).

We applied Symphony (11) to map cells from VEGFA+C organoids to published scRNAseq datasets from human bone marrow (12) and fetal liver and bone marrow cells (6,13) respectively. For the human bone marrow dataset, we first built the reference data using the normalized expression matrix using ‘symphony::buildReference’. For the fetal liver dataset we used the pre-built reference provided by the Symphony developer. The ‘mapQuery’ and ‘knnPredict’ function were used to map the VEGFA+C cells onto the three reference datasets.

Histology

Organoids were fixed in neutral buffered formalin (Sigma-Aldrich, Cat#HT501128-4L) in a 15mL Falcon tube, washed twice with PBS, and then subject to serial dehydration (30%, 50%, 70%, 100%) in ethanol before immersion in Histoclear (Geneflow, Cat#A2-0101). Samples were then embedded in paraffin and sent as blocks to C&C laboratories for staining and mounting.

CellTrace labelling for viability and proliferation assays

Primary cells were labelled with CellTrace Far Red as indicated by the manufacturer. Briefly, cells were washed 1X with PBS and resuspended at $1x10^6$ cells/mL in staining solution (CellTrace Far Red 2μM in PBS). Cells were incubated in staining solution for 30min at 37°C. After incubation CellTrace was quenched with 5 volumes of PBS with FBS (10%), spun down and resuspended in the appropriate media.

Quantitative Real-Time Polymerase Chain Reaction (qRT-PCR)

Whole organoids were processed using either the Micro RNEasy Kit (Qiagen, Cat#74004) or Qiagen Mini RNA isolation kit (Qiagen, Cat#74104) according to the manufacturer’s instructions. Isolated RNA was quantified on the NanoDrop ND-100 (Thermo Scientific) and cDNA was prepared using the High Capacity cDNA Reverse Transcription Kit (Applied Biosystems, Cat# 4368814) or EvoScript Universal cDNA Master (Roche, Cat#07912374001) according to the manufacturer’s instructions using standard cycling conditions. cDNA was diluted to 5ng before being combined with PowerUp SYBR Green Master Mix reagent (Applied Biosystems, Cat# A25742) and the relevant PrimeTime qRT-PCR primers (IDT), or performed using TaqMan™ Universal PCR Master Mix (Applied Biosystems) on StepOne plus machine (Applied Biosystem) (see Suppl. Table 6 for a full list of primers). The absolute expression of the respective genes was calculated using the ∆Ct method using *GAPDH* as an internal housekeeping control.

Luminex Assays

To assess the production of growth factors, organoids were washed and cultured in StemPro-34 (L-Glutamine only) without any added supplements or growth factors for 12 days. 50:50 media

changes were performed at 72 hour intervals, and media was collected for Luminex assays at day 12. Supernatant from 12 organoids was collected and pooled for each repeat.

Luminex kits (LXSHAM-03, LXSAHM-28) were used for multiplexed proteomic assays as per the manufacturer instructions. No detectable signal was observed in cell-free medium.

Mutation detection by next generation sequencing (NGS)

A custom-made, targeted, hybridization, ultra-deep, next-generation sequence panel for the detection of a panel of commonly mutated genes in clonal hematopoiesis and myeloproliferative neoplasms was used to screen for mutations in samples from myelofibrosis patients post- organoid engraftment (14,15). The combined probe footprint size of the panel was 25,083 bp, and the exact target regions are listed in Suppl. Table 7. Library preparation was performed as per the Twist NGS Workflow manufacturer instructions, including enzymatic DNA fragmentation, end repair and dA-tailing; universal adapters ligation; PCR amplification using UDI primers; bead-based, capture probes - pool hybridization; and post-hybridisation target enrichment. Sequencing was carried out on the Illumina NextSeq™ 500 System, using the NextSeq™ 500/550 Mid Output Kit v2.5 (150 Cycles).

Pre-processing of DNA-sequencing data

FASTQ files were pre-processed for downstream variant detection as previously described.(6, 7) Nucleotide bases at the 3'-end of the sequencing reads with Phred score < 20 were trimmed using TrimGalore (version 0.6.5).(16). Trimmed reads were aligned to the human reference genome (GRCh38 build) using Burrows-Wheeler Aligner (version 0.7.17)(17). The resulting Sequence Alignment Map (SAM) were converted to Binary Alignment Map (BAM) and indexed using Samtools (version 1.9)(18). The sample IDs were added to the sequencing reads using the *AddorReplaceReadGroups* module from Picard (version 2.3.0). Next, duplicate reads were marked and removed using the *MarkDuplicates* module from Picard. The base quality scores were subsequently recalibrated based on known single-nucleotide and indel polymorphism sites using the *BaseRecalibrator* and *ApplyBQSR* modules from The Genome Analysis Toolkit (GATK) (version 4.2.0.0)(19).

Variant detection, annotation, and filtering

Variant detection was performed using *Mutect2* module from GATK with the following options to enable detection of variants with low variant allele frequency (VAF) (20): --tumor-lod-to-emit 2.0 --min-base-quality-score 20 --disable-read-filter MateOnSameContigOrNoMappedMateReadFilter --mitochondria-mode --callable-depth 10 --max-reads-per-alignment-start 0 --annotation AS_FisherStrand --annotation AS_QualByDepth --annotation FisherStrand --annotation QualByDepth. Then, functional annotation of variants was performed using ANNOVAR (21), and the

flanking 10bp nucleotides of the variants were retrieved using BEDTools (version 2.29.2) (22). Finally, the following variants were excluded: (1) germline polymorphisms; (2) variants located on the non-coding regions; (3) variants located within homopolymer regions defined as regions with 6 or more consecutive identical nucleotides; (4) occurrence of at least 3 variants that are within a window of 20 bases; (5) variants that do not alter the open reading frame such as non-frameshift indels and synonymous variants; (6) variants with fisher strand bias > 20; (7) variants supported by less than 5 reads and subsequently (8) variant supported by less than 10 reads, and (9) variants with VAF <1%. Coverage analysis for this run showed a median alignment rate of 99.5%, and sequencing depth of > 1 $x10^6$ reads for all samples (median 4.1 $x10^6$), with most probes demonstrating > 1,000x coverage.

Viability and proliferation assays

Primary cells were labelled with CellTrace Far Red as per kit instructions. Briefly, cells were washed and resuspended at $1x10^6$ cells/mL in staining solution for 30min at 37°C. After incubation, CellTrace was quenched with 5 volumes of PBS with 10% FBS.

Donor cell labelling

Prior to seeding, donor cells were labelled with CellVue Claret Far Red Fluorescent Cell Linker Mini Kit for General Membrane Labelling (Sigma Aldrich, Cat#MINCLARET-1KT) following manufacturer instructions. For seeding of organoids, each well of a 96-well plate containing 1-2 individual organoids or media alone were seeded with 5000 cells per well and cultured for up to 14 days in StemPro (Thermo Fisher Scientific, Cat#10639011) supplemented with Phase IV cytokines. Wells seeded with iALL cells were further supplemented with IL7, with 50% media changes every 2-3 days.

References

1. Khan AO, Slater A, Maclachlan A, Nicolson PLR, Pike JA, Reyat JS, et al. Post-translational polymodification of β1-tubulin regulates motor protein localisation in platelet production and function. Haematologica. 2020;Online ahead of print:0–0.

2. Ng ES, Azzola L, Bruveris FF, Calvanese V, Phipson B, Vlahos K, et al. Differentiation of human embryonic stem cells to HOXA+ hemogenic vasculature that resembles the aorta-gonad-mesonephros. Nat Biotechnol. 2016;34:1168–79.

3. Wimmer RA, Leopoldi A, Aichinger M, Wick N, Hantusch B, Novatchkova M, et al. Human blood vessel organoids as a model of diabetic vasculopathy. Nature. 2019;565:505–10.

4. Schindelin J, Arganda-Carreras I, Frise E, Kaynig V, Longair M, Pietzsch T, et al. Fiji: an open-source platform for biological-image analysis. Nat Methods. 2012;9:676–82.

5. Stegner D, vanEeuwijk JMM, Angay O, Gorelashvili MG, Semeniak D, Pinnecker J, et al. Thrombopoiesis is spatially regulated by the bone marrow vasculature. Nat Commun. 2017;8:127.

6. Roy A, Wang G, Iskander D, O'Byrne S, Elliott N, O'Sullivan J, et al. Transitions in lineage specification and gene regulatory networks in hematopoietic stem/progenitor cells over human development. Cell Reports. 2021;36:109698.

7. Brennecke P, Anders S, Kim JK, Kołodziejczyk AA, Zhang X, Proserpio V, et al. Accounting for technical noise in single-cell RNA-seq experiments. Nat Methods. 2013;10:1093–5.

8. Garcia-Alonso L, Handfield L-F, Roberts K, Nikolakopoulou K, Fernando RC, Gardner L, et al. Mapping the temporal and spatial dynamics of the human endometrium in vivo and in vitro. Nat Genet. 2021;53:1698–711.

9. Efremova M, Vento-Tormo M, Teichmann SA, Vento-Tormo R. CellPhoneDB: inferring cell–cell communication from combined expression of multi-subunit ligand–receptor complexes. Nat Protoc. 2020;15:1484–506.

10. Nagai JS, Leimkühler NB, Schaub MT, Schneider RK, Costa IG. CrossTalkeR: Analysis and Visualisation of Ligand Receptor Networks. Bioinformatics. 2021;37:btab370-.

11. Kang JB, Nathan A, Weinand K, Zhang F, Millard N, Rumker L, et al. Efficient and precise single-cell reference atlas mapping with Symphony. Nat Commun. 2021;12:5890.

12. Jong MME de, Kellermayer Z, Papazian N, Tahri S, Bruinink DH op, Hoogenboezem R, et al. The multiple myeloma microenvironment is defined by an inflammatory stromal cell landscape. Nat Immunol. 2021;22:769–80.

13. Popescu D-M, Botting RA, Stephenson E, Green K, Webb S, Jardine L, et al. Decoding human fetal liver haematopoiesis. Nature. 2019;574:365–71.

14. Watson CJ, Papula AL, Poon GYP, Wong WH, Young AL, Druley TE, et al. The evolutionary dynamics and fitness landscape of clonal hematopoiesis. Science. 2020;367:1449–54.

15. Greenfield G, McMullin MF, Mills K. Molecular pathogenesis of the myeloproliferative neoplasms. J Hematol Oncol. 2021;14:103.

16. Martin M. Cutadapt removes adapter sequences from high-throughput sequencing reads. Embnet J. 2011;17:10–2.

17. Li H, Durbin R. Fast and accurate short read alignment with Burrows–Wheeler transform. Bioinformatics. 2009;25:1754–60.

18. Li H, Handsaker B, Wysoker A, Fennell T, Ruan J, Homer N, et al. The Sequence Alignment/Map format and SAMtools. Bioinformatics. 2009;25:2078–9.

19. McKenna A, Hanna M, Banks E, Sivachenko A, Cibulskis K, Kernytsky A, et al. The Genome Analysis Toolkit: A MapReduce framework for analyzing next-generation DNA sequencing data. Genome Res. 2010;20:1297–303.

20. Schischlik F, Jäger R, Rosebrock F, Hug E, Schuster M, Holly R, et al. Mutational landscape of the transcriptome offers putative targets for immunotherapy of myeloproliferative neoplasms. Blood. 2019;134:199–210.

21. Wang K, Li M, Hakonarson H. ANNOVAR: functional annotation of genetic variants from high-throughput sequencing data. Nucleic Acids Res. 2010;38:e164–e164.

22. Quinlan AR, Hall IM. BEDTools: a flexible suite of utilities for comparing genomic features. Bioinformatics. 2010;26:841–2.

Supplementary Figure 1 (relating to Figure 1)

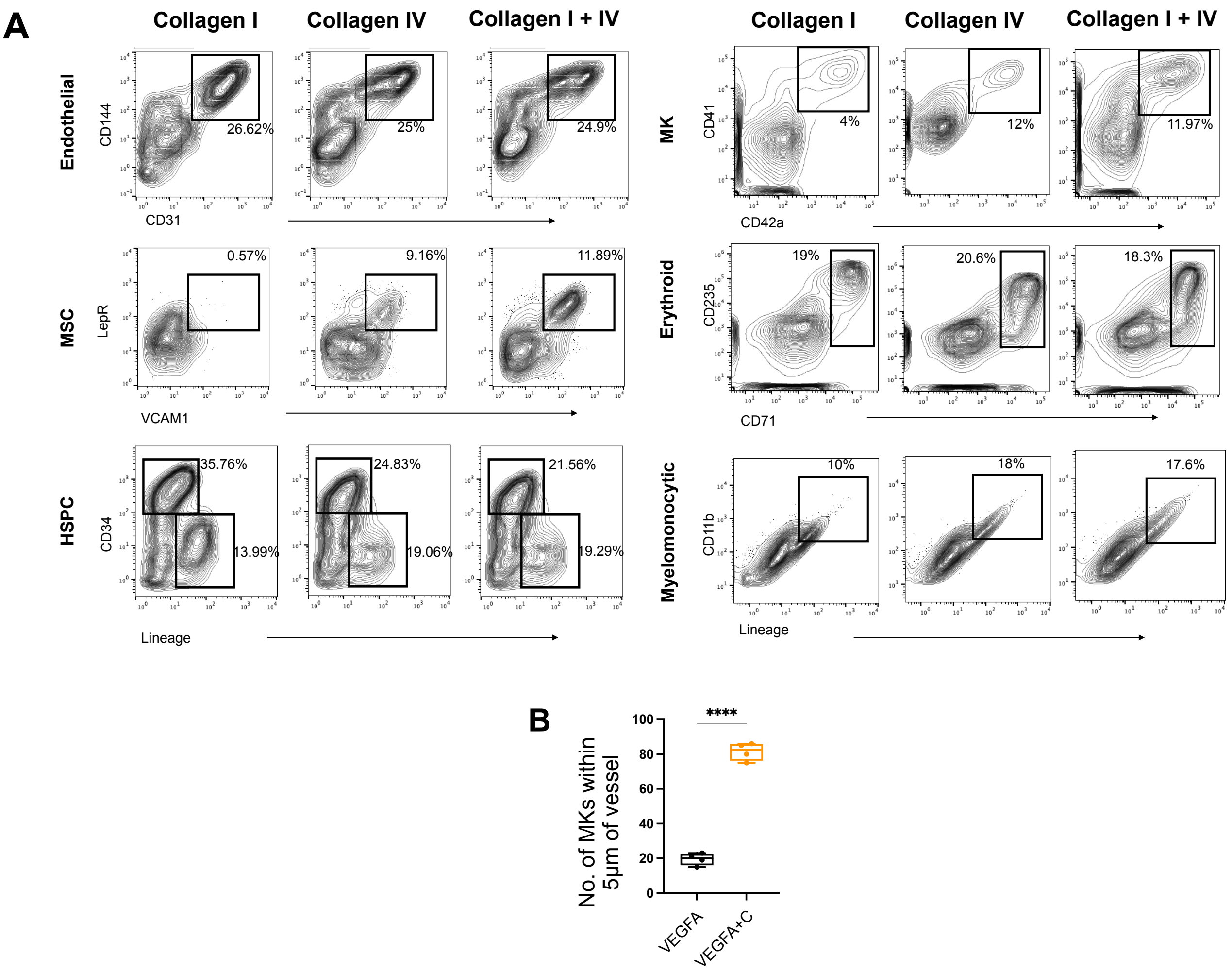

Supplementary Figure 1, relating to Figure 1: Representative flow cytometry plots for characterization of organoids differentiated in collagen I, collagen IV and collagen I+IV Matrigel hydrogels. (A) Gating strategy for analysis of hematopoietic and stromal cell types is shown. Abbreviations: Hematopoietic stem and progenitor cells (HSPC); mesenchymal stromal cells (MSC), megakaryocytes (MK). **(B)** Measurement of the number of megakaryocytes (MKs) in close proximity to vessels in VEGFA and VEGFA+C derived organoids. Each datapoint represents number of MKs within 5µm of a vessel in a 250µm^2 region of interest.

Supplementary Figure 2 (relating to Figure 3)

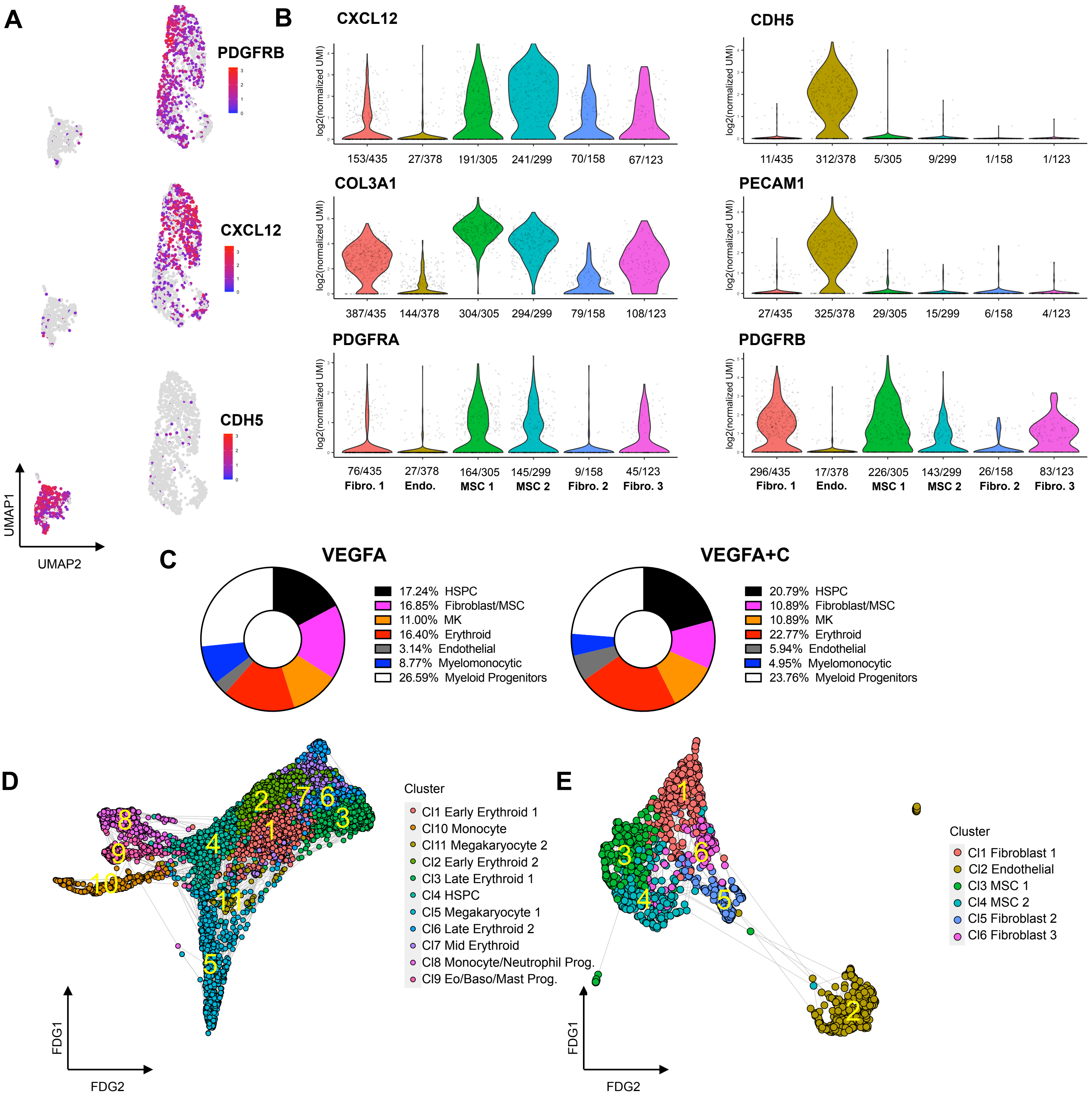

Supplementary Figure 2, relating to Figure 3: Expression of canonical genes, relative abundance and differentiation trajectories. (A) Uniform Manifold and Approximation Projection (UMAP) plots showing expression of *PDGFRB* and *CXCL12* in fibroblasts and mesenchymal stromal cells (MSC) and *CDH5* in endothelial cell clusters of VEGFA+C bone marrow organoids respectively. **(B)** Violin plots showing expression of key genes in stromal cell clusters (*CXCL12, COL3A1, PDGFRA/PDGFRB* in MSC/fibroblasts (Fibro), *CDH5* and *PECAM1* in endothelial cells (Endo). Number of cells in each cluster in which expression of gene was detected is indicated below the plot. **(C)** Relative abundance of cell types in VEGFA and VEGFA+C derived organoids. **(D & E)** Force-directed graph (FDG) of **(D)** hematopoietic and **(E)** stromal cell compartments in VEGFA+C organoids.

Supplementary Figure 3 (relating to Figure 3)

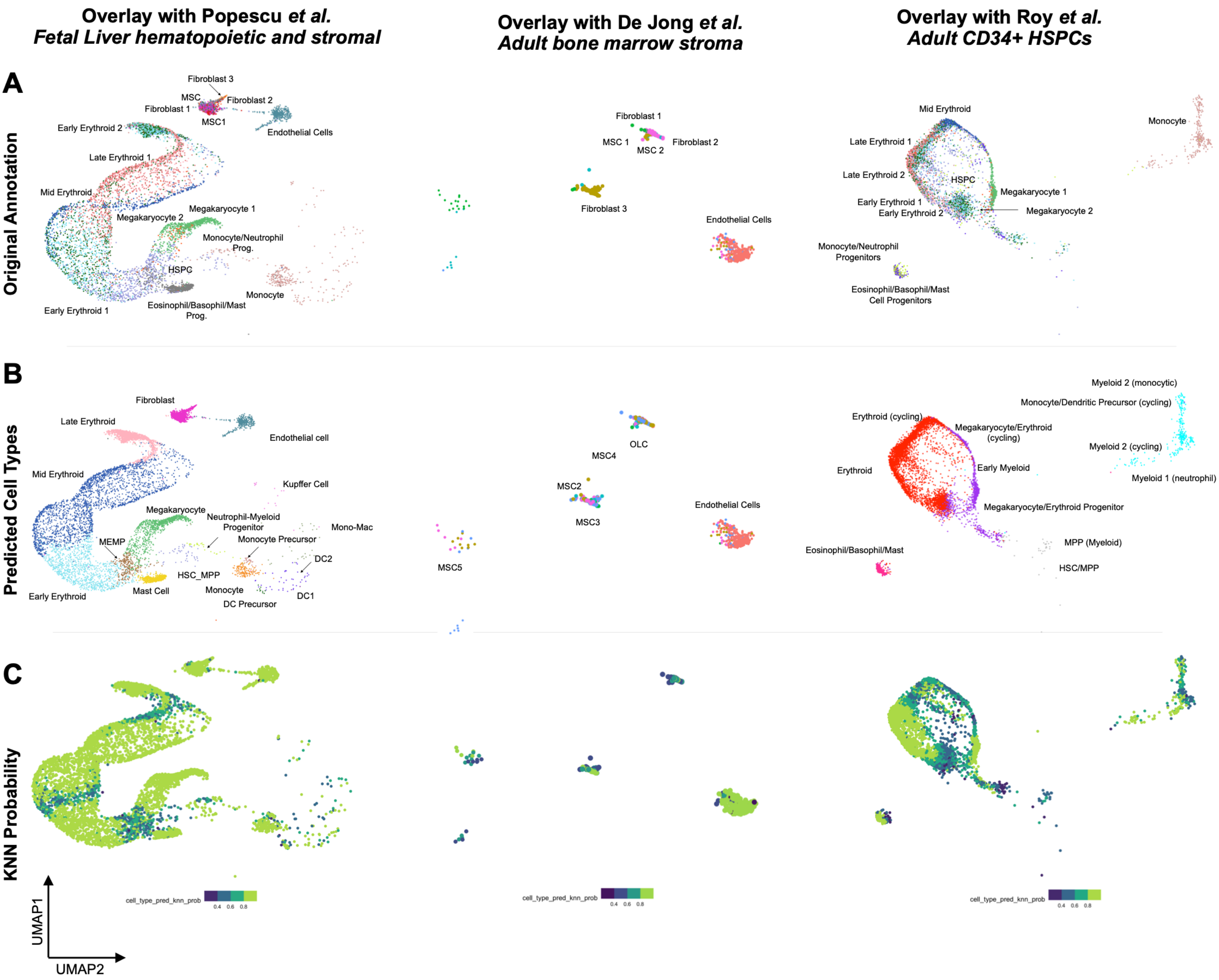

Supplementary Figure 3, relating to Figure 3: Overlay of VEGFA+C derived bone marrow organoid cells to published datasets of human hematopoietic and stromal cells using the Symphony package. (A) Original annotation as per Figure 3. **(B)** Predicted cell types, according to matching to the relevant reference dataset. **(C)** KNN score, representing the spearman correlation score between the cells in the reference and query datasets, where a high score indicates good correlation between cell types.

Supplementary Figure 4 (relating to Figure 3)

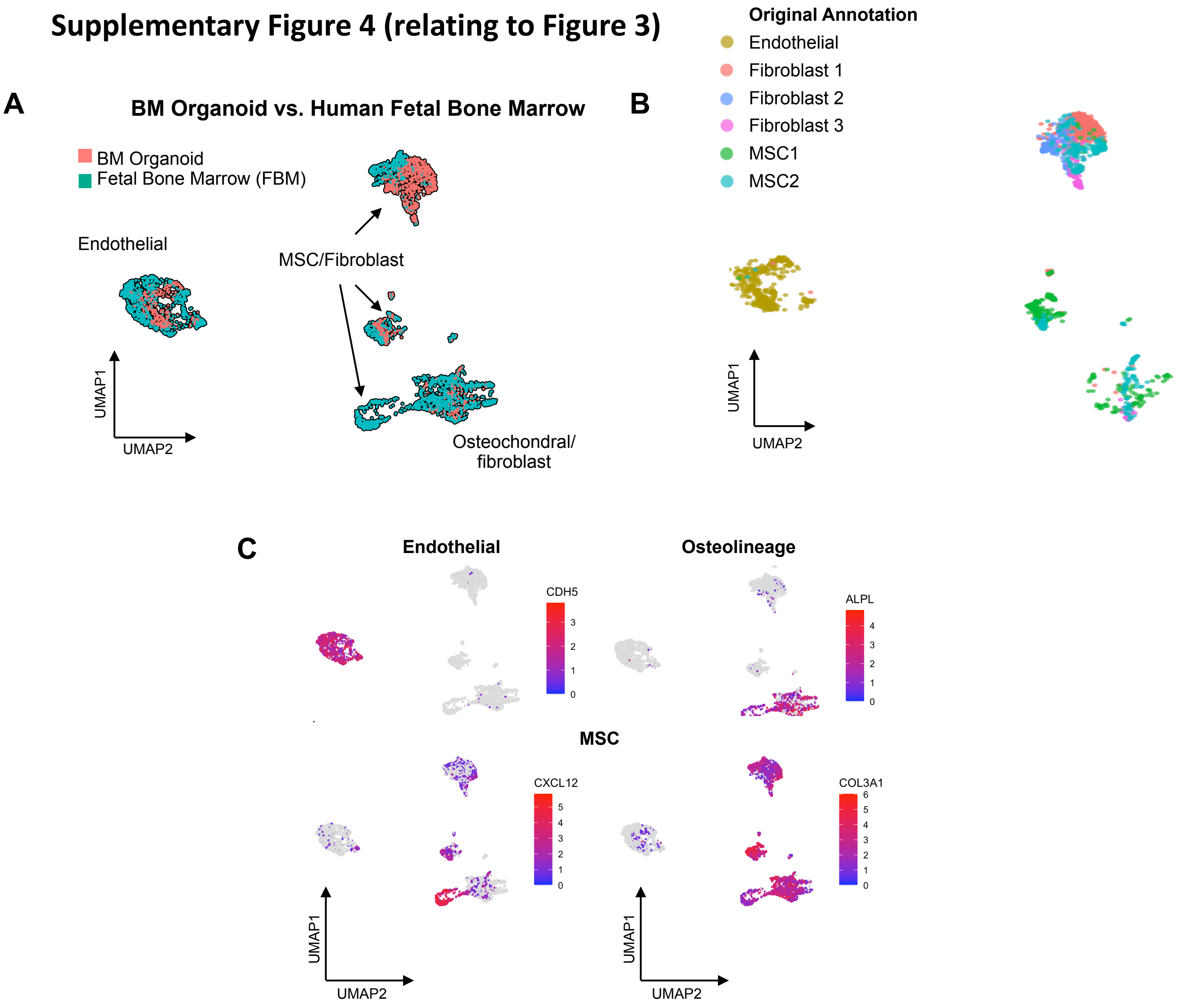

Supplementary Figure 4, relating to Figure 3: Integration of bone marrow organoid stromal cells with fetal bone marrow stroma. (A) Organoid stromal cells (mesenchymal stromal cells (MSCs), fibroblasts and endothelial cells) were integrated with relevant stromal cell clusters from a dataset of fetal bone marrow (Jardine et al, Nature 2021). Endothelial and MSC/fibroblast populations from organoids and native human bone marrow cluster together. **(B)** UMAP of integrated data sets with original VEGFA+C organoid cell annotations shown. **(C)** Integrated UMAPs were annotated using key features with clusters assigned based on the expression of canonical markers e.g. *CDH5* for endothelial clusters, *CXCL12*/*COL3A1* for MSC/Fibro, *ALPL* for osteochondral/fibroblast cells.

Supplementary Figure 5 (relating to Figure 4)

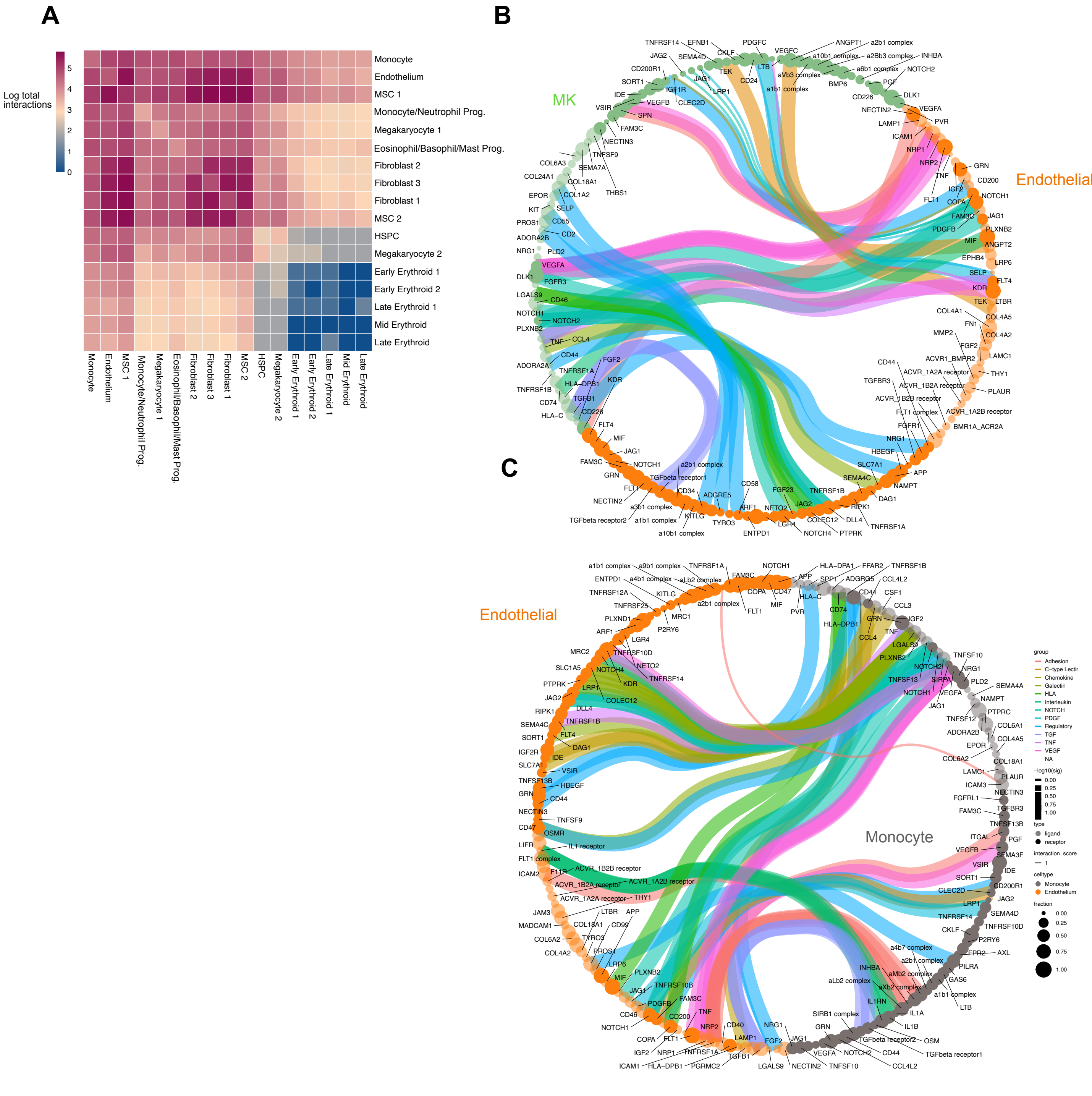

Supplementary Figure 5, relating to Figure 4: Cell-cell interactions between hematopoietic and stromal cell types in VEGFA+C-stimulated organoids. (**A**) Heatmap showing the log(number) of significant predicted receptor (R) – ligand (L) interactions between cell clusters identified using CellPhoneDB v2.0.1. (**B & C**) Circos plots showing interactions between **(B)** megakaryocytes and **(C)** monocytes with endothelial cells. Colors indicate R-L group, width of connecting band reflects log(10) p value and the size of the circle indicates the percentage of cells within the cluster that expressed the relevant receptor or ligand. Interactions were color coded by grouping, and only significant interactions were plotted.

Supplementary Figure 6 (relating to Figure 4)

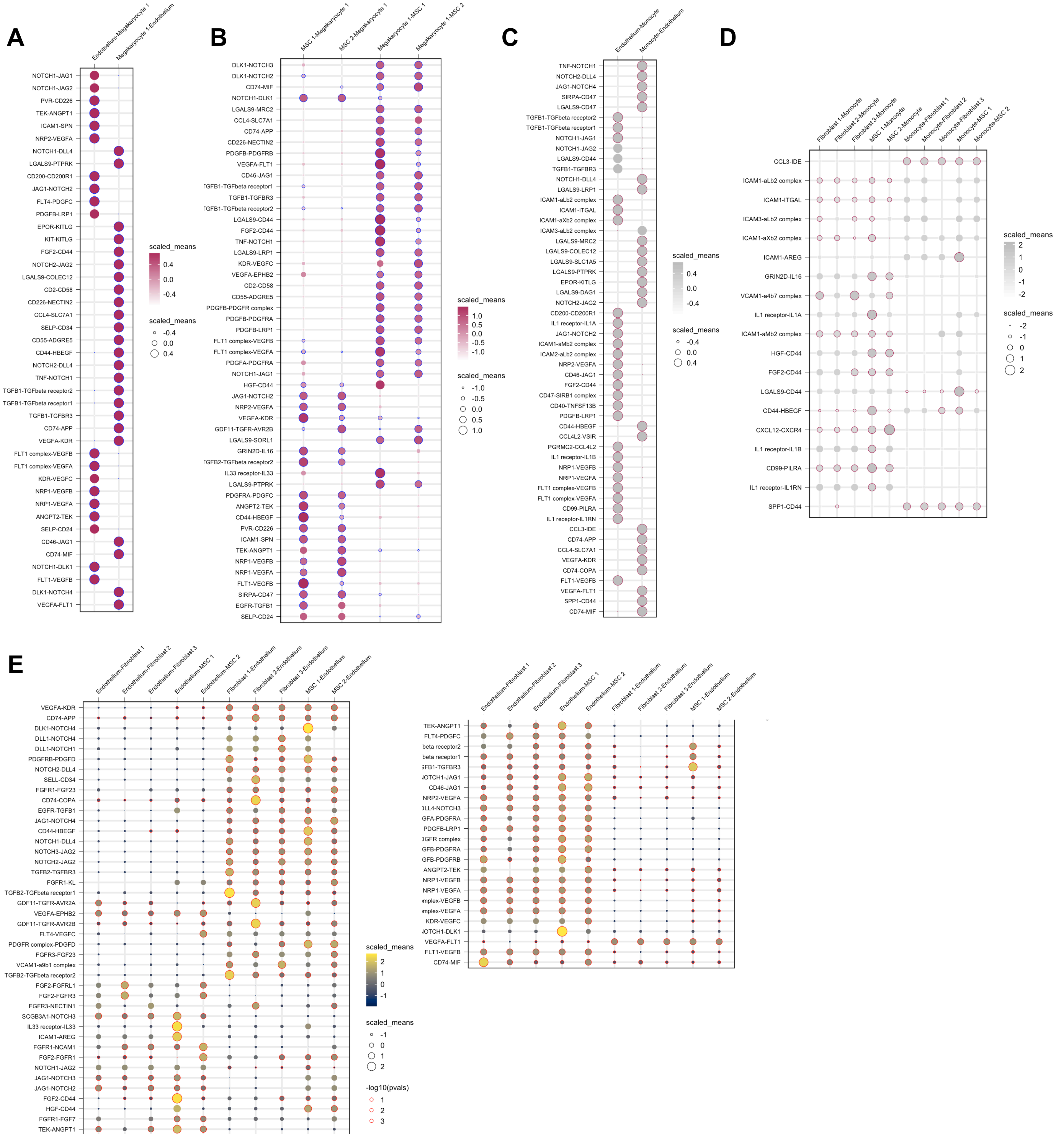

Supplementary Figure 6, relating to Figure 4: Dot plots showing significant receptor-ligand interactions between key interacting cell types within the organoids. Significant interactions predicted by CellPhoneDB (p value < 0.05) are shown for selected clusters to highlight interactions of interest. Each circle is color and size coded to indicate relevant expression level (scaled means) for the ligand:receptor pairing in the clusters of interest. **(A)** Significant endothelial cell – megakaryocyte; **(B)** mesenchymal stromal cell (MSC) - megakaryocyte; **(C)** endothelial cell – monocyte; **(D)** fibroblast/MSC – monocyte and **(E)** endothelial cell – MSC/fibroblast interacting ligand-receptor pairs.

Supplementary Figure 7 (relating to Figure 3)

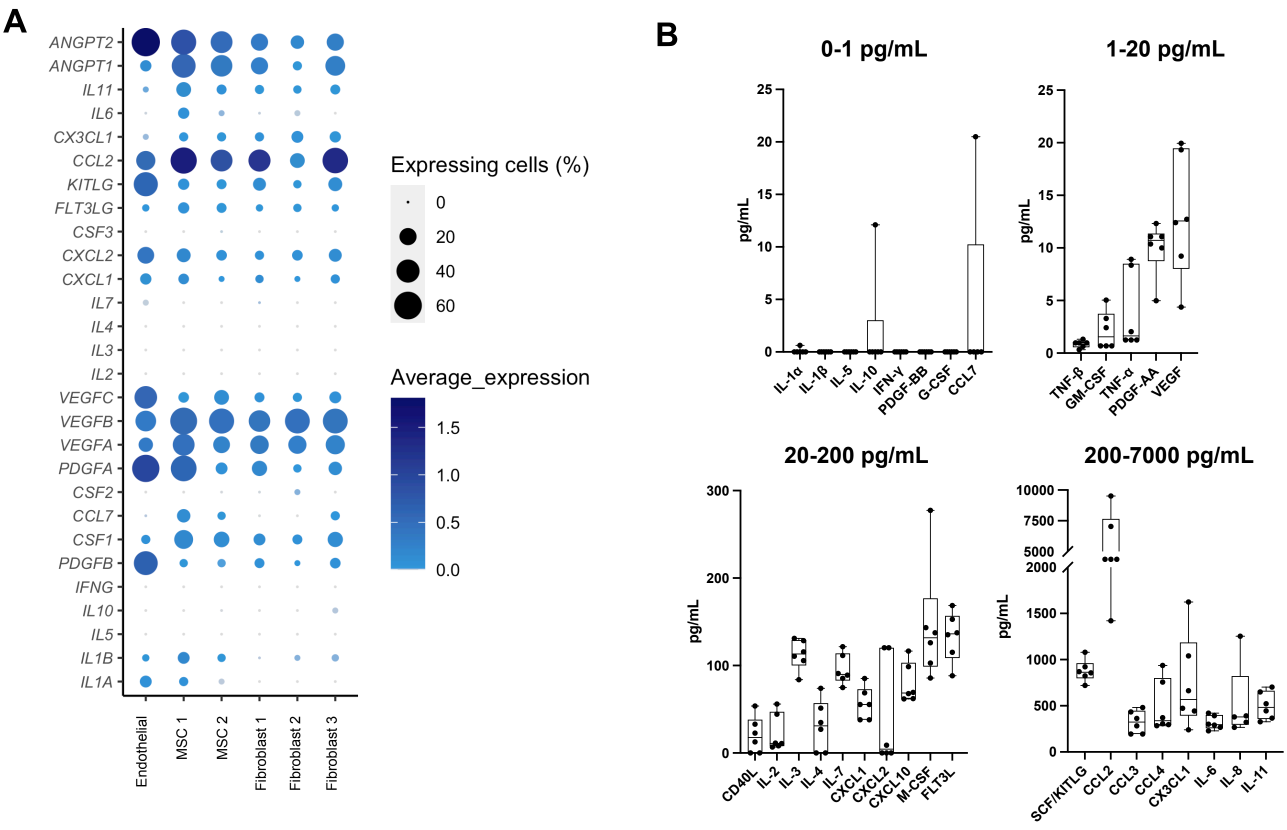

Supplementary Figure 7, relating to Figure 4: Expression of hematopoietic support factors by bone marrow organoids. (A) Expression of hematopoietic growth factors in stromal cell subsets detected by scRNAseq. **(B)** Detection of hematopoietic support factors at the protein level in the organoid media following serial 'washouts' to remove exogenously added cytokines. 50:50 media changes were performed every 72 hours, and media collected at day 12 for Luminex assay. Charts are split according to expression level of proteins.

Supplementary Figure 8 (relating to Figure 6)

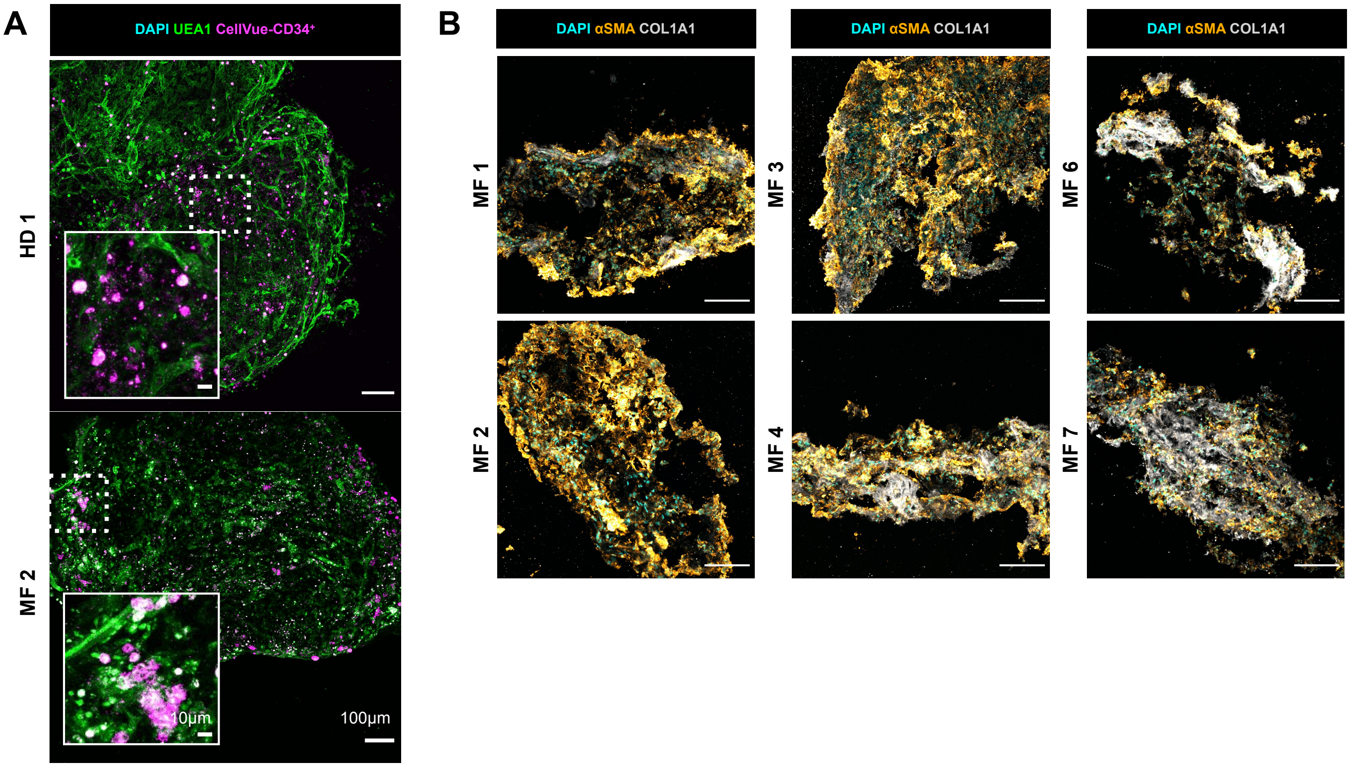

Supplementary Figure 8, relating to Figure 6: Organoids engrafted with CD34+ hematopoietic stem/progenitor cells from healthy donors and patients with myelofibrosis demonstrate fibrotic remodelling. (A) Fluorescently labelled healthy donor and patient cells visualized within organoids in whole volume confocal images (maximum Z projection). Adult donor-derived cells were observed throughout the volume of the organoid. **(B)** Increased collagen 1 deposition and αSMA expression 14 days after seeding of organoids with cells from patients with myelofibrosis.

Supplementary Figure 9 (relating to Figure 6)

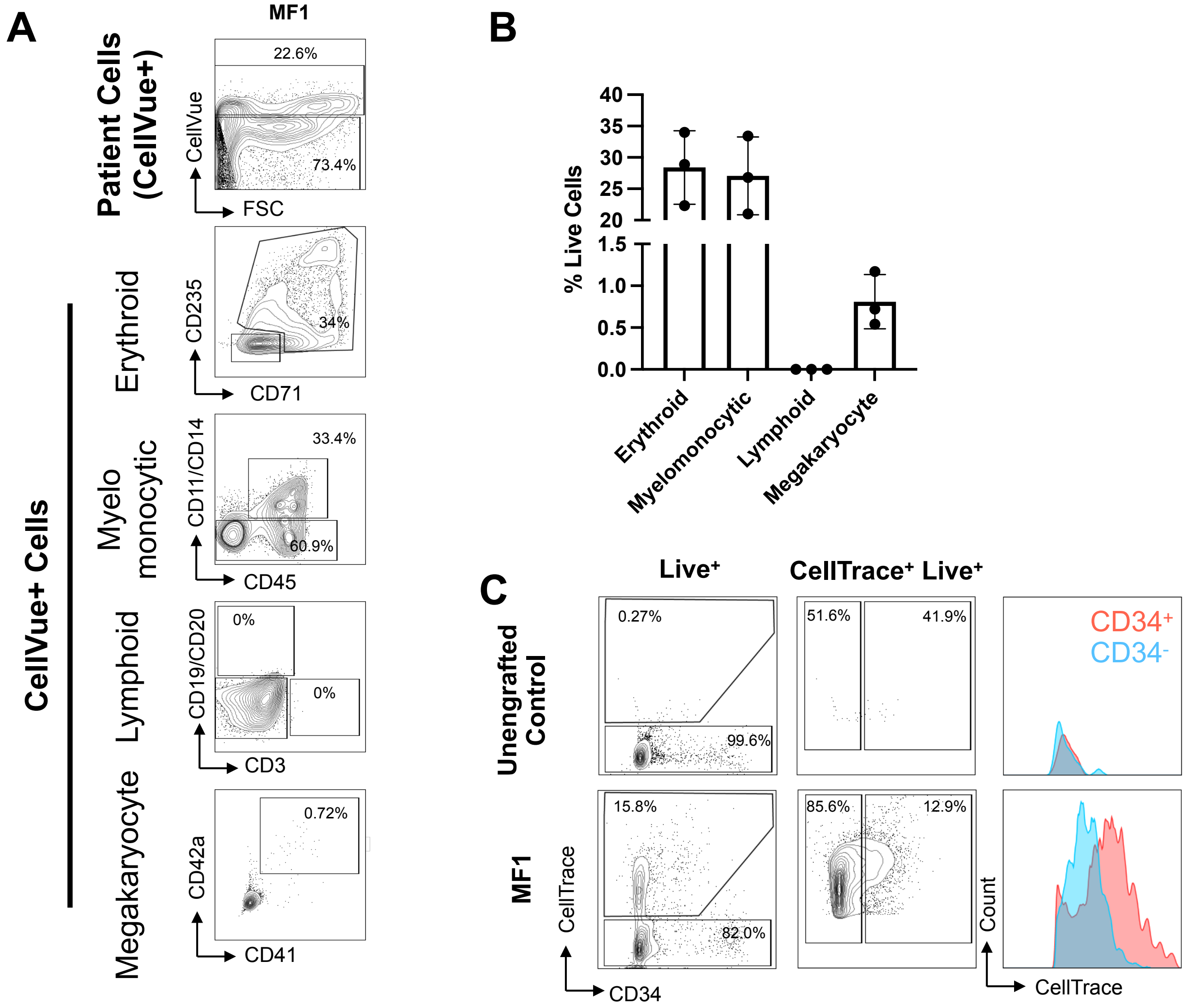

Supplementary Figure 9, relating to Figure 6: CD34+ hematopoietic stem/progenitor cells from patients with myelofibrosis engrafted onto bone marrow organoids differentiate into myeloid lineages and retain a quiescent CD34+ population after 14 days of culture. (A,B) Within the label-positive cells at day 14, populations of CD71+/CD235+ erythroid, CD11b/CD14+ myelomonocytic, and CD41+/CD42+ megakaryocytic cells were detected. **(C)** Distinct CellTrace+ CD34+ cells were detected in organoids. The left shift in CellTrace peak in CD34- cells reflects lower rates of cell division for the CD34+ cells.

Supplementary Figure 10 (relating to Figure 7)

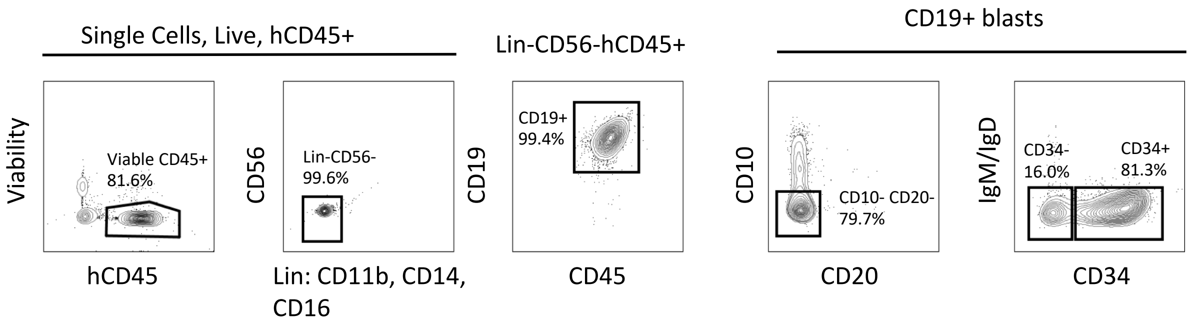

Supplementary Figure 10, relating to Figure 7: Immunophenotyping of donor Xeno iALL cells prior to engraftment on to BM organoids. Xeno iALL samples were confirmed as 99% lymphoblasts by confirmation of CD45$^{+,}$ CD19^{+} , with majority of ProB phenotype (CD10-CD20-IgM/IgD-) and variable expression of CD34. Representative plot shown.

Supplementary Figure 11 (relating to Figure 7)

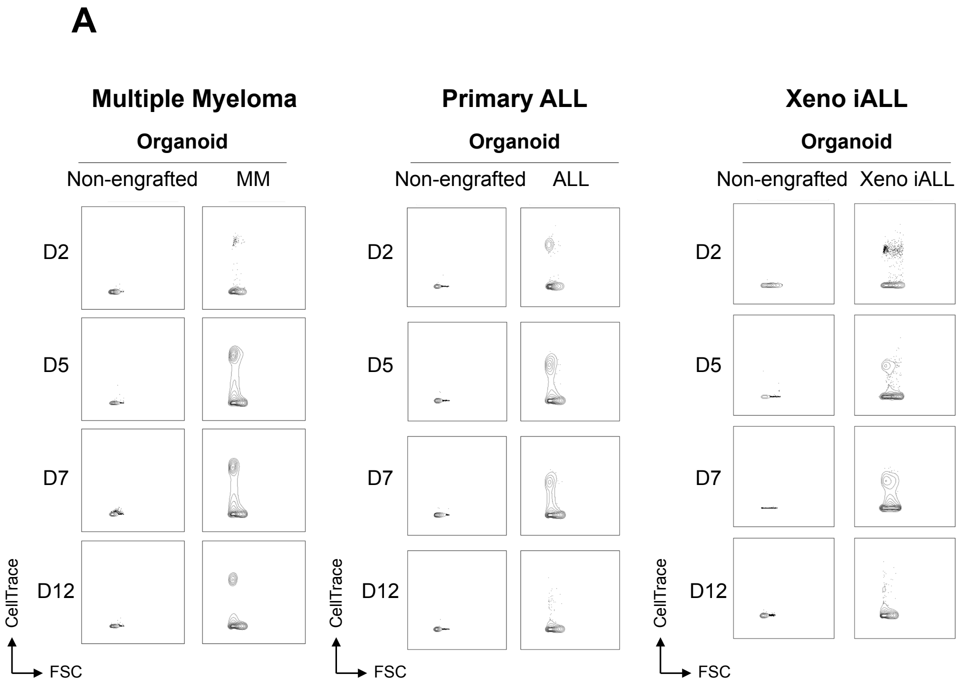

Supplementary Figure 11, relating to Figure 7: Detection of CellTrace+ cells in organoids over 12 days after seeding. (A) Compared to non-engrafted controls, a distinct CellTrace+ population was evident in all experiments. The CellTrace signal is lost after 7-8 cell divisions, or following cell death.

Supplementary Figure 12 (relating to Figure 7)

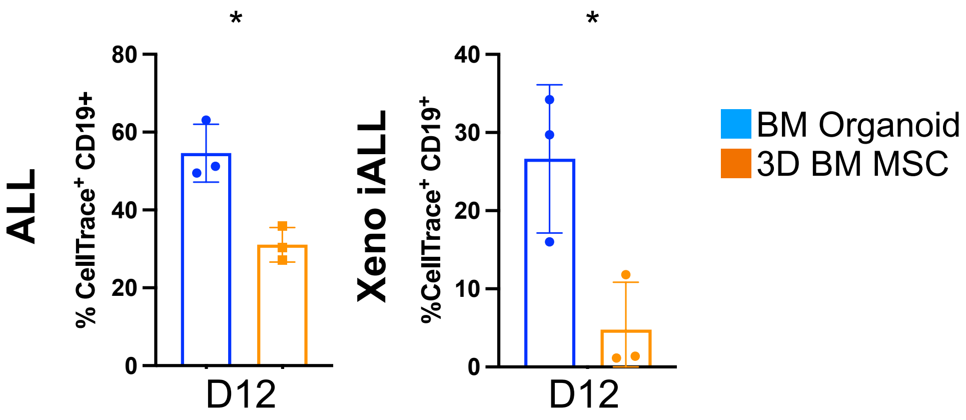

Supplementary Figure 12 (Relating to figure 7): CD19 expression on ALL and iALL in model B vs organoids after 12 days of co-culture. ALL and Xeno iALL cells assessed for the retention of phenotype (CD19+ CellTrace+ cells) after 12 days of co-culture. In both instances, cells co-cultured with BM organoids demonstrate a significantly increased CellTrace+ CD19+ population. (* $p = < 0.05$, paired t-test, *n* = 3 donors/samples each.
